# Borylation and rearrangement of alkynyloxiranes: a stereospecific route to substituted α-enynes

**DOI:** 10.3762/bjoc.15.141

**Published:** 2019-06-27

**Authors:** Ruben Pomar Fuentespina, José Angel Garcia de la Cruz, Gabriel Durin, Victor Mamane, Jean-Marc Weibel, Patrick Pale

**Affiliations:** 1 Laboratoire de Synthèse, Réactivité Organiques & Catalyse, associé au CNRS, Institut de Chimie, Université de Strasbourg, 4 rue Blaise Pascal, 67000 Strasbourg, France

**Keywords:** boron, enyne, lithium, oxirane, rearrangement

## Abstract

1,3-Enynes are important building blocks in organic synthesis and also constitute the key motif in various bioactive natural products and functional materials. However, synthetic approaches to stereodefined substituted 1,3-enynes remain a challenge, as they are limited to Wittig and cross-coupling reactions. Herein, stereodefined 1,3-enynes, including tetrasubstituted ones, were straightforwardly synthesized from *cis* or *trans*-alkynylated oxiranes in good to excellent yields by a one-pot cascade process. The procedure relies on oxirane deprotonation, borylation and a stereospecific rearrangement of the so-formed alkynyloxiranyl borates. This stereospecific process overall transfers the *cis* or *trans*-stereochemistry of the starting alkynyloxiranes to the resulting 1,3-enynes.

## Introduction

Lithiated oxiranes are useful intermediates in organic synthesis [1–2]. Such species can undergo different reactions, driven either by their carbanionic or carbenic behavior [3–4], thus leading to various products. In contrast, the transmetalation of lithiated oxiranes and the evolution of the resulting species have been less explored. Utimoto’s group was probably the first who reported on the treatment of oxiranyllithium with diethylzinc or trimethylaluminium [5]. Shimizu et al. demonstrated that in situ formed trifluoromethyloxiranyllithium derivatives reacted with trimethylaluminium, dimethylzinc or organoboranes to yield tetrasubstituted alkenes [6–7]. Capitalizing on these precedents, Aggarwal et al. showed that oxiranes could be homologated to diols through lithiation, borylation, rearrangement and oxidation of the so-formed β-hydroxyboranes [8]. More recently, Blakemore et al*.* applied this sequence to sulfinyloxiranes [9]. Alternatively, Unemaya et al. and more recently Buchwald et al*.* described a Negishi cross coupling of aryl bromides or chlorides to α-CF_3_-oxiranyl zincates generated by lithiation of trifluoromethyloxirane and transmetalation with zinc chloride [10–11].

**Scheme 1:**
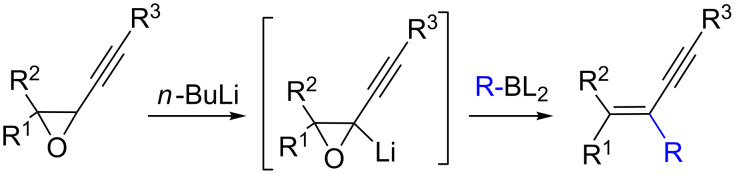
Stereospecific formation of α-enynes from alkynyloxiranes.

In this context and based on the pioneering work of Shimizu [6–7], and our experience on the metalation of alkynyloxiranes and reactivity studies of the so-formed alkynyloxirane anions [12–13], we explored the reaction of such anions with various boron derivatives in order to produce stereodefined α-enynes after Matteson-type rearrangement [14] and elimination [15] (Scheme 1). We also demonstrated the stereospecificity of these transformations [16].

α-Enynes (1,3-enynes) are an interesting class of compounds in organic chemistry as these structures are found in various pharmacologically active natural products [17], and in functional materials [18]. α-Enynes are also important building blocks in organic syntheses or platforms for further synthesis [19]. Accordingly, significant efforts have been made to develop efficient syntheses of enynes [17–20]. The most common routes relied on Wittig-type reactions [21–22] and transition-metal-catalyzed coupling reactions of either terminal or organometallic alkynes with vinyl halides or of alkynyl halides with vinylic organometallics [20]. However, both routes may lead to mixtures of stereoisomers and synthetic approaches to stereodefined substituted α-enynes remain scarce and thus still represent a challenge in organic chemistry.

## Results and Discussion

### Reaction set up

First we selected the simple 2-ethynyl-3-dimethyloxirane **1a** to probe the feasibility of the reaction. In order to avoid unwanted reactions upon metalation [23], the ethynyl group was silylated and a bulky silyl group was installed to avoid any silyl migration [12]. As already shown [12–13], *n*-butyllithium proved to be very efficient, although stronger or complex bases are usually required to produce oxiranyl anions [1–2,24–28]. Upon deprotonation, the corresponding oxiranyllithium was sufficiently stable at −78 °C (see below) to be trapped by various phenylboronic esters **2**. The resulting borate intermediate afforded the tetrasubstituted enyne **3** in good to moderate yields, depending on the boronic ester’s nature (Table 1). The catechol ester **2a** only provided a modest yield of **3** (Table 1, entry 1), while the neopentylglycol ester **2b** proved slightly better (Table 1, entry 2). The pinacol ester **2c** turned out to be the most effective reagent for this transformation, giving around 70% of **3** after isolation and purification (Table 1, entry 3). These results showed that the steric bulk at the boron atom seems to facilitate the reaction. Indeed, the bulkier the boron substituent is, the more efficient is the process. Therefore, the substitution at the boron plays a key role, most probably at the boron rearrangement step (see below).

**Table 1:** Borylation and rearrangement of ethynyloxirane **1a**.^a,b^.

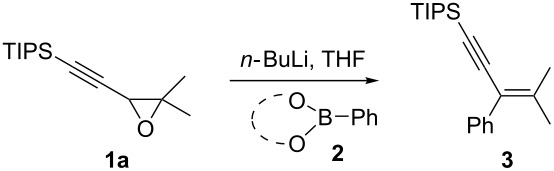				

Entry	Boronic ester	Time^c^ (min)		Yield^d^ (%)
		*t*_1_	*t*_2_	

1	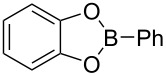 **2a**	30	20	33
2	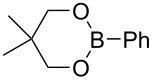 **2b**	30	20	43
3	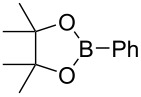 **2c**	30	20	66

^a^Reaction conditions: 1) 1.5 equiv *n-*BuLi, THF, −78 °C, *t*_1_; 2) 1.5 equiv boronic ester **2**, −78 °C, *t*_2_; 3) rt, 1 h; ^b^TIPS: triisopropylsilyl); ^c^*t*_1_ corresponds to the time of the lithiation step and *t*_2_ to the time of the borylation step; ^d^yields of pure isolated products, unless otherwise stated.

### Stereoselectivity, scope and limitations

Next, the stereoselectivity of this rearrangement was evaluated using *cis* and *trans*-disubstituted alkynyloxiranes (Table 2). For synthetic reasons [29–30], we selected *cis*-2,3-epoxy-5-triisopropylsilylpent-4-ynol, protected with the monomethoxytrityl group (MMTr) **1b** and prepared its *trans*-isomer to compare the outcome of the reaction with both isomers.

**Table 2:** Lithiation, borylation and rearrangement of *cis* or *trans*-disubstituted ethynyloxiranes **1b**.^a,b^.

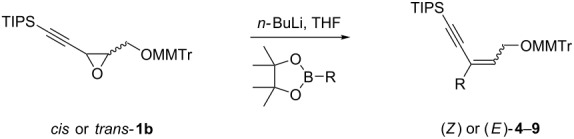							

Entry	Oxirane	R	Temp. (°C)	Time^c^ (min)		Enyne	Yield^d^ (%)
				*t*_1_	*t*_2_		

1	*cis***-1b**	Ph	−78	30	20	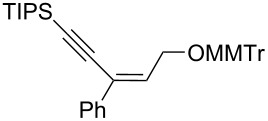 (*Z*)-**4**	78
2	*trans-***1b**	"	−78	30	20	*(E)-***4** + (*Z*)-**4** 4:1	80
3	*trans-***1b**	"	−92	60	90	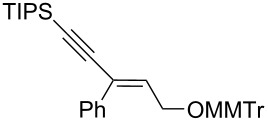 (*E*)-**4**	70
4	*cis***-1b**	*p-*MeOPh	−78	30	20	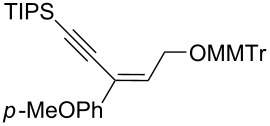 (*Z*)-**5**	85
5	*trans-***1b**	"	−92	60	90	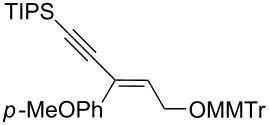 (*E*)-**5**	88
6	*cis***-1b**	*p-*ClPh	−78	30	20	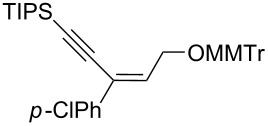 (*Z*)-**6**	81
7	*trans-***1b**	"	−92	60	90	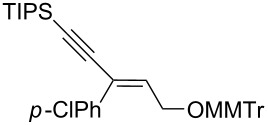 (*E*)-**6**	85
8	*cis***-1b**	Bn	−78	30	20	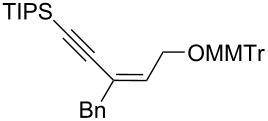 (*Z*)-**7**	62
9	*trans-***1b**	"	−92	60	90	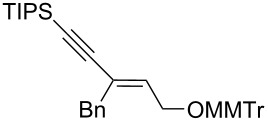 (*E*)-**7**	65
10	*cis***-1b**	allyl	−78	30	20	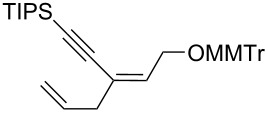 (*Z*)-**8**	57
11	*trans-***1b**	"	−92	60	90	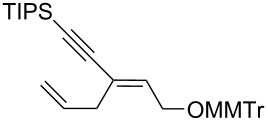 (*E*)-**8**	78
12	*cis***-1b**	*n-*butyl	−78	30	20	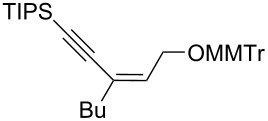 (*Z*)-**9**	85
13	*trans-***1b**	"	−92	60	90	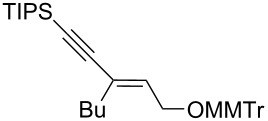 (*E*)-**9**	90

^a^Reactions were run under argon, *c* = 0.125 mol/L; ^b^MMTr: monomethoxytrityl; ^c^*t*_1_ corresponds to the time of the lithiation step and *t*_2_ to the time of the borylation step; ^d^yields of pure isolated products, unless otherwise stated.

The *cis*-configured compound *cis*-**1b** readily reacted with pinacol phenylboronate **2c** under the conditions set up above, providing enyne **4** as the sole product in high yield (Table 2, entry 1). NMR analysis revealed the *Z-*stereochemistry of this enyne [31]. In contrast, the *trans-*isomer *trans*-**1b** afforded a mixture of the 2 possible isomers, although in similar overall yield (Table 2, entry 2 vs 1). Decreasing the temperature from −78 to −92 °C for the deprotonation–borylation steps rewardingly allowed the exclusive formation of the *E-*enyne **4** as a single isomer (Table 2, entry 3).

After these encouraging results, the nature of the group transferred during the rearrangement was explored. Electron-withdrawing or electron-donating substituents on the phenyl group did not alter the stereochemical course of the reaction, while slightly improving the yields (Table 2, entries 4–7 vs 1 and 2). Benzyl or allyl groups could be transferred as well, although slightly lower yields were observed (Table 2, entries 8–11). Even an alkyl group could be transferred and interestingly, the best yields were achieved with this kind of group (Table 2, entries 12 and 13).

It is worth noting that the same stereochemical relationship between the starting oxirane and the enyne product was always obtained, whatever the group transferred from boron to the oxirane carbon. These results revealed the stereospecificity of the reaction and suggested that no epimerization of the oxiranyllithium intermediate occurred.

The present reaction was then further explored and extended to various ethynyloxiranes (Table 3). The non-functionalized *cis* or *trans*-3,4-epoxy-1-triisopropylsilyldec-1-ynes **1c** stereospecifically gave the expected *E* or *Z* trisubstituted enynes **10** in moderate to good yields (Table 3, entries 1 and 2). It is worth mentioning that *cis*-**1c** contaminated with its *trans-*isomer (9:1 ratio) was converted to enyne **10** with the same *E/Z* ratio of 1:9. The *cis* or *trans*-phenyl analogues **1d** were less reactive, as revealed by incomplete reactions at −92 °C and longer reaction times needed at −78 °C. Surprisingly, the *trans* isomer of **1d** always provided a 4:1 mixture of the *E/Z* isomers of **11**, while *cis-***1d** gave the expected *Z-*isomer of **11** exclusively (Table 3, entries 3 and 4). The two isomers could, however, be separated by chromatography. Interestingly, the *cis* or *trans*-epoxydiynes **1e** stereospecifically and solely provided the corresponding and expected *E* or *Z-*isomers of enyne **12** in quite good yields (Table 3, entries 5 and 6). However, in these reactions some degradation was observed, but the yields were satisfying considering the sensitive polyunsaturated nature of the starting compounds. The trisubstituted ethynyloxirane **1f** proved also reactive, but longer reaction times were required, probably due to steric hindrance at some stage of the rearrangement (vide infra). Both isomers exclusively afforded the expected *E* or *Z* tetrasubstituted alkenes **13** in high yields (Table 3, entries 7 and 8).

**Table 3:** Lithiation, borylation and rearrangement of various *cis* or *trans*-ethynyloxiranes.^a^

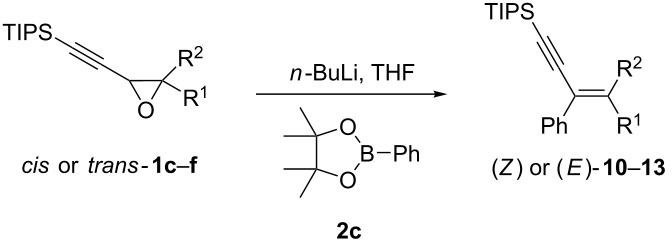				

Entry	Oxirane	Temp. (°C)	Enyne	Yield (%)^b^

1^c^	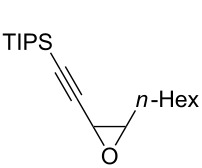 *cis*-**1c**	−78	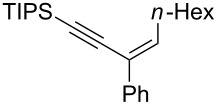 (*Z*)-**10**	55^d^
2	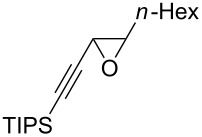 *trans*-**1c**	−92	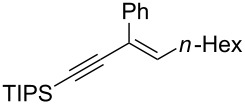 (*E*)-**10**	56
3	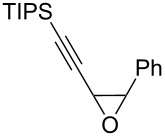 *cis*-**1d**	−78	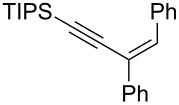 (*Z*)-**11**	48
4	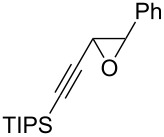 *trans*-**1d**	−78	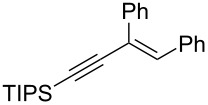 (*E*)-**11**	63^e,f^
5	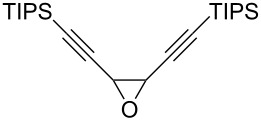 *cis*-**1e**	−78	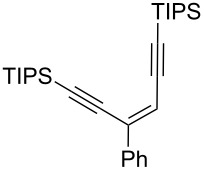 (*Z*)-**12**	59^g^
6	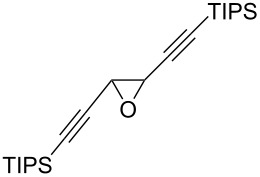 *trans*-**1e**	−92	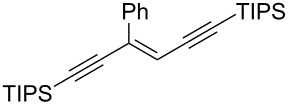 (*E*)-**12**	57^g^
7	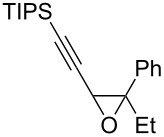 *cis*-**1f**	−78	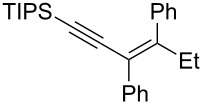 (*Z*)-**13**	69^h^
8	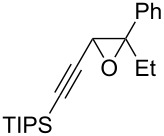 *trans*-**1f**	−92	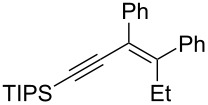 (*E*)-**13**	73^h^

^a^Reactions were run under argon, *c* = 0.125 mol/L; ^b^yields of pure isolated products, unless otherwise stated; ^c^ratio *cis*/*trans* 9:1; ^d^ratio *Z*/*E* 9:1; ^e^a 4:1 mixture of stereoisomers was obtained; ^f^the yield refers to the isolated *E-*isomer; ^g^some degradation occurred; ^h^after addition of the boronic ester, the mixture was stirred overnight at room temperature.

### Stereochemistry and mechanism

Based on the above results, we conclude that the alkynyloxirane-to-enyne reaction is stereospecific, maintaining the stereochemistry of the starting oxirane in the resulting enyne. This stereospecificity imposes the stereochemical integrity of the oxiranyllithium species formed upon lithiation, but also the existence of at least one concerted step during the reaction mechanism. This step most probably corresponds to the group transfer in organoboron derivatives, as the stereoselectivity of such transfer has been demonstrated [32–33].

**Scheme 2:**
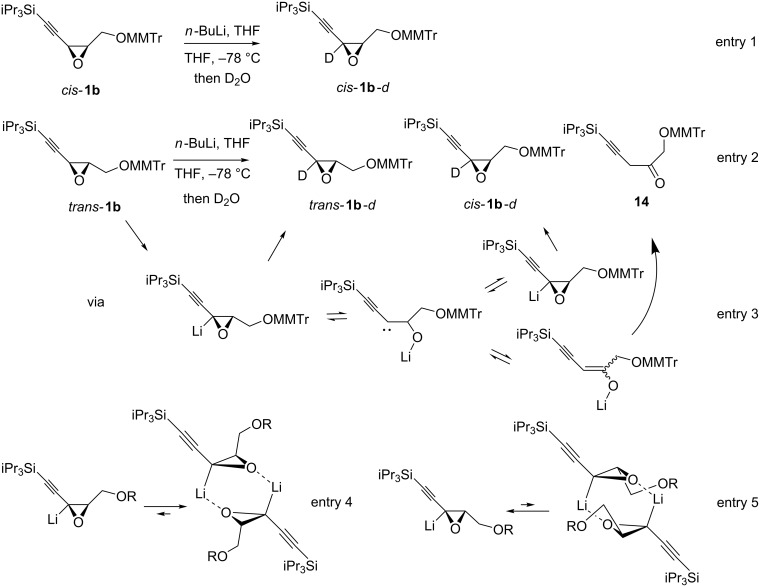
Trapping experiments of the oxiranyllithium derived from *cis* or *trans*-alkynyloxiranes **1b**, and their interpretation.

Regarding the oxiranyllithium stereochemical integrity and stability, it has been demonstrated that solvent, oxirane substituent’s nature and of course temperature play key roles [34], in addition to the initial *cis* or *trans* stereochemistry, if present [35]. In our case, the oxiranyllithium derived from *cis*-alkynyloxiranes are configurationally stable as it was established by us [12–13]. However, we found here that in case of *trans*-alkynyloxiranes, a lower temperature was required to ensure stereospecificity (see entry 3 vs 2, Table 2). This observation suggests that the corresponding oxiranyllithium species could be labile. Trapping experiments with deuterated water were thus conducted at −92 and −78 °C with *cis*-**1b** or *trans*-**1b**, respectively. As expected, the *cis-*isomer gave the deuterated *cis-*derivative **1b**-*d* with an excellent 92% yield, whatever the temperature was (Scheme 2, entry 1). However, the *trans-*isomer at −78 °C, produced a mixture of products (Scheme 2, entry 2). The expected deuterated *trans-*derivative was the major compound, the second product (2:1 ratio) was ascribed to ketone **14** after isolation. In addition, traces of the deuterated *cis-*derivative were detected by ^1^H NMR in the crude mixture, together with traces of other side-products. The formation of this set of products clearly evidenced the carbenic behavior of the involved oxiranyllithium (Scheme 2, entry 3) and revealed that the *trans*-alkynyloxiranes are unstable at −78 °C, but perfectly stable at −92 °C.

As already proposed [36] and then demonstrated [12–13], deprotonation of oxiranes occurred upon oxygen-directed metalation. The resulting oxiranyllithium probably exists as a dimer in solution, as it has been nicely evidenced by structural characterization and NMR studies of the lithiated *o*-trifluoromethylstyrene oxide [37]. Such dimerization obviously stabilizes the oxiranyllithium species. Applied to oxiranyllithium derived from *cis* or *trans*-alkynyloxiranes, such dimer may easily form from the *cis-*oxirane (Scheme 2, entry 4), but not from the *trans-*isomer, or at least the large steric constrain present in it may hamper its stability. Indeed, the long oxirane C–O bond adjacent to lithium observed by X-ray diffraction in the lithiated *o*-trifluoromethylstyrene oxide [37] may be further elongated due to hindrance introduced by the *trans-*substituent adjacent to the lithium atom of the second oxirane in the dimer (Scheme 2, entry 5). Such effect may facilitate the formation of the corresponding carbenoid, especially with increasing temperature.

**Scheme 3:**
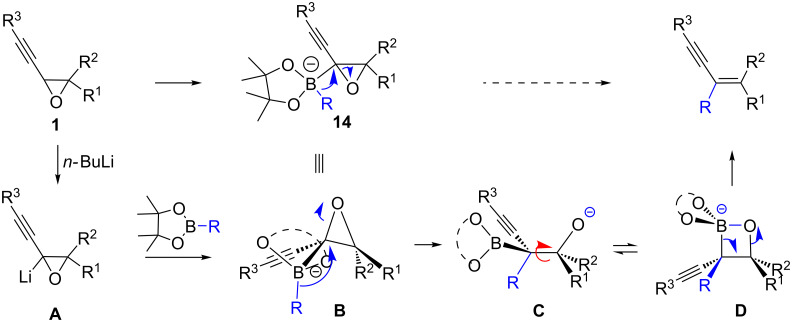
Proposed mechanism for the rearrangement of alkynyloxiranes to α-enynes through metalation and borylation.

Once the oxiranyllithium species is formed (**A** in Scheme 3), oxiranylborate complexes **B** are obtained by the addition of aryl-, benzyl-, allyl- or alkylboronic esters. This was clearly evidenced by the fast disappearance of the coloration linked to the oxiranyllithium species upon the addition of the boronic ester. Unfortunately, all attempts to detect or isolate such ate complexes or related oxiranylboronic esters failed so far [38]. The oxiranylborate complex **B** then undergoes an intramolecular S_N_2-like process (Matteson-type rearrangement) [14] transferring the non-oxygenated boron substituent and promoting oxirane ring opening [39]. The group’s migratory aptitude cannot be directly compared to that known from cationic rearrangements, because the nature of the other boron substituents plays a key role in migration, as revealed by calculations [40–42]. From the results we so far gained, a migratory order could be assessed for the present reaction: Bu ≥ EDG-substitued Ar > EWG-substitued Ar ≥ Ar ≥ benzyl > allyl, and it appears that the larger the other substituents on the boron atom are, the easier the transfer is.

A possible transition state **B** could be envisaged with the large pinacol substituent facing the small oxirane ring and thus placing the migrating group antiparallel to the adjacent oxiranyl C–O bond. This may explain the role of the diol substituent on the reaction efficacy (see Table 1). Upon migration and oxirane ring opening, a β-boron alkoxide **C** is formed, which probably isomerizes to a 1,2-oxaboretanide **D**, a known intermediate in bora-Wittig reactions [7], whose fragmentation then stereospecifically forms the observed enyne.

## Conclusion

To summarize, we reported here on a convenient access to stereochemically defined α-enynes through lithiation, borylation and rearrangement of *cis* or *trans*-alkynyloxiranes. This cascade reaction is stereospecific, probably due to the stereocontrolled group transfer from boron to the adjacent oxiranyl carbon in an oxiranylborate complex intermediate, followed by an internal bora-Wittig reaction.

This method is particularly appealing, providing with good to high yields a wide diversity of functionalized *E* or *Z* tri- or tetrasubstituted α-enynes.

## Supporting Information

File 1 Experimental details and analytical data of all compounds.

File 2 Copies of ^1^H NMR and ^13^C NMR spectra of all new compounds.
