# Supplementary material for: Borylation and rearrangement of alkynyloxiranes: a stereospecific route to substituted α-enynes
Source: Beilstein J Org Chem. 2019 Jun 27;15:1416–24. doi: 10.3762/bjoc.15.141 (PMC6604752; doi:10.3762/bjoc.15.141)
Supplement: File 1 — Experimental details and analytical data of all compounds. [file Beilstein_J_Org_Chem-15-1416-s001.pdf]

BEILSTEIN JOURNAL OF ORGANIC CHEMISTRY

Supporting Information

for

Borylation and rearrangement of alkynyloxiranes: a stereospecific route to substituted α-enynes

Ruben Pomar Fuentespina, José Luis Angel Garcia de la Cruz, Gabriel Durin, Victor Mamane, Jean-Marc Weibel and Patrick Pale

Beilstein J. Org. Chem. **2019,** *15,* 1416–1424. doi:10.3762/bjoc.15.141

Experimental details and analytical data of all compounds

License and Terms: This is a supporting information file under the terms of the Creative Commons Attribution License (http://creativecommons.org/licenses/by/4.0). Please note that the reuse, redistribution and reproduction in particular requires that the authors and source are credited.
The license is subject to the *Beilstein Journal of Organic Chemistry* terms and conditions: (https://www.beilstein-journals.org/bjoc)

Experimental

General information. Proton (^{1}H NMR) and carbon (^{13}C NMR) nuclear magnetic resonance spectra were recorded on 300, 400 or 500 MHz instruments. The chemical shifts are given in parts per million (ppm) on the delta scale. The residual solvent peak was used as reference. For ^{1}H NMR: $CDCl_3$= 7.26 ppm; for ^{13}C NMR: $CDCl_3$= 77.16 ppm. Data are presented as follows: chemical shift, multiplicity (s = singlet, d = doublet, t = triplet, q = quartet, quint = quintet, m = multiplet, b = broad), coupling constants (J/Hz) and integration. High resolution mass spectra (HRMS) data were recorded on a microTOF spectrometer equipped with orthogonal electrospray interface (ESI). Analytical thin layer chromatography (TLC) was carried out on silica gel 60 F254 plates with visualization by ultraviolet light. Reagents and solvents were purified using standard procedures. Tetrahydrofuran (THF) was freshly distilled from sodium metal/benzophenone. Anhydrous reactions were carried out in flame-dried glassware and under an argon atmosphere. All other chemicals were used as received.

Synthesis of substrate 1a

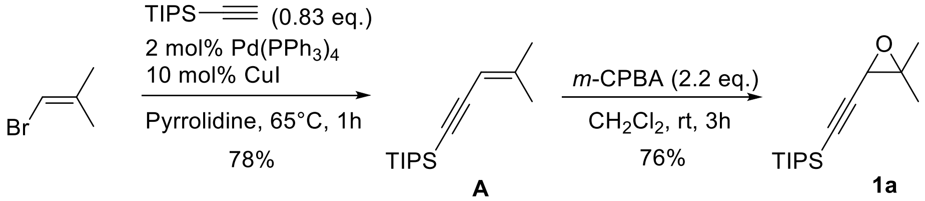

Triisopropyl(4-methylpent-3-en-1-yn-1-yl)silane (A). To a solution of 1-bromo-2-methylprop-1-ene (1.1 mL, 10.7 mmol) in degassed pyrrolidine (10 mL) at ambient temperature were successively added $Pd(PPh_3)_4$ (2 mol %, 205 mg, 0.178 mmol), CuI (10 mol %, 170 mg, 0.89 mmol) and ethynyltriisopropylsilane (2.0 mL, 8.9 mmol). The mixture was placed in a pre-heated bath at 65 °C and stirred for 1 h. After cooling to ambient temperature, a saturated solution of NH_4Cl (30 mL) was added. The mixture was extracted with Et_2O (3 × 30 mL). The organic phases were combined, washed with brine (30 mL), dried over Na_2SO_4, filtered and concentrated under vacuum. The crude was purified by flash chromatography on silica gel (petroleum ether) to give 1.64 g of a colorless liquid in 78% yield. ^{1}H NMR (500 MHz, $CDCl_3$) δ 5.33 (m, 1H), 1.93 (s, 3H), 1.81 (d, J = 2.0 Hz, 3H), 1.10-1.07 (m, 21H). ^{13}C NMR (126 MHz, $CDCl_3$) δ 150.1, 106.0, 105.3, 92.3, 25.0, 21.3, 18.8, 11.5.

((3,3-Dimethyloxiran-2-yl)ethynyl)triisopropylsilane (1a). Compound **A** (675 mg, 2.86 mmol) was dissolved in dichloromethane (3 mL) and placed in an ice bath. A solution of *m*-CPBA (77% purity, 1.41 g, 6.29 mmol) in dichloromethane (16 mL) was added dropwise through an addition funnel over a period of 1 h. The ice bath was removed and the reaction mixture was stirred vigorously for 3 h. The reaction was quenched with 10% sodium thiosulfate (30 mL) and extracted with diethyl ether (3 × 30 mL). The combined organic layers were then washed with a saturated solution of sodium bicarbonate (3 × 30 mL). The organic phase was dried with sodium sulfate and concentrated under reduced pressure. The crude material was further purified using silica gel chromatography (petroleum ether/diethyl ether 95:5) to give 525 mg of a colorless liquid in 76% yield. ^{1}H NMR (500 MHz, $CDCl_3$) δ 3.24 (s, 1H), 1.45 (s,

3H), 1.35 (s, 3H), 1.07 (s, 21H). ^{13}C NMR (126 MHz, $CDCl_3$) δ 103.2, 87.2, 60.7, 52.3, 23.5, 20.4, 18.7, 11.3. HRMS (ESI-TOF) *m/z* $[M + K]^+$ Calcd for $C_{15}H_{28}KOSi$ 291.1541; Found 291.1561.

<u>Synthesis of substrate 1b-*trans*</u>

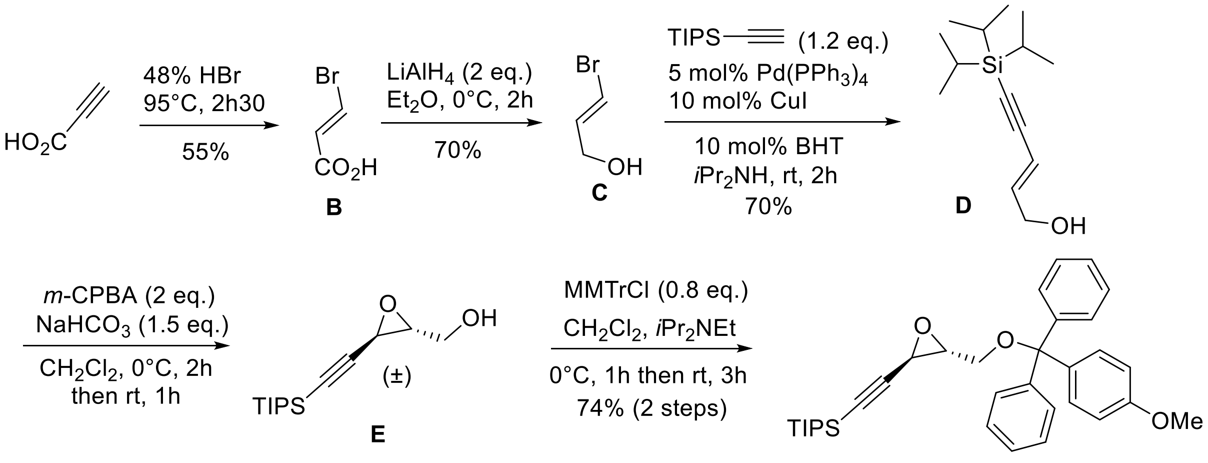

(±)-*trans*-1b

(*E*)-3-Bromoacrylic acid (B).[1] A solution of propargylic acid (5 g, 71.5 mmol) in aqueous HBr 48% (20 mL) was heated at 95 °C and stirred for 2.5 h. After cooling to ambient temperature, the precipitate was filtered, washed with cold water (3 × 12 mL) and dried in air to give 5.85 g of white crystals in 55% yield. ^1H NMR: (300 MHz, $CDCl_3$) δ 7.73 (1H, d, J = 13.8 Hz), 6.52 (1H, d, J = 13.8 Hz).

(*E*)-3-Bromoprop-2-en-1-ol ©.[1] To a suspension of $LiAlH_4$ (1.52 g, 40 mmol) in dry Et_2O (20 mL) at 0 °C was added **B** (3.02 g, 20 mmol) in small portions. After the end of the addition, the mixture was stirred at 0 °C for 2 h. The reaction was quenched at 0 °C by the successive addition of water (1.2 mL), NaOH 15% (3 mL) and water (3.5 mL). The cooling bath was then removed and after 30 min, the mixture was filtered over Celite and washed with Et_2O (50 mL). The filtrate was dried over $MgSO_4$ then concentrated in vacuum to give 1.9 g of a greenish oil in 70% yield. The product was used without further purification in the next step. ^1H NMR: (300 MHz, $CDCl_3$) δ 4.13 (2H, s), 6.38 (2H, m).

(*E*)-5-(Triisopropylsilyl)pent-2-en-4-yn-1-ol (D).[2] In a round-bottomed flask were placed **C** (1.44 g, 10.51 mmol), triisopropylsilylacetylene (2.3 g, 12.61 mmol) and $(iPr)_2NH$ (32 mL). Nitrogen was bubbled into the solution for 20 min. 2,6-Di-*tert*-butyl-*p*-cresol (BHT, 231 mg, 1.05 mmol), $Pd(PPh_3)_4$ (612 mg, 0.53 mmol) and CuI (200 mg, 1.05 mmol) were added and the mixture was stirred at room temperature for 2 h. Then, NH_4Cl sat. (20 mL) was added and the product was extracted with AcOEt (3 × 20 mL). The organic phases were combined, washed with NaCl sat. (3 × 20 mL), then dried over Na_2SO_4. After filtration and concentration under vacuum, the crude product was purified by flash chromatography on silica gel

---

[1] Zeng, F.; Negishi, E.i. *Org. Lett.* **2002**, *4*, 703-706.
[2] Cho, J.; Lee, Y. M.; Kim, D.; Kim, S. *J. Org. Chem.* **2009**,*74*, 3900-3904.

(cyclohexane/AcOEt 6:1) to give 1.76 g of a colorless oil in 70% yield. ^{1}H NMR: (500 MHz, $CDCl_3$) δ 6.29 (dt, *J* = 16.0, 5.2 Hz, 1H), 5.78 (dt, *J* = 16.0, 1.8 Hz, 1H), 4.20 (ddd, *J* = 6.0, 5.2, 1.8 Hz, 2H), 1.06 (s, 21H). ^{13}C NMR (126 MHz, $CDCl_3$) δ 142.6, 111.0, 104.9, 91.8, 63.1, 18.7, 11.4.

***trans*-3-((Triisopropylsilyl)ethynyl)oxiran-2-yl)methanol (E).**[2] To a solution of **D** (3.13 g, 13.1 mmol) in anhydrous DCM (70 mL) was added $NaHCO_3$ (1.67 g, 19.9 mmol) and mCPBA (77%, 6.4 g, 26 mmol) at 0 °C. After stirring for 2 h at 0 °C and 1 h at room temperature, $Na_2S_2O_{3sat.}$ (50 mL) was added. The mixture was washed with $NaCl_{sat.}$ (100 mL) and extracted with DCM (3 × 50 mL). The organic phases were combined and washed with $K_2CO_{3sat.}$ (2 × 50 mL) and dried over $MgSO_4$. After filtration and concentration, the crude product was used in the next step without further purification. ^{1}H NMR (300 MHz, $CDCl_3$) δ 3.96 (dd, *J* = 13.0, 2.1 Hz, 1H), 3.71 (dd, *J* = 13.0, 3.3 Hz, 1H), 3.46 (d, *J* = 2.1 Hz, 1H), 3.31 (dt, *J* = 3.3, 2.1 Hz, 1H), 1.06 (m, 21H).

***trans*-Triisopropyl((3-(((4-methoxyphenyl)diphenylmethoxy)methyl)oxiran-2-yl)ethynyl)silane (*trans*-1b).** To a solution of **D** in DCM (35 mL) was added Hünigs base (4.6 mL). The mixture was cooled to 0 °C and a solution of 4-methoxytriphenylmethyl chloride (MMTrCl, 0.8 equiv, 3.34g, 10.49 mmol) in DCM (18 mL) was added during 30 min. Stirring was continued at 0 °C for 1 h, and at room temperature for 3 h. $NaHCO_3$ sat. (80 mL) was added and the phases were separated. The aqueous phase was extracted with DCM (3 × 35 mL). The organic phases were combined, dried over Na_2SO_4 and concentrated under vacuum. The crude was purified by flash chromatography on silica gel (cyclohexane/AcOEt/Et_3N 98:1:1) to give 2.85 g of a yellow syrup in 74% yield (2 steps). ^{1}H NMR (300 MHz, $CDCl_3$) δ 7.47-7.41 (m, 4H), 7.34-7.18 (m, 8H), 6.83 (d, *J* = 9.0 Hz, 2H), 3.79 (s, 3H), 3.42-3.35 (m, 2H), 3.29 (dt, *J* = 4.8, 2.5 Hz, 1H), 3.14 (dd, *J* = 11.0, 4.5 Hz, 1H), 1.05 (m, 21H). ^{13}C NMR (126 MHz, $CDCl_3$) δ 158.6, 144.5, 136.7, 135.9, 135.8, 130.4, 128.6, 128.5, 127.9, 127.8, 127.0, 126.2, 113.2, 108.2, 89.9, 86.9, 61.6, 55.3, 53.6, 18.8, 11.5. HRMS (ESI-TOF) *m*/*z* $[M + Na]^+$ Calcd for $C_{34}H_{42}NaO_3Si$ 549.2795; Found 549.2796.

<u>Synthesis of substrate 1b-*cis*</u>

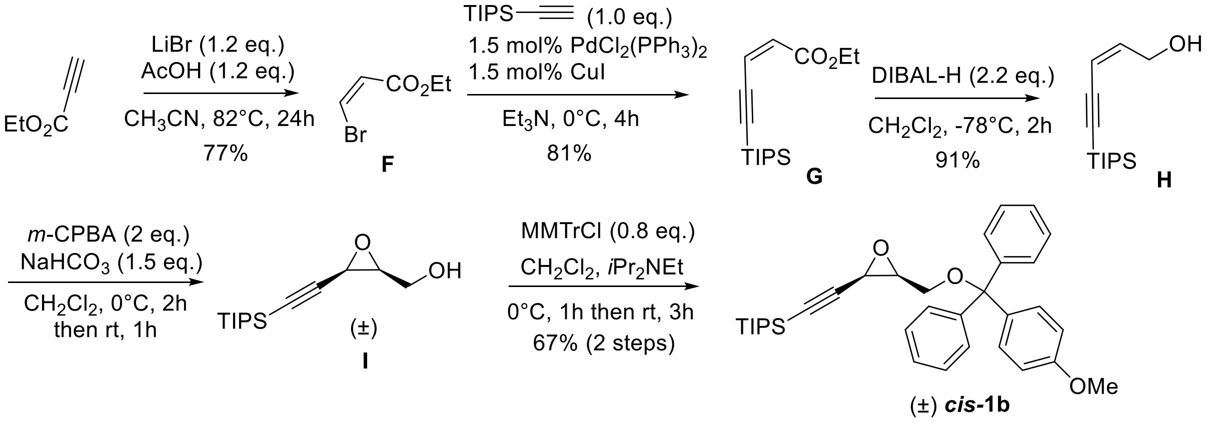

Ethyl (*Z*)-3-bromoacrylate (F).[3] LiBr (3.3g, 38 mmol) was dried at 120 °C under vacuum for 1 h. At room temperature, CH_3CN (33 mL), ethyl propiolate (3g, 31 mmol) and glacial acetic acid (2.3 g, 38 mmol) were successively added. The mixture was heated under reflux for 24 h. After cooling to rt, water (5 mL) was added and the mixture was neutralized by slow addition of solid K_2CO_3. The aqueous phase was extracted with Et_2O (3 × 10 mL), the organic phases were combined and dried over Na_2SO_4. After filtration and concentration under vacuum, 4.3 g of an orange liquid was obtained (77% yield). The product was used in the next step without further purification. ^{1}H NMR (500 MHz, $CDCl_3$) δ 6.97 (d, *J* = 8.4 Hz, 1H), 6.60 (d, *J* = 8.4 Hz, 1H), 4.23 (q, *J* = 7.1 Hz, 2H), 1.30 (t, *J* = 7.1 Hz, 3H).

Ethyl (*Z*)-5-(triisopropylsilyl)pent-2-en-4-ynoate (G). In a round-bottomed flask, at 0 °C, were successively added Et_3N (45 mL), **F** (5.25 g, 29 mmol), triisopropylsilylacetylene (6.5 mL, 29 mmol), $PdCl_2(PPh_3)_2$ (330 mg, 0.5 mmol) and CuI (95 mg, 0.5 mmol). The mixture was stirred at 0 °C for 4 h, then water (50 mL) was added. The crude was extracted with EtOAc (2 × 50 mL), washed with brine, dried over Na_2SO_4, filtered and concentrated in vacuum. After purification by chromatography on silica gel (cyclohexane/ethyl acetate 98:2), 6.6 g of an orange liquid were obtained (81% yield). ^{1}H NMR (300 MHz, $CDCl_3$) δ 6.14 (d, *J* = 11.7 Hz, 1H), 6.05 (d, *J* = 11.7 Hz, 1H), 4.21 (q, *J* = 7.1 Hz, 2H), 1.27 (t, *J* = 7.1 Hz, 3H), 0.80-1.50 (m, 21H). ^{13}C NMR (126 MHz, $CDCl_3$) δ 164.6, 129.4, 122.4, 105.1, 102.6, 60.5, 18.7, 14.4, 11.5. HRMS (ESI-TOF) *m*/*z* $[M + Na]^+$ Calcd for $C_{16}H_{28}NaO_2Si$ 303.1751; Found 303.1764.

(*Z*)-5-(Triisopropylsilyl)pent-2-en-4-yn-1-ol (H). To a solution of **G** (3.0 g, 10.6 mmol) in DCM (60 mL) at −78 °C was slowly added DIBAL-H (1.2 M in toluene, 20 mL, 24 mmol). The mixture was stirred 2 h at −78 °C and for 1 h at 0 °C. Then, NaOH (aq. 15%, 20 mL) was added at 0 °C and the two phases were separated. The aqueous phase was extracted with DCM (50 mL) and the organic phases were combined, washed with brine (50 mL) and dried over Na_2SO_4. After filtration and concentration, 2.54 g of a colorless oil was obtained (91% yield) which was used in the next step without further purification. ^{1}H NMR (500 MHz, $CDCl_3$) δ 6.10 (dt, *J* = 11.1, 6.1 Hz, 1H), 5.62 (d, *J* = 11.0 Hz, 1H), 4.43 (dt, *J* = 6.1, 1.4 Hz, 2H), 2.34 (s, 1H), 1.11-1.02 (m, 21H). ^{13}C NMR (126 MHz, $CDCl_3$) δ 142.6, 111.0, 102.4, 97.7, 61.4, 18.8, 11.3.

***cis*-(3-((Triisopropylsilyl)ethynyl)oxiran-2-yl)methanol (I).** The same protocol was used as for the synthesis of **E**, starting from 10.7 mmol of **H**. Compound **I** was used in the next step without further purification. ^{1}H NMR (500 MHz, $CDCl_3$) δ 4.01-3.81 (m, 2H), 3.54 (d, *J* = 4.1 Hz, 1H), 3.27 (dt, *J* = 6.3, 4.1 Hz, 1H), 1.05 (s, 21H). ^{13}C NMR (126 MHz, $CDCl_3$) δ 101.1, 88.5, 62.5, 57.5, 44.5, 18.7, 11.2.

***cis*-Triisopropyl((3-(((4-methoxyphenyl)diphenylmethoxy)methyl)oxiran-2-yl)ethynyl)silane (*cis*-1b).** The same protocol was used as for the synthesis of ***trans*-1b**, starting from 1.6 mmol of **I**. Yield = 67% (2 steps). ^{1}H NMR (500 MHz, $CDCl_3$) δ 7.48-7.41 (m, 4H), 7.32 (d, *J* = 9.0 Hz, 2H), 7.29-7.25 (m, 4H), 7.22-7.16 (m, 2H), 6.80 (d, *J* = 9.0, 2H), 3.77 (s, 3H), 3.34-3.45 (m, 3H), 3.24 (dt, *J* = 6.6, 3.8 Hz, 1H), 1.15-0.95 (m, 21H). ^{13}C NMR (126 MHz,

[3] Ma, S.; Lu, X. *Org. Synth.* **1995**, *72*, 112-15.

CDCl3) δ 158.7, 144.5, 144.3, 135.6, 130.5, 128.55, 128.50, 128.00, 127.95, 127.05, 127.00, 113.3, 101.4, 87.5, 86.9, 64.2, 56.9, 55.3, 43.8, 18.6, 11.1. HRMS (ESI-TOF) *m/z* $[M + K]^+$ Calcd for $C_{34}H_{42}KO_3Si$ 565.2535; Found 565.2557.

Synthesis of substrate *trans*-1d

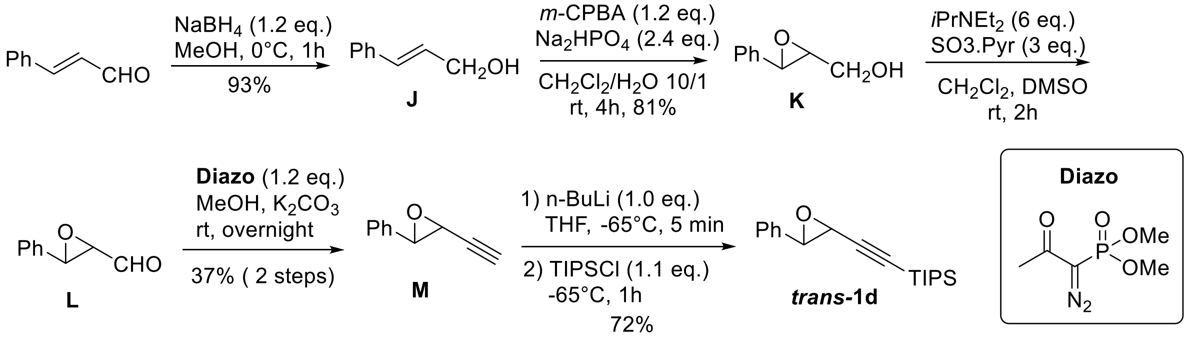

trans-2-Ethynyl-3-phenyloxirane (M)[4]

a) (*E*)-Cinnamaldehyde (5.28 g, 40 mmol) was dissolved in MeOH (100 mL) under nitrogen and the solution cooled to −5 °C. $NaBH_4$ (1.81 g, 48 mmol) was added protionwise while keeping the temperature below 0 °C. After the end of the addition, the mixture was stirred at 0 °C for 1 h. Water (20 mL) was slowly added and the temperature allowed to reach ambient temperature. Methanol was evaporated under vacuum and the residue was extracted with EtOAc (2 × 50 mL). The organic phases were combined and washed with brine (30 mL). After drying over Na_2SO_4, the solution was filtered and concentrated in vacuum to give pure alcohol **J** as colorless oil (5 g, 93% yield). ^1H NMR (300 MHz, $CDCl_3$) δ 7.40-7.20 (m, 5H), 6.63 (dt, *J* = 15.9, 1.5 Hz, 1H), 6.37 (dt, *J* = 15.9, 5.7 Hz, 1H), 3.33 (dd, *J* = 5.7, 1.5 Hz, 2H).

b) Compound **J** (4.98g, 37 mmol) was dissolved in DCM (185 mL) at room temperature. Water (18.5 mL), solid Na_2HPO_4 (12.6 g, 88.8 mmol) and mCPBA (77%, 10 g, 44.4 mmol) were successively added and the mixture was vigorously stirred for 4 h. A saturated solution of Na_2SO_3 (50 mL) was added and the phases was separated. The organic phase was washed with $NaHCO_3$ (2 × 50 mL), dried over Na_2SO_4, filtered and concentrated in vacuum to give pure epoxide **K** as yellow oil (4.5 g, 81% yield). ^1H NMR (300 MHz, $CDCl_3$) δ 7.32-7.18 (m, 5H), 3.98 (dd, *J* = 12.9, 2.4 Hz, 1H), 3.86 (d, *J* = 2.4 Hz, 1H), 3.73 (dd, *J* = 12.9, 2.4 Hz), 3.16 (d, *J* = 3.9, 2.4 Hz, 1H).

c) Compound **K** (3.75 g, 25 mmol) was dissolved in dry DCM (250 mL) under nitrogen, Hünigs base (14.3 mL, 150 mmol) was added and the solution was cooled to −5 °C. A solution of SO_3·pyridine (11.93 g, 75 mmol) in dry DMSO (45 mL) was slowly added. The cooling bath was removed and stirring was continued for 2 h. A saturated solution of NH_4Cl (50 mL) was added and the phases was separated. The organic phase was washed with sat. $NaHSO_4$ (50 mL) then with sat. $NaHCO_3$ (2 × 50 mL) and dried over Na_2SO_4. After filtration and concentration, the crude mixture containing **L** (peak of aldehyde was observed in ^1H NMR at 9.08 ppm in $CDCl_3$) was directly used in the next step.

4 Mitzel, T. M.; Palomo, C.; Jendza, K. *J. Org. Chem.* **2002**, *67*, 136-145.

d) Crude **L** was dissolved in MeOH (120 mL) and the solution was cooled to −5 °C. A solution of diazo compound (5.76 g, 30 mmol) in MeOH (30 mL) was added, followed by solid K_2CO_3 (6.9 g, 50 mmol). The cooling bath was removed and stirring was continued overnight. Methanol was evaporated under vacuum and the residue was extracted with EtOAc (2 × 100 mL). The organic phases were combined and washed with brine (50 mL). After drying over Na_2SO_4, the solution was filtered and concentrated. The crude product was purified by chromatography on silica gel (cyclohexane/EtOAc 95:5) to give compound **M** as colorless oil (1.33 g, 37% yield over two steps). ^{1}H NMR (500 MHz, $CDCl_3$) δ 7.40-7.32 (m, 3H), 7.30-7.25 (m, 2H), 4.06 (d, *J* = 1.8 Hz, 1H), 3.35 (t, *J* = 1.8 Hz, 1H), 2.41 (d, *J* = 1.8 Hz, 1H). ^{13}C NMR (126 MHz, $CDCl_3$) δ 135.5, 129.0, 128.8, 125.7, 80.0, 72.4, 59.9, 49.0.

***trans*-Triisopropyl((3-phenyloxiran-2-yl)ethynyl)silane (*trans*-1d).** Compound **M** (770 mg, 5.35 mmol) was dissolved in THF (50 mL) under argon and the solution was cooled to −65 °C. *n*-BuLi (1.6 M in hexanes, 3.35 mL, 5.35 mmol) was added and stirring was continued at −65 °C for 5 min. TIPS-Cl (1.13 g, 5.9 mmol) was added and stirring was maintained for 1 h at the same temperature. A saturated solution of NH_4Cl (10 mL) was added and the mixture was extracted with EtOAc (3 × 20 mL). The organic phases were combined and washed with brine (20 mL). After drying over Na_2SO_4, the solution was filtered and concentrated. The crude product was purified by chromatography on silica gel (cyclohexane/EtOAc 98:2) to give compound *trans*-**1d** as colorless oil (1.16 g, 72% yield). ^{1}H NMR (500 MHz, $CDCl_3$) δ 7.40-7.31 (m, 3H), 7.30-7.27 (m, 2H), 4.00 (d, *J* = 2.0 Hz, 1H), 3.38 (d, *J* = 2.0 Hz, 1H), 1.09 (s, 21H). ^{13}C NMR (126 MHz, $CDCl_3$) δ 135.9, 128.8, 128.7, 125.7, 103.2, 86.3, 60.5, 49.9, 18.7, 11.2.

Synthesis of substrate *cis*-1d

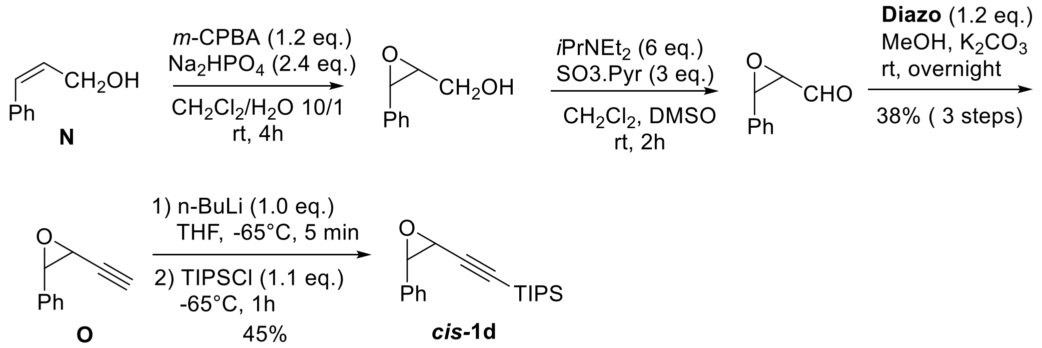

***cis*-2-Ethynyl-3-phenyloxirane (O).** It was obtained from allylalcohol **N**[5] following the same procedure used for the synthesis of **M** (38% yield over 3 steps). ^{1}H NMR (500 MHz, $CDCl_3$) δ 7.45-7.32 (m, 5H), 4.12 (d, *J* = 4.0 Hz, 1H), 3.76 (dd, *J* = 4.0, 1.5 Hz, 1H), 2.33 (d, *J* = 1.5 Hz, 1H). ^{13}C NMR (126 MHz, $CDCl_3$) δ 133.9, 128.7, 128.1, 127.0, 78.12, 74.8, 58.7, 48.0.

***cis*-Triisopropyl((3-phenyloxiran-2-yl)ethynyl)silane (*cis*-1d).** It was obtained from compound **O** following the same procedure used for the synthesis of *trans*-**1d** (45% yield). ^{1}H NMR (300 MHz, $CDCl_3$) δ 7.44-7.38 (m, 2H), 7.36-7.28 (m, 3H), 4.12 (d, *J* = 4.2 Hz, 1H), 3.77

[5] Kim, I. S.; Dong, G. R.; Jung, Y. H. *J. Org. Chem.* **2007**, *72*, 5424-5426.

(d, J = 4.2 Hz, 1H), 0.94 (s, 21H). ^{13}C NMR (126 MHz, $CDCl_3$) δ 134.3, 128.3, 127.9, 127.0, 101.2, 88.6, 59.2, 48.7, 18.5, 11.1.

Synthesis of substrate *cis*-1c

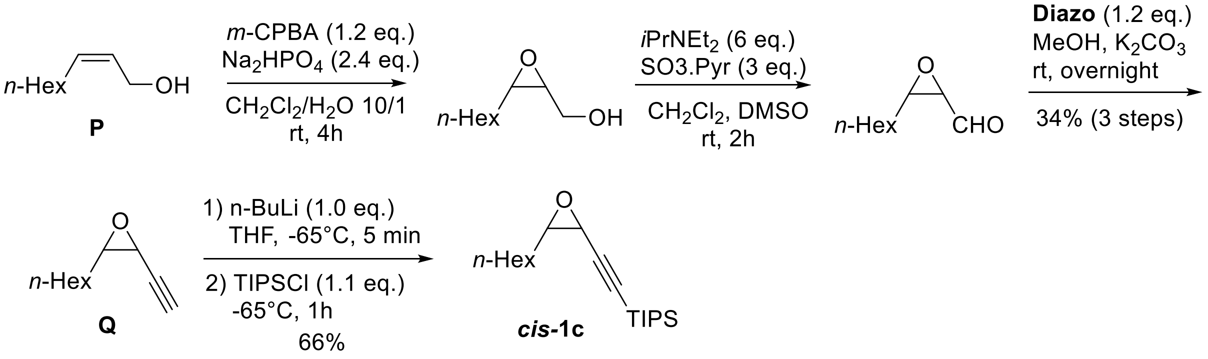

***cis*-2-Ethynyl-3-hexyloxirane (Q).**[6] It was obtained from allylalcohol **P**[7] following the same procedure used for the synthesis of **M** (34% yield over 3 steps). Cis/trans ratio: 9/1. ^{1}H NMR (500 MHz, $CDCl_3$) δ 3.41 (dd, J = 2.4, 1.2 Hz, 1H), 3.03 (td, J = 5.1, 2.4 Hz, 1H), 2.34 (d, J = 1.2 Hz, 1H), 1.69 (m, 2H), 1.49 (m, 2H), 1.40-1.20 (m, 6H), 0.88 (t, J = 4.2 Hz, 3H). ^{13}C NMR (126 MHz, $CDCl_3$) δ 79.1, 73.7, 58.1, 44.9, 31.9, 29.4, 29.2, 26.0, 22.7, 14.2.

***cis*-Triisopropyl((3-hexyloxiran-2-yl)ethynyl)silane (*cis*-1c).** It was obtained from compound **Q** following the same procedure used for the synthesis of *trans*-**1d** (66% yield). Cis/trans ratio: 9/1. ^{1}H NMR (500 MHz, $CDCl_3$) δ 3.44 (d, J = 4.0 Hz, 1H), 3.02 (dt, J = 6.0, 4.0 Hz, 1H), 1.83-1.75 (m, 1H), 1.69-1.61 (m, 1H), 1.52-1.44 (m, 2H), 1.40-1.33 (m, 2H), 1.32-1.24 (m, 4H), 1.07 (s, 21H), 0.88 (t, J = 6.5 Hz, 3H). ^{13}C NMR (126 MHz, $CDCl_3$) δ 102.4, 87.3, 58.4, 45.5, 31.9, 29.7, 29.4, 25.9, 22.7, 18.7, 14.2, 11.3.

Synthesis of substrate 1c-*trans*

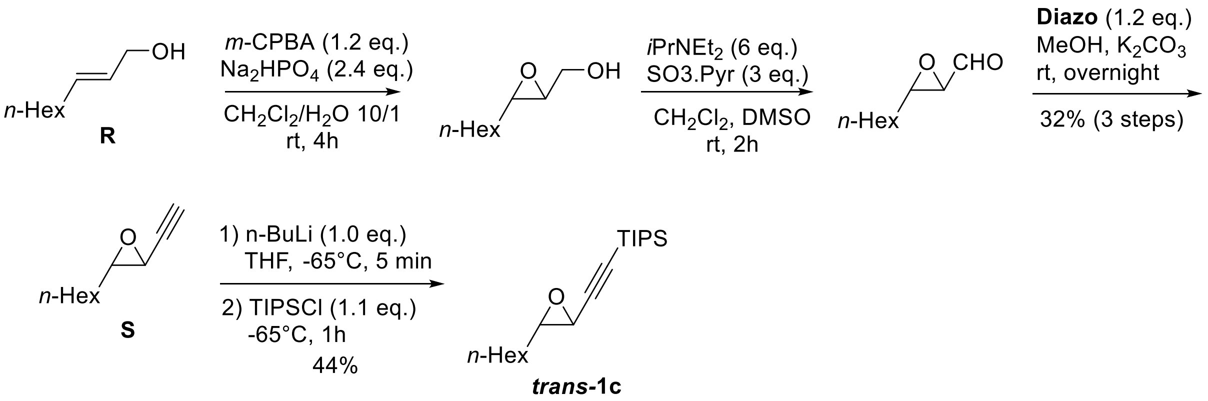

[6] Alexakis, A.; Marek, I.; Mangeney, P.; Normant, J. F. *Tetrahedron* **1991**, *47*, 1677-1696.
[7] Abascal, N. C.; Lichtor, P. A.; Giuliano, M. W.; Miller, S. J. *Chem. Sci.* **2014**, *5*, 4505-4511.

***trans*-2-Ethynyl-3-hexyloxirane (S).**[6] It was obtained from allylalcohol **R**[8] following the same procedure used for the synthesis of **M** (32% yield over 3 steps). Cis/trans ratio: 9/91. ^{1}H NMR (500 MHz, $CDCl_3$) δ 3.11-3.07 (m, 2H), 2.30 (d, *J* = 1.2 Hz, 1H), 1.55 (m, 2H), 1.39-1.23 (m, 8H), 0.88 (t, *J* = 4.2 Hz, 3H). ^{13}C NMR (126 MHz, $CDCl_3$) δ 80.8, 71.8, 60.6, 45.0, 31.8, 29.1, 27.1, 25.7, 22.7, 14.2.

***trans*-Triisopropyl((3-hexyloxiran-2-yl)ethynyl)silane (*trans*-1c).** It was obtained from compound **S** following the same procedure used for the synthesis of ***trans*-1d** (44% yield). Cis/trans > 5/95. ^{1}H NMR (500 MHz, $CDCl_3$) δ 3.11 (d, J = 2.5 Hz, 1H), 3.07 (dt, J = 4.5, 2.5 Hz, 1H), 1.65-1.57 (m, 1H), 1.52-1.41 (m, 3H), 1.37-1.25 (m, 6H), 1.06 (m, 21H), 0.88 (t, J = 6.5 Hz, 3H). ^{13}C NMR (126 MHz, $CDCl_3$) δ 104.2, 85.4, 61.1, 45.7, 31.9, 29.1, 25.8, 22.7, 18.7, 14.2, 11.2.

Synthesis of substrate *cis*-1e

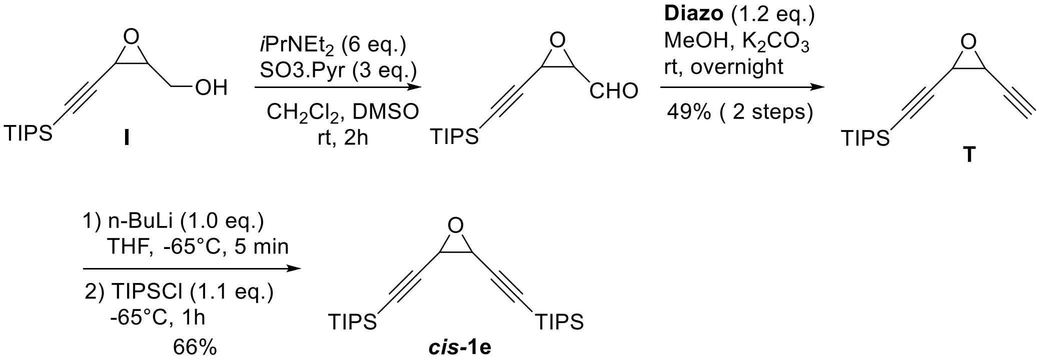

***cis*-((3-Ethynyloxiran-2-yl)ethynyl)triisopropylsilane (T).** It was obtained from compound **I** following the same procedure used for the synthesis of **M** (49% yield over 2 steps: oxidation and alkynylation). ^{1}H NMR (300 MHz, $CDCl_3$) δ 3.57 (d, *J* = 3.9 Hz, 1H), 3.53 (dd, *J* = 3.9, 1.5 Hz, 1H), 2.39 (d, *J* = 1.5 Hz, 1H), 1.09 (s, 21H). ^{13}C NMR (126 MHz, $CDCl_3$) δ 105.5, 88.6, 78.1, 74.3, 46.6, 46.4, 18.6, 11.2. HRMS (ESI-TOF) *m/z* $[M + H]^+$ Calcd for $C_{15}H_{25}OSi$ 249.1669; Found 249.1677.

***cis*-2,3-Bis((triisopropylsilyl)ethynyl)oxirane (*cis*-1e).** It was obtained from compound **T** following the same procedure used for the synthesis of *trans*-**1d** (66% yield). ^{1}H NMR (500 MHz, $CDCl_3$) δ 3.59 (s, 2H), 1.08 (s, 42H). ^{13}C NMR (126 MHz, $CDCl_3$) δ 100.7, 89.4, 47.1, 18.7, 11.2. HRMS (ESI-TOF) *m/z* $[M + Na]^+$ Calcd for $C_{24}H_{44}NaOSi_2$ 427.2823; Found 427.2822.

[8] Fernández-Mateos, A.; Encinas Madrazo, S.; Herrero Teijón, P.; Rubio González, R. *Eur. J. Org. Chem.* **2010**, 856-861.

Synthesis of substrate 1e-*trans*

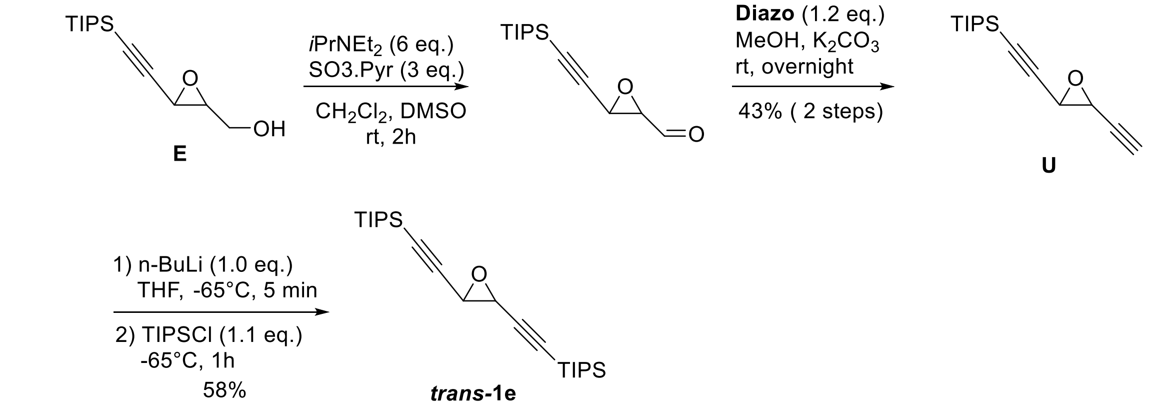

***trans*-((3-Ethynyloxiran-2-yl)ethynyl)triisopropylsilane (U).** It was obtained from compound **E** following the same procedure used for the synthesis of **M** (43% yield over 2 steps: oxidation and alkynylation). ^{1}H NMR (300 MHz, $CDCl_3$) δ 3.54 (d, *J* = 2.1 Hz, 1H), 3.49 (dd, *J* = 2.1, 1.5 Hz, 1H), 2.35 (d, *J* = 1.5 Hz, 1H), 1.06 (s, 21H). ^{13}C NMR (126 MHz, $CDCl_3$) δ 101.6, 87.3, 79.0, 72.8, 47.24, 47.18, 18.6, 11.2. HRMS (ESI-TOF) *m/z* $[M + H]^+$ Calcd for $C_{15}H_{25}OSi$ 249.1669; Found 249.1660.

***trans*-2,3-Bis((triisopropylsilyl)ethynyl)oxirane (*trans*-1e).** It was obtained from compound **U** following the same procedure used for the synthesis of *trans*-**1d** (58% yield). ^{1}H NMR (300 MHz, $CDCl_3$) δ 3.50 (s, 2H), 1.06 (s, 42H). ^{13}C NMR (126 MHz, $CDCl_3$) δ 102.1, 86.9, 47.8, 18.6, 11.2. HRMS (ESI-TOF) *m/z* $[M + H]^+$ Calcd for $C_{24}H_{45}OSi_2$ 405.3003; Found 405.2997.

Synthesis of substrate *trans*-1f

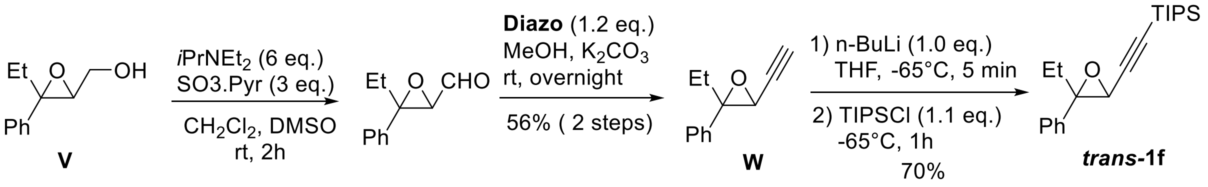

***trans*-2-Ethyl-3-ethynyl-2-phenyloxirane W.** It was obtained from compound **V** following the same procedure used for the synthesis of **M** (56% yield over 2 steps). ^{1}H NMR (300 MHz, $CDCl_3$) δ 7.40-7.27 (m, 5H), 3.34 (d, *J* = 2.0 Hz, 1H), 2.48 (d, *J* = 2.0 Hz, 1H), 2.31 (sext, *J* = 7.5 Hz, 1H), 2.00 (sext, *J* = 7.5 Hz, 1H), 0.98 (t, *J* = 7.5 Hz, 3H). ^{13}C NMR (126 MHz, $CDCl_3$) δ 138.5, 128.5, 128.0, 126.2, 79.2, 74.4, 66.7, 53.4, 26.1, 9.3.

***trans*-((3-Ethyl-3-phenyloxiran-2-yl)ethynyl)triisopropylsilane (*trans*-1f).** It was obtained from compound **W** following the same procedure used for the synthesis of *trans*-**1d** (70% yield). ^{1}H NMR (300 MHz, $CDCl_3$) δ 7.45-7.26 (m, 5H), 3.38 (s, 1H), 2.29 (sext, *J* = 7.5 Hz, 1H), 2.05 (sext, *J* = 7.5 Hz, 1H), 1.10 (s, 21H), 0.99 (t, *J* = 7.5 Hz, 3H). ^{13}C NMR (126 MHz, $CDCl_3$) δ 138.9, 128.5, 127.9, 126.3, 102.4, 88.2, 67.0, 54.0, 26.3, 18.7, 11.3, 9.5. HRMS (ESI-TOF) *m/z* $[M + H]^+$ Calcd for $C_{21}H_{33}OSi$ 329.2295; Found 329.2289.

Synthesis of substrate cis-1f

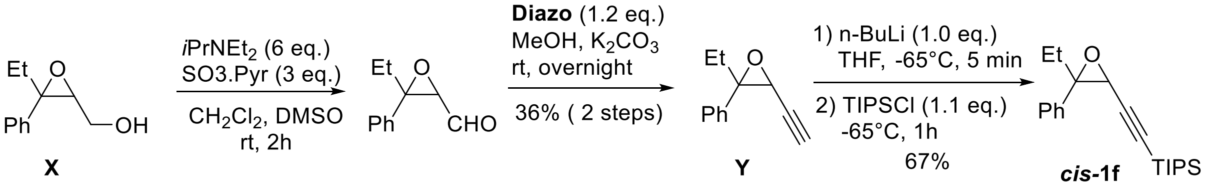

***cis*-2-Ethyl-3-ethynyl-2-phenyloxirane (Y).** It was obtained from compound **X** following the same procedure used for the synthesis of **M** (36% yield over 2 steps). ^{1}H NMR (300 MHz, $CDCl_3$) δ 7.45-7.28 (m, 5H), 3.58 (d, *J* = 1.8 Hz, 1H), 2.25 (sext, *J* = 7.8 Hz, 1H), 2.10 (d, *J* = 1.8 Hz, 1H), 1.72 (sext, *J* = 7.8 Hz, 1H), 0.93 (t, *J* = 7.8 Hz, 3H). ^{13}C NMR (126 MHz, $CDCl_3$) δ 137.0, 128.0, 127.8, 127.0, 79.1, 74.3, 67.8, 51.9, 29.5, 8.9.

***cis*-((3-Ethyl-3-phenyloxiran-2-yl)ethynyl)triisopropylsilane (*cis*-1f).** It was obtained from compound **Y** following the same procedure used for the synthesis of *trans*-**1d** (67% yield). ^{1}H NMR (300 MHz, $CDCl_3$) δ 7.38 (d, *J* = 7.5 Hz, 2H), 7.30 (t, *J* = 7.5 Hz, 2H), 7.24 (t, *J* = 7.5 Hz, 1H), 3.57 (s, 1H), 2.26 (sext, *J* = 7.5 Hz, 1H), 1.70 (sext, *J* = 7.5 Hz, 1H), 0.91 (t, *J* = 7.5 Hz, 3H), 0.84 (s, 21H). ^{13}C NMR (126 MHz, $CDCl_3$) δ 137.4, 128.0, 127.6, 127.1, 102.4, 87.8, 68.2, 52.6, 29.5, 18.5, 11.0, 9.0. HRMS (ESI-TOF) *m*/*z* $[M + H]^+$ Calcd for $C_{21}H_{33}OSi$ 329.2295; Found 329.2290.

General procedure for the synthesis of enynes

A solution of ethynyloxirane **1** (0.38–0.5 mmol) in THF (0.125 M) was cooled to −92 °C (for *trans*-**1**) or −78 °C (for *cis*-**1**). A solution of *n*-BuLi (1.4 M in hexanes, 1.5 equiv) was slowly added and stirring was continued at the same temperature (60 min for *trans*-**1** or 30 min for *cis*-**1**). A solution of boronic ester (1.5 equiv) in THF (0.25 M) was slowly added and stirring was continued at the same temperature (90 min for *trans*-**1** or 20 min for *cis*-**1**). The mixture was slowly raised to rt and stirred for 1 h. A saturated solution of NH_4Cl (5 mL) was added and the mixture was extracted with EtOAc (2 × 15 mL). The organic phases were combined, washed with brine (20 mL) and dried over Na_2SO_4. After filtration and concentration under vacuum, the crude was purified by column chromatography on silica gel (cyclohexane/EtOAc 98:2).

Triisopropyl(4-methyl-3-phenylpent-3-en-1-yn-1-yl)silane (3). ^{1}H NMR (500 MHz, $CDCl_3$) δ 7.36-7.29 (m, 4H), 7.25-7.21 (m, 1H), 2.17 (s, 3H), 1.84 (s, 3H), 1.08 (s, 21H). ^{13}C NMR (126 MHz, $CDCl_3$) δ 144.0, 139.4, 129.3, 128.0, 126.7, 119.7, 107.8, 93.1, 24.5, 21.8, 18.9, 11.5. HRMS (ESI-TOF) *m/z* $[M + H]^+$ Calcd for $C_{21}H_{33}Si$ 313.2346; Found 313.2348.

(*Z*)-Triisopropyl(5-((4-methoxyphenyl)diphenylmethoxy)-3-phenylpent-3-en-1-yn-1-yl)silane ((*Z*)-4). ^{1}H NMR (500 MHz, $CDCl_3$) δ 7.61 (d, *J* = 7.5 Hz, 2H), 7.49 (d, *J* = 7.0 Hz, 4H), 7.40-7.20 (m, 11H), 6.83 (d, *J* = 9.0 Hz, 2H), 6.62 (t, *J* = 5.8 Hz, 1H), 4.19 (d, *J* = 5.8 Hz, 2H), 3.78 (s, 3H), 1.00 (m, 21H). ^{13}C NMR (126 MHz, $CDCl_3$) δ 158.6, 144.7, 137.2, 136.1, 135.8, 130.5, 128.5, 128.4, 128.0, 127.9, 127.0, 126.1, 123.4, 113.3, 102.9, 98.7, 86.9, 64.6, 55.3, 18.8, 11.3. HRMS (ESI-TOF) *m/z* $[M + K]^+$ Calcd for $C_{40}H_{46}KO_2Si$ 625.2899; Found 625.2889.

(*E*)-Triisopropyl(5-((4-methoxyphenyl)diphenylmethoxy)-3-phenylpent-3-en-1-yn-1-yl)silane ((*E*)-4). ^{1}H NMR (300 MHz, $CDCl_3$) δ 7.47-7.40 (m, 5H), 7.35-7.15 (m, 12H), 6.80 (d, *J* = 9.0 Hz, 2H), 6.37 (t, *J* = 6.9 Hz, 1H), 3.81 (d, *J* = 6.9 Hz, 2H), 3.78 (s, 3H), 1.10 (s, 21H). ^{13}C NMR (126 MHz, $CDCl_3$) δ 158.6, 144.5, 136.7, 135.9, 135.8, 130.4, 128.6, 128.5, 127.9, 127.8, 127.0, 126.2, 113.2, 108.2, 89.9, 86.9, 61.6, 55.3, 18.7, 11.5. HRMS (ESI-TOF) *m/z* $[M + K]^+$ Calcd for $C_{40}H_{46}KO_2Si$ 625.2899; Found 625.2925.

(*Z*)-Triisopropyl(3-(4-methoxyphenyl)-5-((4-methoxyphenyl)diphenylmethoxy)pent-3-en-1-yn-1-yl)silane ((*Z*)-5). ^{1}H NMR (500 MHz, $CDCl_3$) δ 7.55 (d, *J* = 9.0 Hz, 2H), 7.48 (d, *J* = 7.5 Hz, 4H), 7.35 (d, *J* = 8.5 Hz, 2H), 7.29 (t, *J* = 7.0 Hz, 4H), 7.21 (t, *J* = 7.5 Hz, 2H), 6.87 (d, *J* = 9.0 Hz, 2H), 6.82 (d, *J* = 9.0 Hz, 2H), 6.49 (t, *J* = 6.0 Hz, 1H), 4.16 (d, *J* = 6.0 Hz, 2H), 3.82 (s, 3H), 3.78 (s, 3H), 0.99 (s, 21H). ^{13}C NMR (126 MHz, $CDCl_3$) δ 159.5, 158.6, 144.7, 135.9, 134.0, 130.5, 129.9, 128.5, 128.0, 127.3, 126.9, 122.9, 113.8, 113.3, 103.1, 98.3, 86.9, 64.6, 55.5, 55.3, 18.8, 11.3. HRMS (ESI-TOF) *m/z* $[M + K]^+$ Calcd for $C_{41}H_{48}KO_3Si$ 655.3004; Found 655.3012.

(*E*)-Triisopropyl(3-(4-methoxyphenyl)-5-((4-methoxyphenyl)diphenylmethoxy)pent-3-en-1-yn-1-yl)silane ((*E*)-5). ^{1}H NMR (500 MHz, $CDCl_3$) δ 7.47-7.44 (m, 4H), 7.33 (d, *J* = 8.5 Hz, 2H), 7.28 (t, *J* = 7.5 Hz, 4H), 7.24-7.18 (m, 4H), 6.81 (d, *J* = 9.0 Hz, 2H), 6.73 (d, *J* = 9.0 Hz, 2H), 6.32 (t, *J* = 7.0 Hz, 1H), 3.82 (d, *J* = 7.0 Hz, 2H), 3.79 (s, 3H), 3.78 (s, 3H), 1.11 (s, 21H). ^{13}C NMR (126 MHz, $CDCl_3$) δ 159.1, 158.6, 144.6, 135.8, 134.7, 130.4, 129.9, 129.2,

128.5, 128.0, 127.0, 125.8, 113.3, 113.2, 108.5, 89.6, 86.8, 61.7, 55.3, 18.8, 11.5. HRMS (ESI-TOF) *m*/*z* [M + Na]$^+$ Calcd for $C_{41}H_{48}NaO_3Si$ 639.3265; Found 639.3225.

(*Z*)-(3-(4-Chlorophenyl)-5-((4-methoxyphenyl)diphenylmethoxy)pent-3-en-1-yn-1-yl)triisopropylsilane ((*Z*)-6). ^{1}H NMR (500 MHz, $CDCl_3$) δ 7.52 (d, *J* = 8.5 Hz, 2H), 7.47 (d, *J* = 7.0 Hz, 4H), 7.35 (d, *J* = 9.0 Hz, 2H), 7.32-7.26 (m, 6H), 7.21 (t, *J* = 7.0 Hz, 2H), 6.82 (d, *J* = 9.0 Hz, 2H), 6.55 (t, *J* = 5.5 Hz, 1H), 4.18 (d, *J* = 5.5 Hz, 2H), 3.77 (s, 3H), 0.98 (s, 21H). ^{13}C NMR (126 MHz, $CDCl_3$) δ 158.7, 144.6, 136.5, 135.8, 133.7, 130.5, 128.6, 128.5, 128.0, 127.4, 127.0, 122.4, 113.3, 102.4, 99.2, 87.0, 64.6, 55.3, 18.8, 11.3. HRMS (ESI-TOF) *m*/*z* [M + K]$^+$ Calcd for $C_{40}H_{45}KClO_2Si$ 659.2509; Found 659.2493.

(*E*)-(3-(4-Chlorophenyl)-5-((4-methoxyphenyl)diphenylmethoxy)pent-3-en-1-yn-1-yl)triisopropylsilane ((*E*)-6). ^{1}H NMR (500 MHz, $CDCl_3$) δ 7.50-7.55 (m, 4H), 7.34-7.41 (m, 9H), 7.28-7.33 (m, 3H), 6.88-6.91 (m, 2H), 6.44 (t, *J* = 7.0 Hz, 1H), 3.89 (s, 3H), 3.85 (d, *J* = 7.0 Hz, 2H), 1.14-1.21 (m, 21H). ^{13}C NMR (126 MHz, $CDCl_3$) δ 158.7, 144.4, 136.2, 135.2, 135.6, 133.6, 130.4, 130.0, 128.4, 128.2, 128.0, 127.1, 125.4, 113.2, 107.7, 90.6, 86.9, 61.3, 55.4, 18.8, 11.4. HRMS (ESI-TOF) *m*/*z* [M + K]$^+$ Calcd for $C_{40}H_{45}KClO_2Si$ 659.2509; Found 659.2569.

(*Z*)-(3-Benzyl-5-((4-methoxyphenyl)diphenylmethoxy)pent-3-en-1-yn-1-yl)triisopropylsilane ((*Z*)-7). ^{1}H NMR (500 MHz, $CDCl_3$) δ 7.44 (d, *J* = 7.5 Hz, 4H), 7.31 (d, J = 8.5 Hz, 2H), 7.30-7.15 (m, 11H), 6.80 (d, *J* = 8.5 Hz, 2H), 6.00 (t, *J* = 6.0 Hz, 1H), 3.94 (d, *J* = 6.0 Hz, 2H), 3.78 (s, 3H), 3.43 (s, 2H), 0.87 (s, 21H). ^{13}C NMR (126 MHz, $CDCl_3$) δ 158.6, 144.8, 138.9, 136.05, 136.0, 130.4, 129.2, 128.5, 128.3, 127.9, 126.9, 126.4, 123.7, 113.2, 104.3, 97.2, 86.8, 64.0, 55.3, 43.5, 18.6, 11.2. HRMS (ESI-TOF) *m*/*z* [M + K]$^+$ Calcd for $C_{41}H_{48}KO_2Si$ 639.3055; Found 639.3067.

(*E*)-(3-Benzyl-5-((4-methoxyphenyl)diphenylmethoxy)pent-3-en-1-yn-1-yl)triisopropylsilane ((*E*)-7). ^{1}H NMR (500 MHz, $CDCl_3$) δ 7.46 (d, *J* = 7.5 Hz, 4H), 7.34 (d, *J* = 8.5 Hz, 2H), 7.29 (t, *J* = 7.5 Hz, 4H), 7.25-7.13 (m, 5H), 7.09 (d, J = 7.5 Hz, 2H), 6.83 (d, *J* = 8.5 Hz, 2H), 6.21 (t, *J* = 6.0 Hz, 1H), 3.79 (s, 3H), 3.78 (d, *J* = 6.0 Hz, 2H), 3.27 (s, 2H), 0.99 (s, 21H). ^{13}C NMR (126 MHz, $CDCl_3$) δ 158.7, 144.5, 138.8, 135.8, 135.1, 130.5, 128.9, 128.5, 128.3, 128.0, 127.0, 126.3, 124.7, 113.3, 108.6, 89.8, 86.8, 60.8, 55.4, 37.8, 18.7, 11.4. HRMS (ESI-TOF) *m*/*z* [M + K]$^+$ Calcd for $C_{41}H_{48}KO_2Si$ 639.3055; Found 639.3037.

(*Z*)-Triisopropyl(3-(2-((4-methoxyphenyl)diphenylmethoxy)ethylidene)hex-5-en-1-yn-1-yl)silane ((*Z*)-8). ^{1}H NMR (500 MHz, $CDCl_3$) δ 7.45 (d, *J* = 7.5 Hz, 4H), 7.33 (d, *J* = 9.0 Hz, 2H), 7.28 (t, *J* = 7.0 Hz, 4H), 7.21 (t, *J* = 7.0 Hz, 2H), 6.82 (d, *J* = 9.0 Hz, 2H), 5.95 (t, *J* = 6.0 Hz, 1H), 5.82 (ddt, *J* = 17.0, 10.0, 6.5 Hz, 1H), 5.10 (dd, *J* = 17.0, 1.5 Hz, 1H), 5.06 (dd, *J* = 10.0, 1.5 Hz, 1H), 3.95 (d, *J* = 6.0 Hz, 2H), 3.79 (s, 3H), 2.88 (d, *J* = 6.5 Hz, 2H), 1.00-0.90 (m, 21H). ^{13}C NMR (126 MHz, $CDCl_3$) δ 158.6, 144.8, 135.9, 135.6, 135.2, 130.4, 128.5, 127.9, 126.9, 122.6, 116.7, 113.2, 104.4, 96.6, 86.7, 63.9, 55.3, 41.4, 18.7, 11.2. HRMS (ESI-TOF) *m*/*z* [M + K]$^+$ Calcd for $C_{37}H_{46}KO_2Si$ 589.2899; Found 589.2931.

(*E*)-Triisopropyl(3-(2-((4-methoxyphenyl)diphenylmethoxy)ethylidene)hex-5-en-1-yn-1-yl)silane ((*E*)-8). ^{1}H NMR (500 MHz, $CDCl_3$) δ 7.45 (d, *J* = 7.5 Hz, 4H), 7.33 (d, *J* = 8.8 Hz, 2H), 7.29 (t, *J* = 7.5 Hz, 4H), 7.21 (t, *J* = 7.5 Hz, 2H), 6.83 (d, *J* = 8.9 Hz, 2H), 6.14 (t, *J* = 6.5 Hz, 1H), 5.71 (ddt, *J* = 17.0, 10.5, 6.5 Hz, 1H), 4.94 (dd, *J* = 17.0, 1.5 Hz, 1H), 4.93 (dd, *J* = 10.5, 1.5 Hz, 1H), 3.78 (s, 3H), 3.68 (d, *J* = 6.5 Hz, 2H), 2.69 (d, *J* = 6.5 Hz, 2H), 1.10-1.05 (m, 21H). ^{13}C NMR (126 MHz, $CDCl_3$) δ 158.7, 144.5, 135.8, 135.1, 134.7, 130.4, 128.5, 128.0, 127.0, 123.3, 116.2, 113.2, 108.5, 89.2, 86.7, 60.6, 55.4, 36.3, 18.8, 11.4. HRMS (ESI-TOF) *m*/*z* $[M + K]^+$ Calcd for $C_{37}H_{46}KO_2Si$ 589.2899; Found 589.2902.

(*Z*)-Triisopropyl(3-(2-((4-methoxyphenyl)diphenylmethoxy)ethylidene)hept-1-yn-1-yl)silane ((*Z*)-9). ^{1}H NMR (500 MHz, $CDCl_3$) δ 7.47 (d, *J* = 7.5 Hz, 4H), 7.35 (d, *J* = 9.0 Hz, 2H), 7.29 (t, *J* = 7.5 Hz, 4H), 7.22 (t, *J* = 7 Hz, 2H), 6.83 (d, *J* = 9.0 Hz, 2H), 5.95 (t, *J* = 6.0 Hz, 1H), 3.94 (d, *J* = 6.0 Hz, 1H), 3.80 (s, 3H), 2.15 (t, *J* = 7.0 Hz, 2H), 1.53 (quint, *J* = 7.0 Hz, 2H), 1.35 (quint, *J* = 7.0 Hz, 2H), 0.98 (s, 21H), 0.92 (t, *J* = 7.0 Hz, 3H). ^{13}C NMR (126 MHz, $CDCl_3$) δ 158.5, 144.8, 136.0, 134.9, 130.4, 128.5, 127.9, 126.9, 124.6, 113.2, 104.7, 96.1, 86.7, 63.9, 55.2, 36.7, 30.4, 22.1, 18.7, 14.1, 11.2. HRMS (ESI-TOF) *m*/*z* $[M + Na]^+$ Calcd for $C_{38}H_{50}NaO_2Si$ 589.3472; Found 589.3466.

(*E*)-Triisopropyl(3-(2-((4-methoxyphenyl)diphenylmethoxy)ethylidene)hept-1-yn-1-yl)silane ((*E*)-9). ^{1}H NMR (500 MHz, $CDCl_3$) δ 7.48 (d, *J* = 7.5 Hz, 4H), 7.36 (d, *J* = 9.0 Hz, 2H), 7.31 (t, *J* = 7.5 Hz, 4H), 7.24 (t, *J* = 7 Hz, 2H), 6.85 (d, *J* = 9.0 Hz, 2H), 6.13 (t, *J* = 6.0 Hz, 1H), 3.81 (s, 3H), 3.69 (d, *J* = 6.0 Hz, 1H), 1.95 (t, *J* = 7.0 Hz, 2H), 1.45 (quint, *J* = 7.0 Hz, 2H), 1.24 (quint, *J* = 7.0 Hz, 2H), 1.11 (s, 21H), 0.83 (t, *J* = 7.0 Hz, 3H). ^{13}C NMR (126 MHz, $CDCl_3$) δ 158.6, 144.6, 135.8, 134.5, 130.4, 128.5, 127.9, 127.0, 125.8, 113.2, 108.8, 88.7, 86.6, 60.6, 55.3, 31.0, 30.4, 22.1, 18.8, 14.0, 11.4. HRMS (ESI-TOF) *m*/*z* $[M + K]^+$ Calcd for $C_{38}H_{50}KO_2Si$ 605.3212; Found 605.3228.

(*Z*)-Triisopropyl(3-phenyldec-3-en-1-yn-1-yl)silane ((*Z*)-10). Z/E 9:1. ^{1}H NMR (500 MHz, $CDCl_3$) δ 7.62 (d, *J* = 7.5 Hz, 2H), 7.33 (t, *J* = 7.5 Hz, 2H), 7.24 (t, *J* = 7.5 Hz, 1H), 6.48 (t, *J* = 7.5 Hz, 1H), 2.53 (q, *J* = 7.5 Hz, 2H), 1.50 (quint, *J* = 7.5 Hz, 2H), 1.37 (quint, *J* = 7.5 Hz, 2H), 1.33-1.24 (m, 4H), 1.14 (s, 21H), 0.89 (t, *J* = 7.5 Hz, 3H). ^{13}C NMR (126 MHz, $CDCl_3$) δ 139.8, 138.2, 128.4, 127.4, 126.0, 123.9, 104.2, 96.6, 31.9, 31.7, 29.3, 29.2, 22.8, 18.9, 14.2, 11.5. HRMS (ESI-TOF) *m*/*z* $[M + H]^+$ Calcd for $C_{25}H_{41}Si$ 369.2972; Found 369.2950.

(*E*)-Triisopropyl(3-phenyldec-3-en-1-yn-1-yl)silane ((*E*)-10). ^{1}H NMR (500 MHz, $CDCl_3$) δ 7.39 (m, 2H), 7.34 (m, 2H), 7.26 (m, 1H), 6.24 (t, *J* = 8.0 Hz, 1H), 2.23 (q, *J* = 7.5 Hz, 2H), 1.42 (m, 2H), 1.33-1.17 (m, 6H), 1.08 (m, 21H), 0.86 (t, *J* = 7.5 Hz, 3H). ^{13}C NMR (126 MHz, $CDCl_3$) δ 141.2, 137.7, 128.9, 128.1, 127.3, 123.7, 109.3, 87.7, 31.8, 29.9, 29.7, 29.2, 22.7, 18.8, 14.2, 11.5. HRMS (ESI-TOF) *m*/*z* $[M + H]^+$ Calcd for $C_{25}H_{41}Si$ 369.2972; Found 369.2952.

(*Z*)-(3,4-Diphenylbut-3-en-1-yn-1-yl)triisopropylsilane ((*Z*)-11).[9] ^{1}H NMR (500 MHz, $CDCl_3$) δ 8.08 (d, *J* = 7.0 Hz, 2H), 7.77 (d, *J* = 7.0 Hz, 2H), 7.44-7.28 (m, 6H), 7.19 (s, 1H),

[9] Zhou, Y.; Ye, F.; Zhou, Q.; Zhang, Y.; Wang, J. *Org. Lett.* **2016**, *18*, 2024–2027.

1.26-1.12 (m, 21H). ^{13}C NMR (126 MHz, $CDCl_3$) δ 139.8, 136.6, 135.3, 129.4, 128.5, 128.3, 127.9, 126.6, 121.9, 105.6, 100.6, 18.9, 11.6.

(*E*)-(3,4-Diphenylbut-3-en-1-yn-1-yl)triisopropylsilane ((*E*)-11).[10] ^{1}H NMR (500 MHz, $CDCl_3$) δ 7.41-7.38 (m, 2H), 7.30-7.25 (m, 3H), 7.18-7.13 (m, 3H), 7.11-7.07 (m, 2H), 1.12 (s, 21H). ^{13}C NMR (126 MHz, $CDCl_3$) δ 137.6, 136.9, 136.2, 129.5, 129.3, 128.4, 128.2, 127.9, 127.7, 124.6, 109.8, 91.1, 18.9, 11.5.

(*Z*)-(3-Phenylhexa-3-en-1,5-diyne-1,6-diyl)bis(triisopropylsilane) ((*Z*)-12). ^{1}H NMR (500 MHz, $CDCl_3$) δ 7.65 (d, *J* = 7.0 Hz, 2H), 7.38-7.28 (m, 3H), 6.39 (s, 1H), 1.14-1.13 (m, 21H), 1.12 (s, 21H). ^{13}C NMR (126 MHz, $CDCl_3$) δ 137.4, 133.1, 128.8, 128.5, 126.3, 114.5, 105.2, 104.1, 101.2, 100.9, 18.92, 18.88, 11.5. HRMS (ESI-TOF) *m*/*z* $[M + H]^+$ Calcd for $C_{30}H_{49}Si_2$ 465.3367; Found 465.3362.

(*E*)-(3-Phenylhexa-3-en-1,5-diyne-1,6-diyl)bis(triisopropylsilane) ((*E*)-12). ^{1}H NMR (500 MHz, $CDCl_3$) δ 8.08 (dd, J = 8.0, 2.0 Hz, 2H), 7.38-7.27 (m, 3H), 6.27 (s, 1H), 1.13-1.09 (m, 21H), 1.08-1.04 (m, 21H). ^{13}C NMR (126 MHz, $CDCl_3$) δ 136.1, 133.4, 128.8, 128.6, 127.9, 115.3, 107.7, 104.8, 103.3, 95.4, 18.8, 18.7, 11.4. HRMS (ESI-TOF) *m*/*z* $[M + H]^+$ Calcd for $C_{30}H_{49}Si_2$ 465.3367; Found 465.3388.

(*Z*)-(3,4-Diphenylhex-3-en-1-yn-1-yl)triisopropylsilane ((*Z*)-13). ^{1}H NMR (500 MHz, $CDCl_3$) δ 7.46 (m, 4H), 7.41-7.24 (m, 6H), 2.51 (q, J = 7.5 Hz, 2H), 1.00-0.85 (m, 24H). ^{13}C NMR (126 MHz, $CDCl_3$) δ 152.5, 141.8, 139.8, 129.0, 128.8, 128.2, 128.0, 127.3, 127.2, 121.1, 108.3, 93.4, 27.6, 18.7, 13.4, 11.4. HRMS (ESI-TOF) *m*/*z* $[M + H]^+$ Calcd for $C_{27}H_{37}Si$ 389.2659; Found 389.2685.

(*E*)-(3,4-Diphenylhex-3-en-1-yn-1-yl)triisopropylsilane ((*E*)-13). ^{1}H NMR (500 MHz, $CDCl_3$) δ 7.21-7.12 (m, 5H), 7.10-7.06 (m, 3H), 7.05-7.02 (m, 2H), 2.95 (q, *J* = 7.5 Hz, 2H), 1.14 (s, 21H), 1.07 (t, *J* = 7.5 Hz, 3H). ^{13}C NMR (126 MHz, $CDCl_3$) δ 152.6, 140.9, 138.8, 130.0, 129.3, 128.1, 127.6, 127.0, 126.5, 120.5, 107.5, 95.1, 31.9, 18.9, 12.7, 11.6. HRMS (ESI-TOF) *m*/*z* $[M + H]^+$ Calcd for $C_{27}H_{37}Si$ 389.2659; Found 389.2663.

Deuteration experiments at −78 °C

A solution of ethynyloxirane **1b** (*cis* or *trans*, 0.2 mmol) in THF (1.6 mL) was cooled to −78 °C. A solution of *n*-BuLi (1.4 M in hexanes, 0.3 mmol, 0.21 mL) was slowly added and stirring was continued at the same temperature for 30 min. D_2O was slowly added and stirring was maintained at the same temperature for 20 min. The mixture was slowly raised to rt and a saturated solution of NH_4Cl (3 mL) was added and the mixture was extracted with EtOAc (2 × 10 mL). The organic phases were combined, washed with brine (15 mL) and dried over Na_2SO_4. After filtration and concentration under vacuum, the crude was purified by column chromatography on silica gel (cyclohexane/EtOAc 95:5).

[10] Sakurada, T.; Sugiyama, Y.-k., Okamoto, S. *J. Org. Chem.* **2013**, *78*, 3583–3591.

cis*-1b-*d. ^{1}H NMR (300 MHz, $CDCl_3$) δ 7.48-7.41 (m, 4H), 7.32 (d, *J* = 9.0 Hz, 2H), 7.29-7.25 (m, 4H), 7.22-7.16 (m, 2H), 6.80 (d, *J* = 9.0, 2H), 3.77 (s, 3H), 3.42 (d, *J* = 3.8 Hz, 1H), 3.40 (d, *J* = 6.0 Hz, 1H), 3.24 (dd, *J* = 6.0, 3.9 Hz, 1H), 1.15-0.95 (m, 21H).

trans*-1b-*d. ^{1}H NMR (300 MHz, $CDCl_3$) δ 7.47-7.41 (m, 4H), 7.34-7.18 (m, 8H), 6.83 (d, *J* = 9.0 Hz, 2H), 3.79 (s, 3H), 3.39 (dd, *J* = 11.0, 2.5 Hz, 1H), 3.30 (dd, *J* = 4.5, 2.5 Hz, 1H), 3.14 (dd, *J* = 11.0, 4.5 Hz, 1H), 1.05 (m, 21H).

1-((4-Methoxyphenyl)diphenylmethoxy)-5-(triisopropylsilyl)pent-4-yn-2-one (14). ^{1}H NMR (500 MHz, $CDCl_3$) δ 7.44 (d, J = 7.5 Hz, 4H), 7.35-7.27 (d, 6H), 7.25-7.21 (m, 2H), 6.83 (d, J = 9.0 Hz, 2H), 4.02 (s, 2H), 3.79 (s, 3H), 3.43 (s, 2H), 1.15-0.95 (m, 21H). ^{13}C NMR (126 MHz, $CDCl_3$) δ 201.4, 158.9, 143.8, 134.8, 130.5, 128.4, 128.2, 127.3, 113.4, 99.1, 87.3, 86.1, 68.9, 55.4, 32.9, 18.7, 11.3. HRMS (ESI-TOF) *m*/*z* $[M + H]^+$ Calcd for $C_{34}H_{42}NaO_3Si$ 549.2795; Found 549.2780.
