# Supplementary material for: Borylation and rearrangement of alkynyloxiranes: a stereospecific route to substituted α-enynes
Source: Beilstein J Org Chem. 2019 Jun 27;15:1416–24. doi: 10.3762/bjoc.15.141 (PMC6604752; doi:10.3762/bjoc.15.141)

BEILSTEIN JOURNAL OF ORGANIC CHEMISTRY

Supporting Information

for

Borylation and rearrangement of alkynyloxiranes: a stereospecific route to substituted α-enynes

Ruben Pomar Fuentespina, José Luis Angel Garcia de la Cruz, Gabriel Durin, Victor Mamane, Jean-Marc Weibel and Patrick Pale

Beilstein J. Org. Chem. **2019,** *15,* 1416–1424. doi:10.3762/bjoc.15.141

Copies of ^{1}H NMR and ^{13}C NMR spectra of all new compounds

License and Terms: This is a supporting information file under the terms of the Creative Commons Attribution License (http://creativecommons.org/licenses/by/4.0). Please note that the reuse, redistribution and reproduction in particular requires that the authors and source are credited.
The license is subject to the *Beilstein Journal of Organic Chemistry* terms and conditions: (https://www.beilstein-journals.org/bjoc)

Triisopropyl(4-methylpent-3-en-1-yn-1-yl)silane (A)

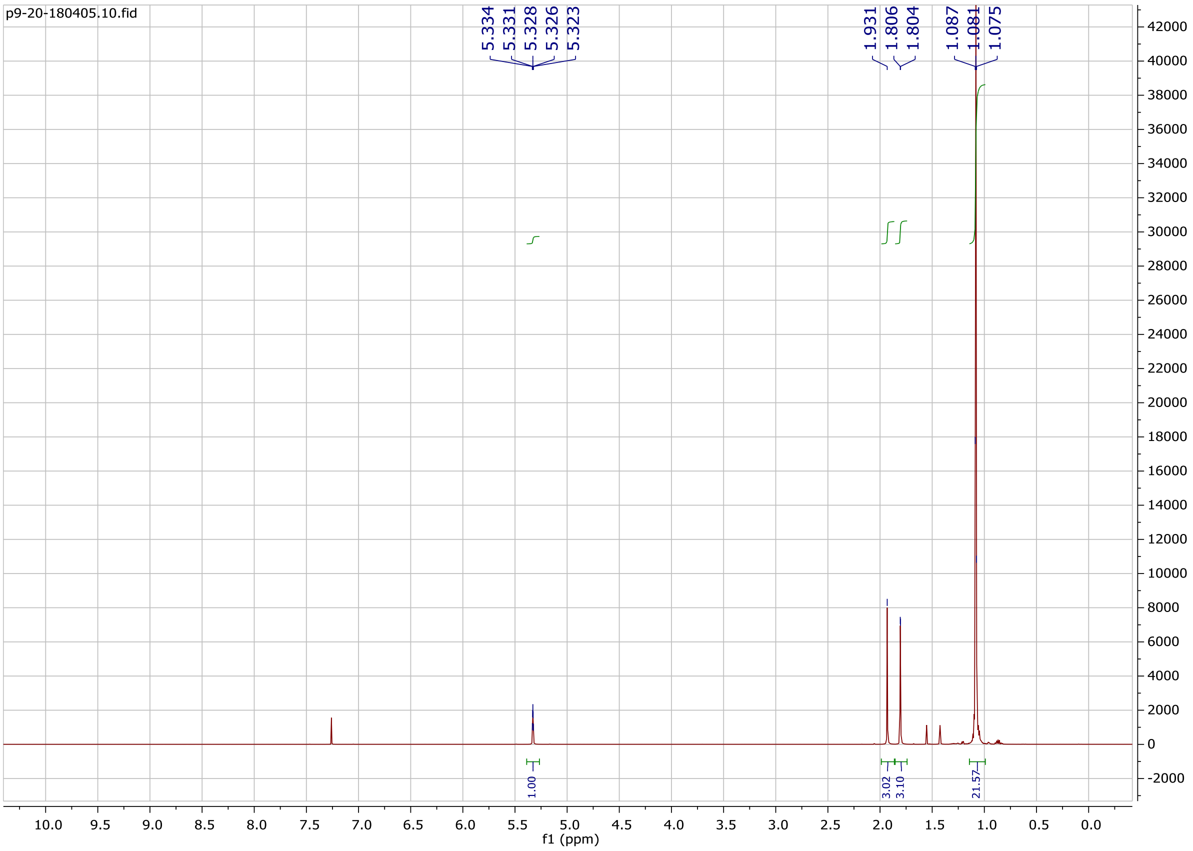

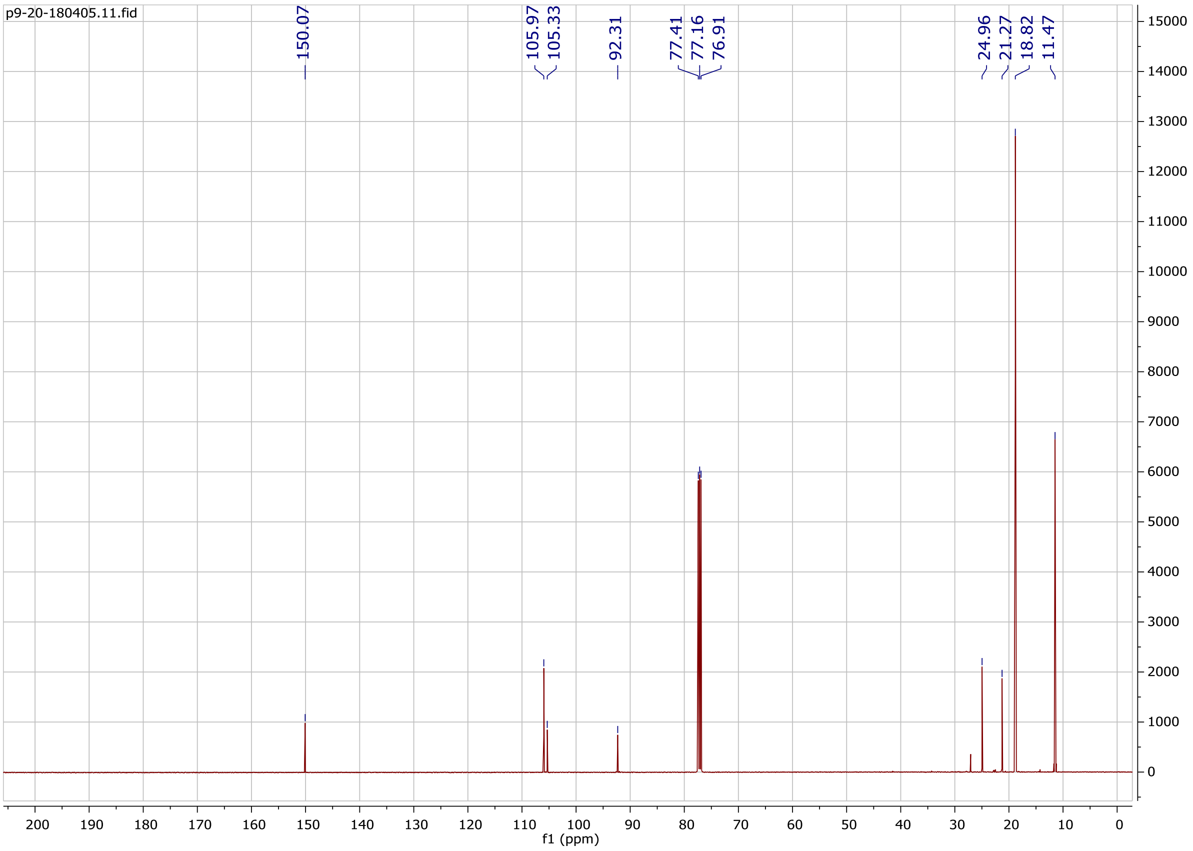

((3,3-Dimethyloxiran-2-yl)ethynyl)triisopropylsilane (1a)

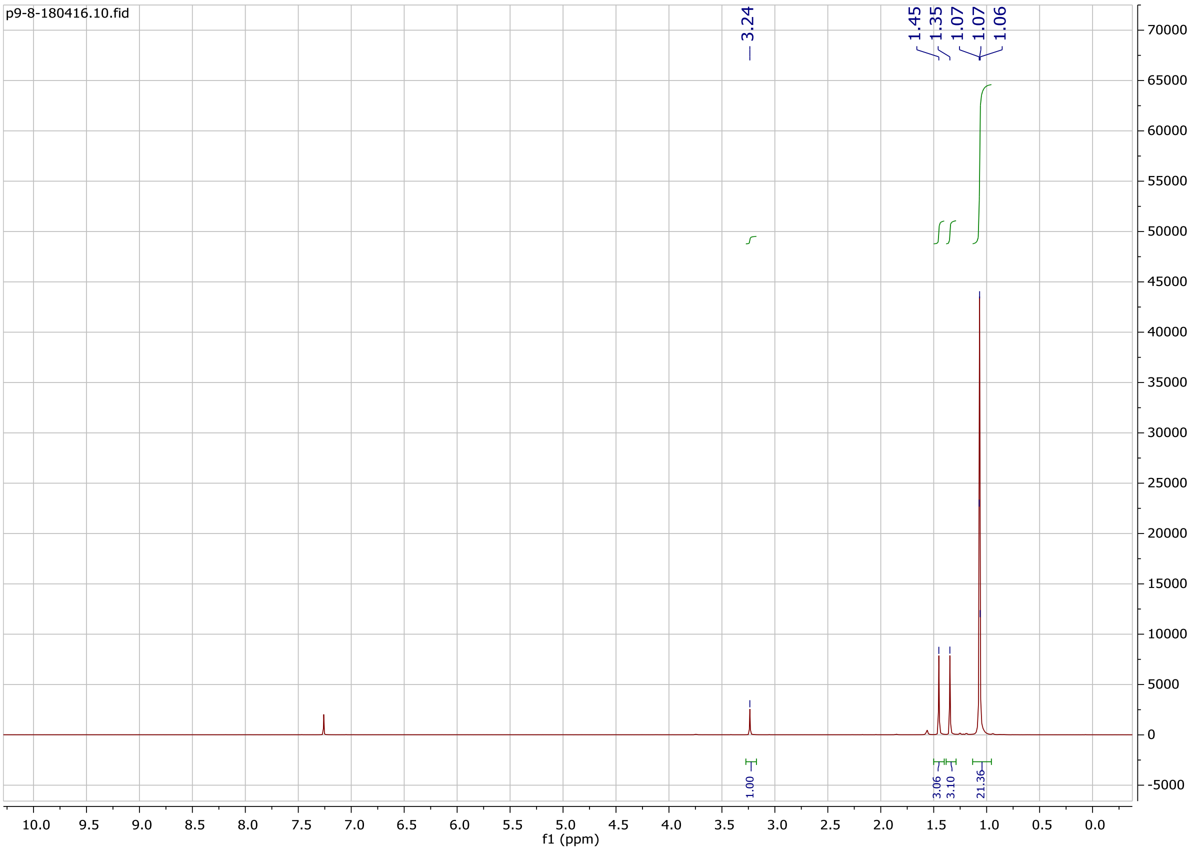

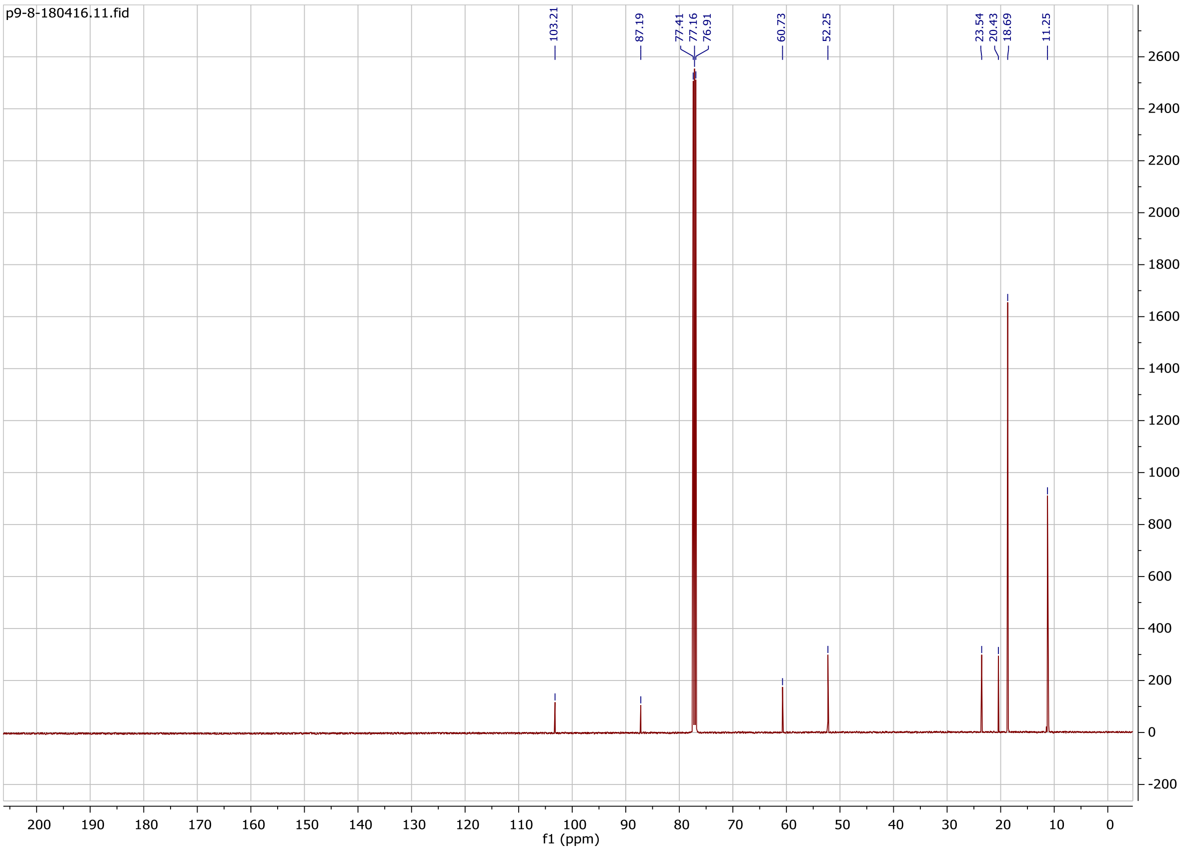

Triisopropyl(4-methyl-3-phenylpent-3-en-1-yn-1-yl)silane (3)

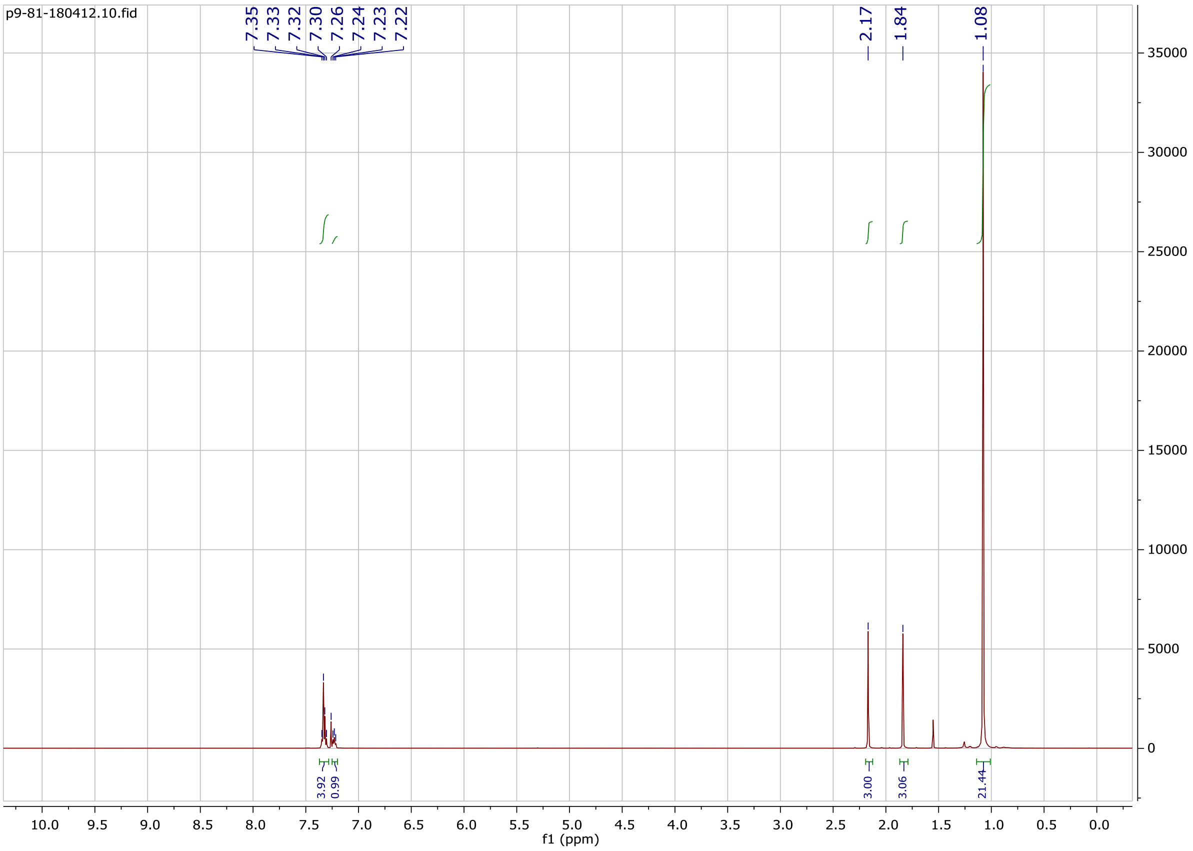

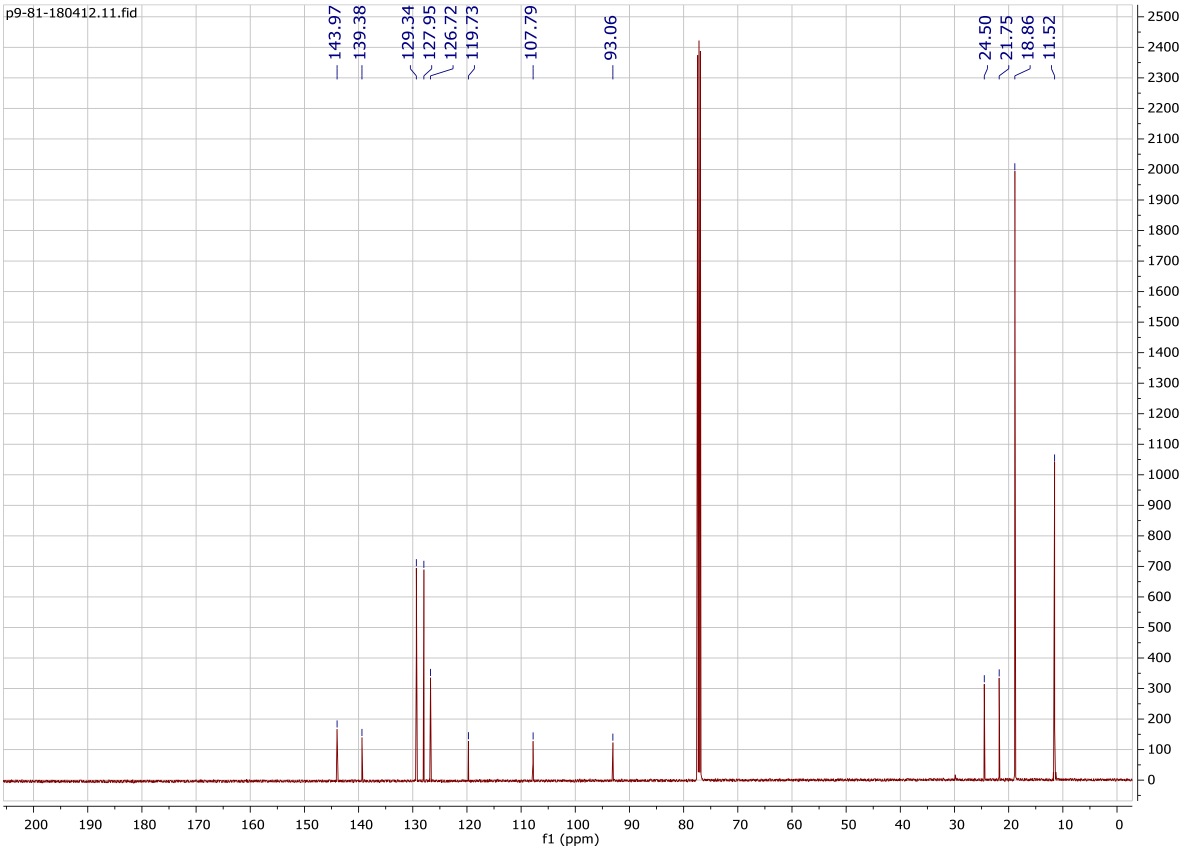

***trans*-Triisopropyl((3-(((4-methoxyphenyl)diphenylmethoxy)methyl)oxiran-2-yl)ethynyl)silane (*trans*-1b)**

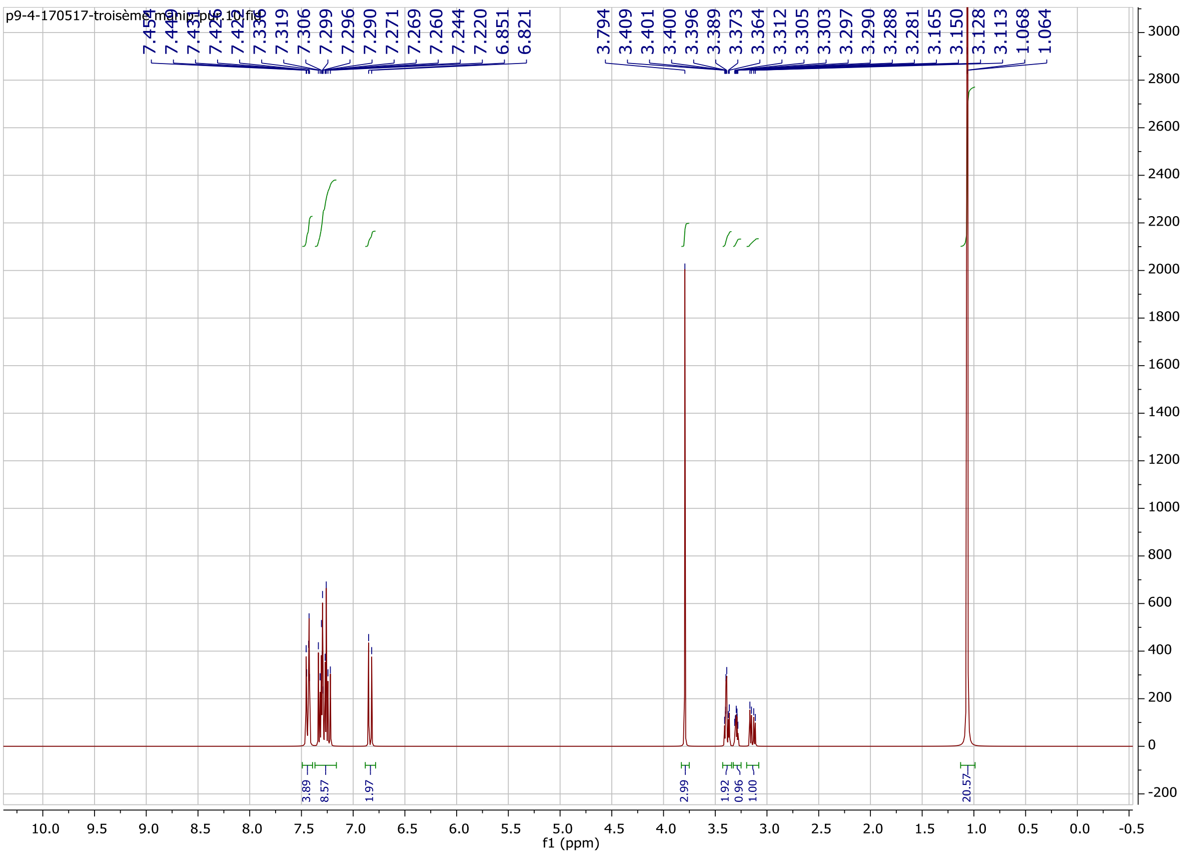

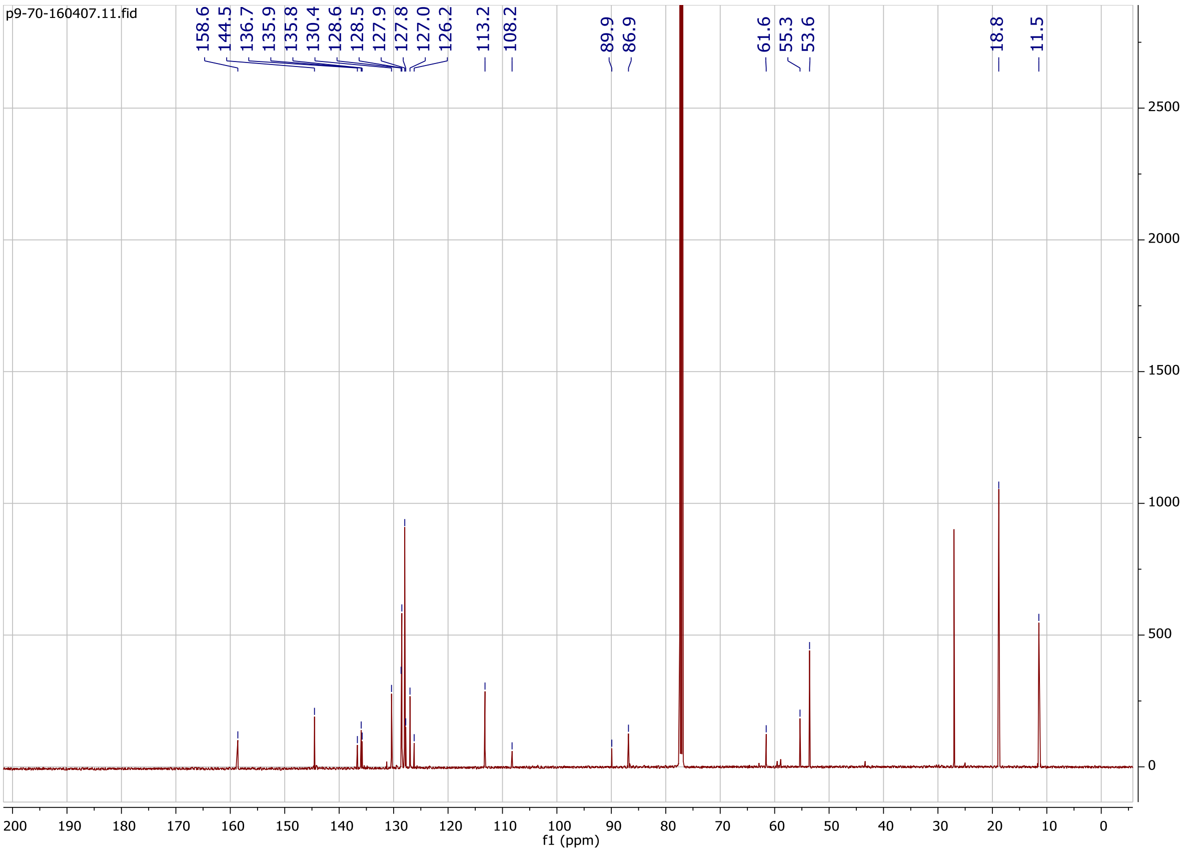

Ethyl (*Z*)-5-(triisopropylsilyl)pent-2-en-4-ynoate (G)

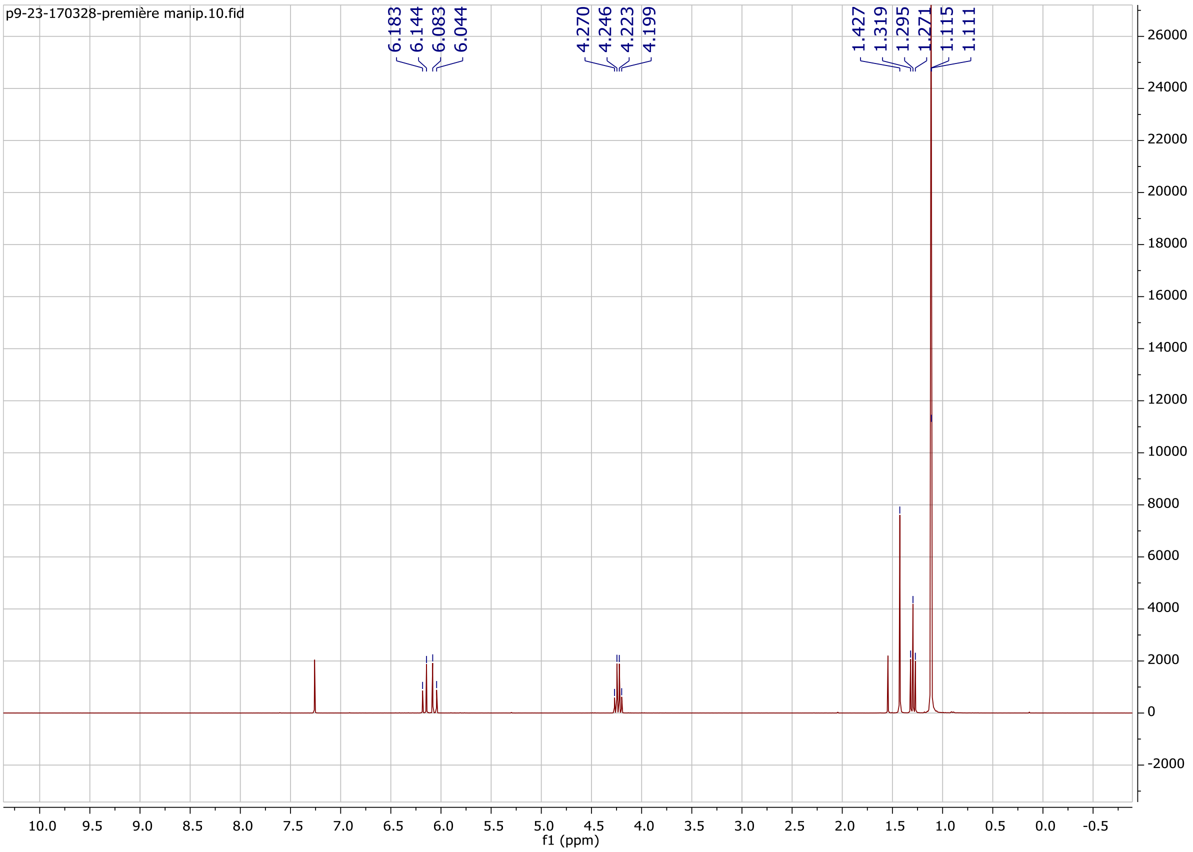

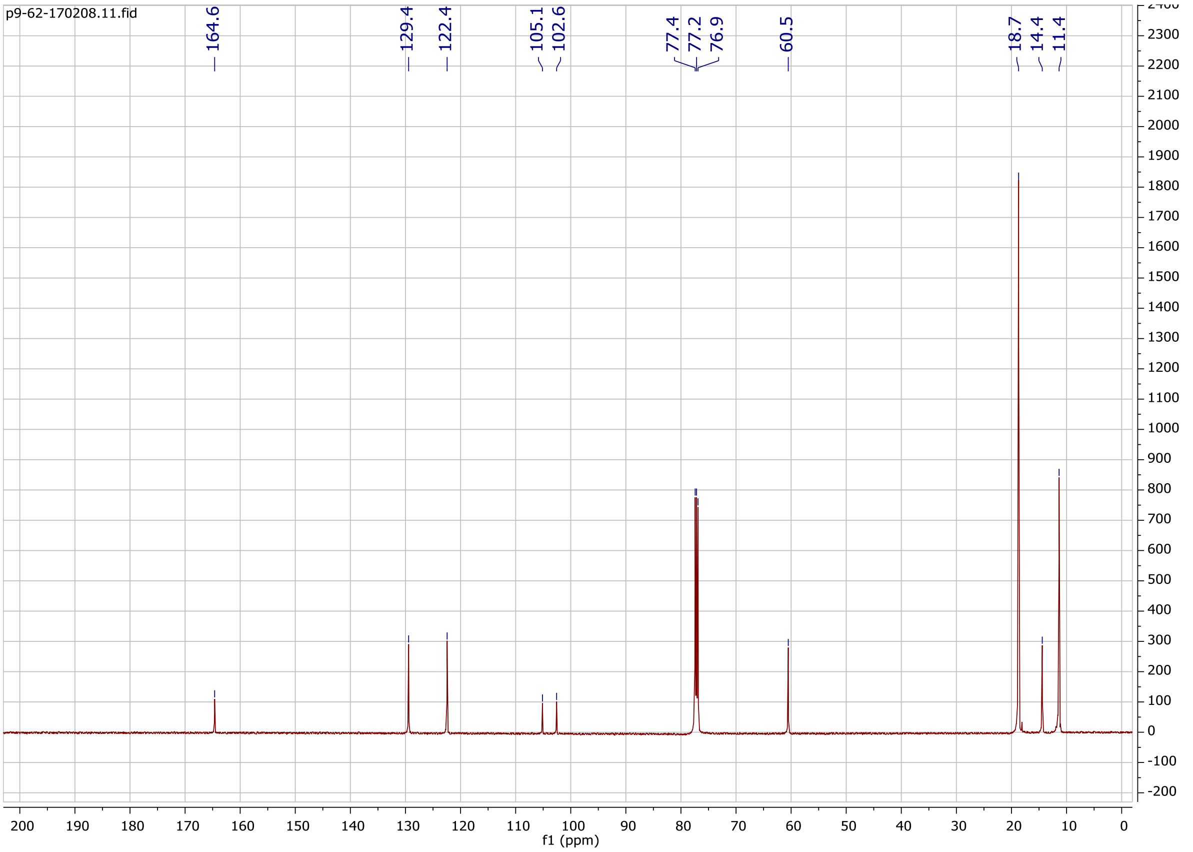

(*Z*)-5-(Triisopropylsilyl)pent-2-en-4-yn-1-ol (H)

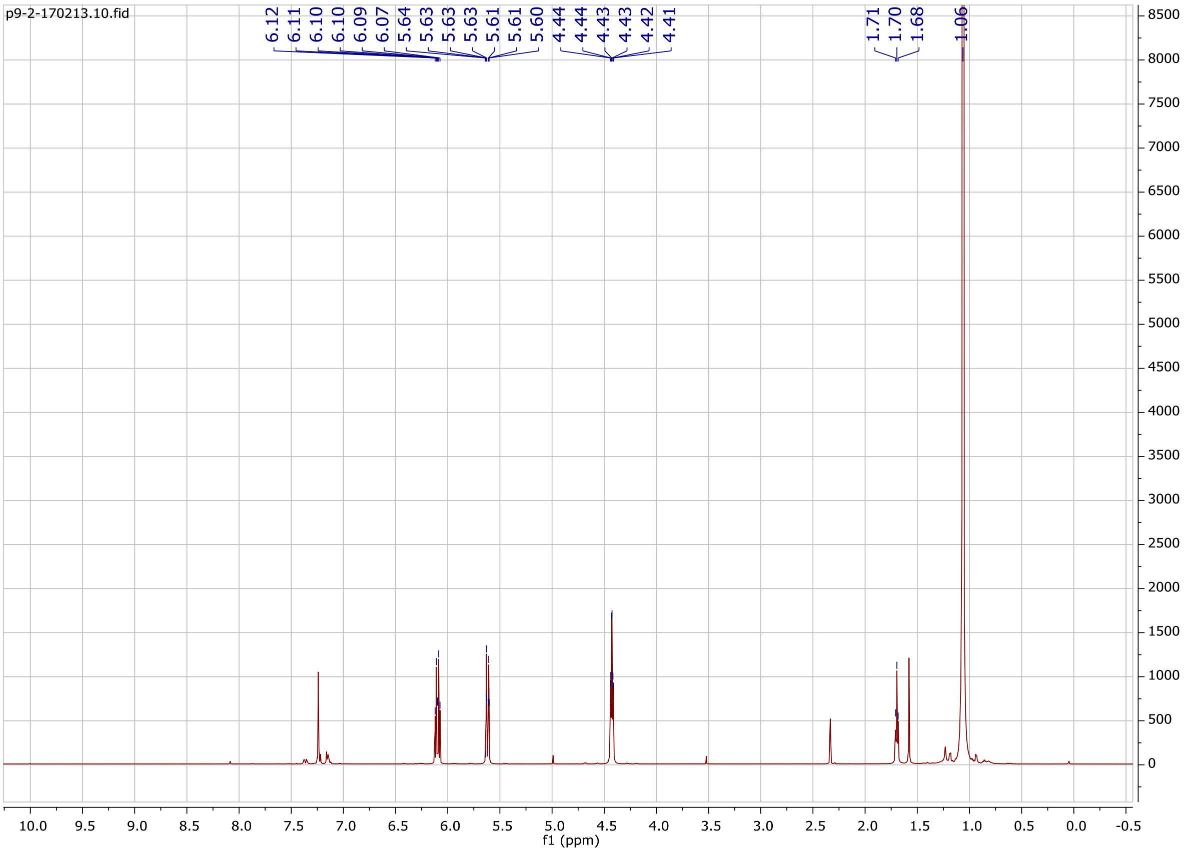

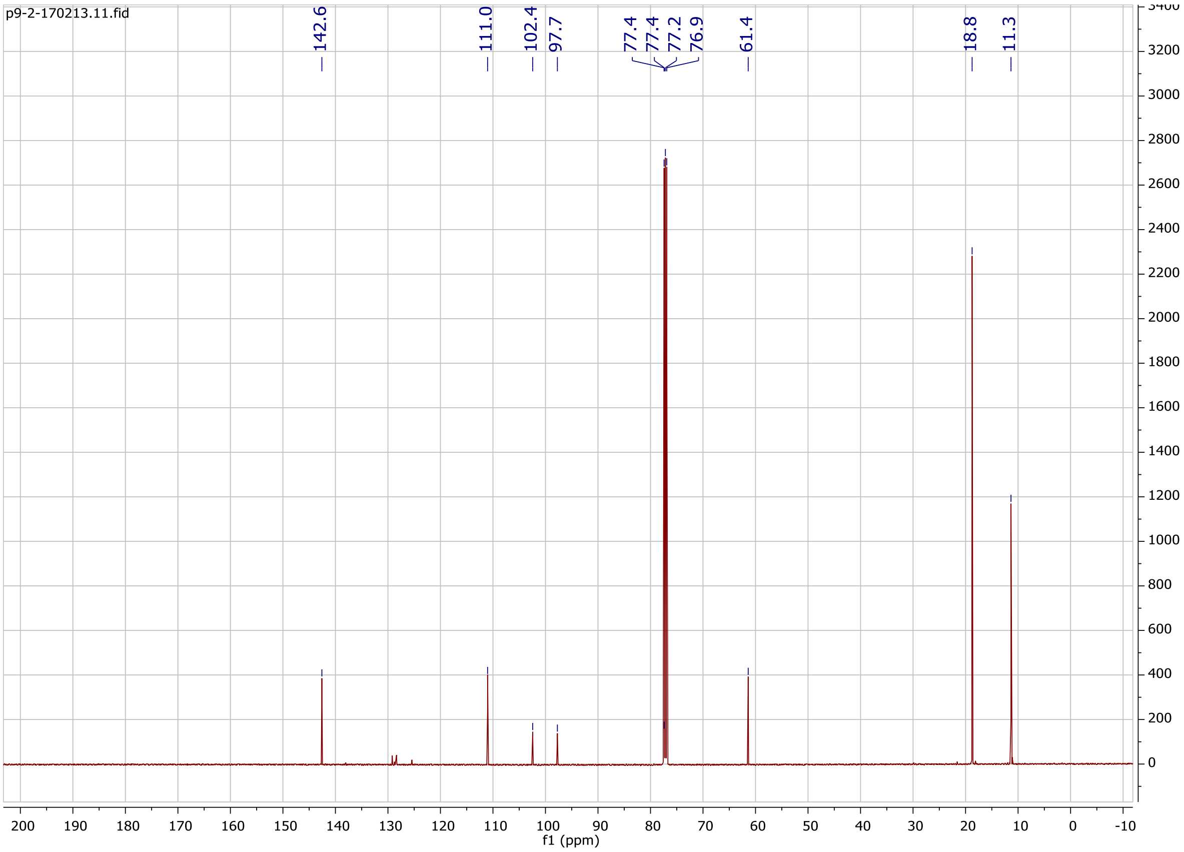

cis-(3-((Triisopropylsilyl)ethynyl)oxiran-2-yl)methanol (I)

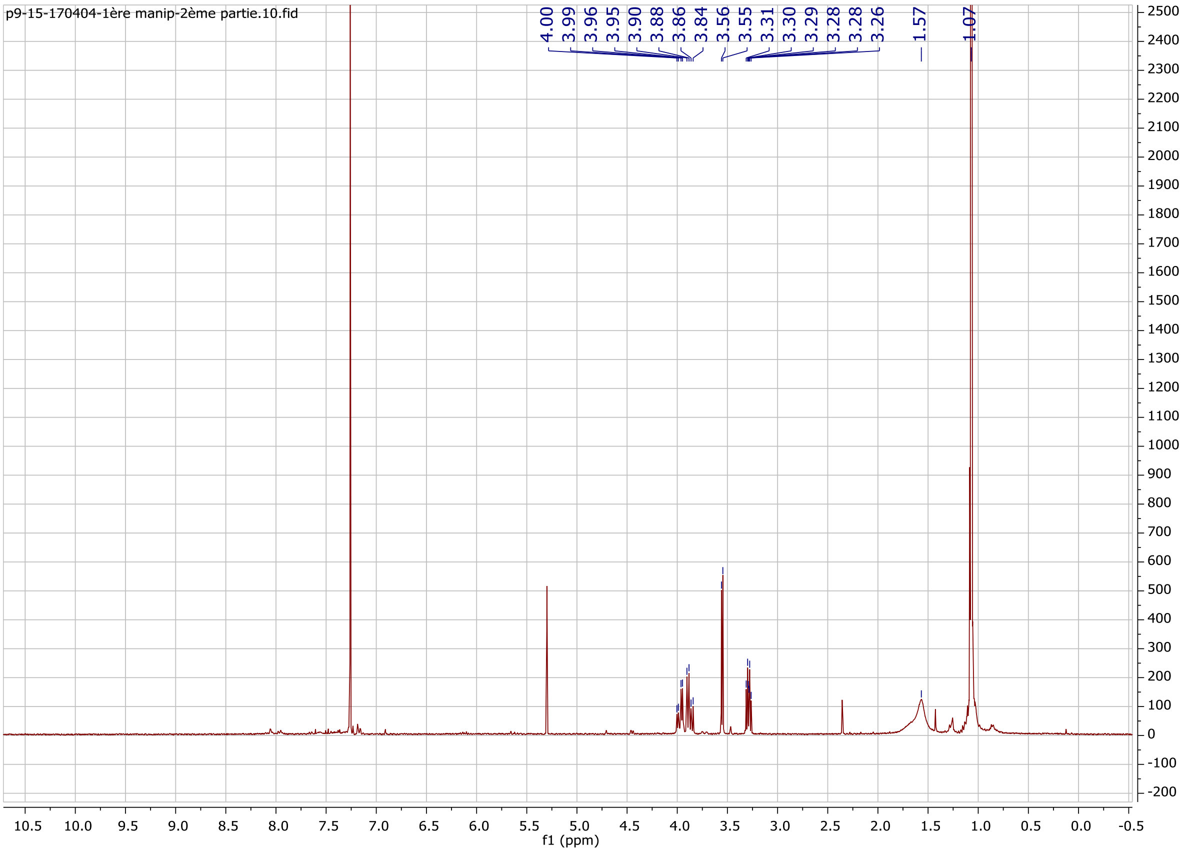

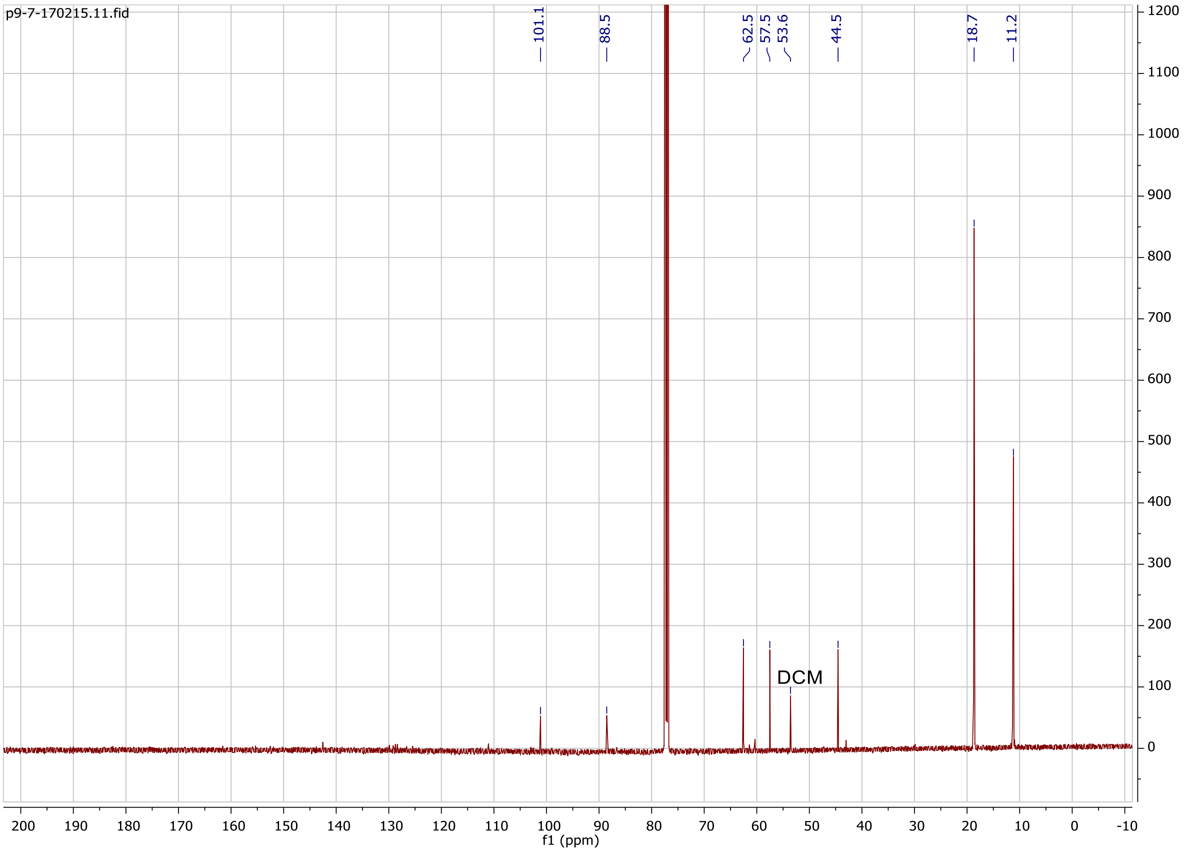

***cis*-Triisopropyl((3-(((4-methoxyphenyl)diphenylmethoxy)methyl)oxiran-2-yl)ethynyl)silane (*cis*-1d)**

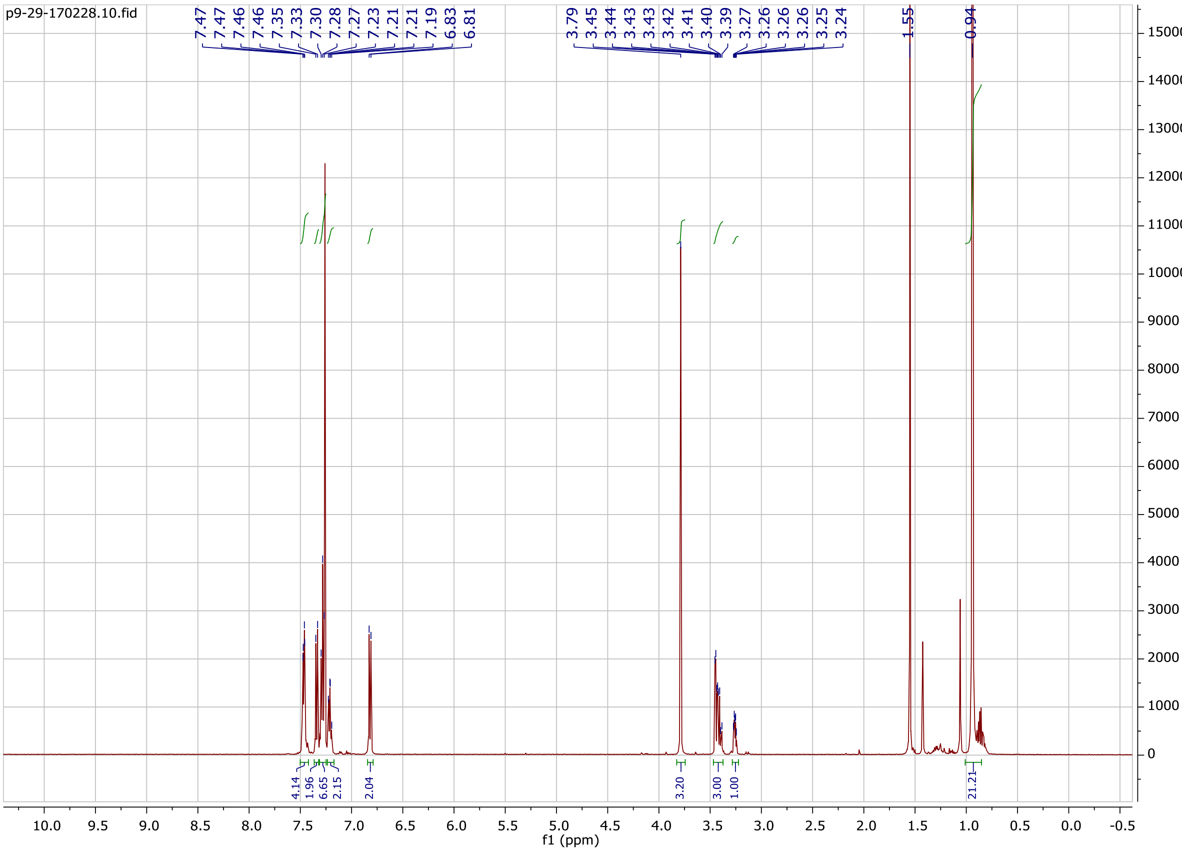

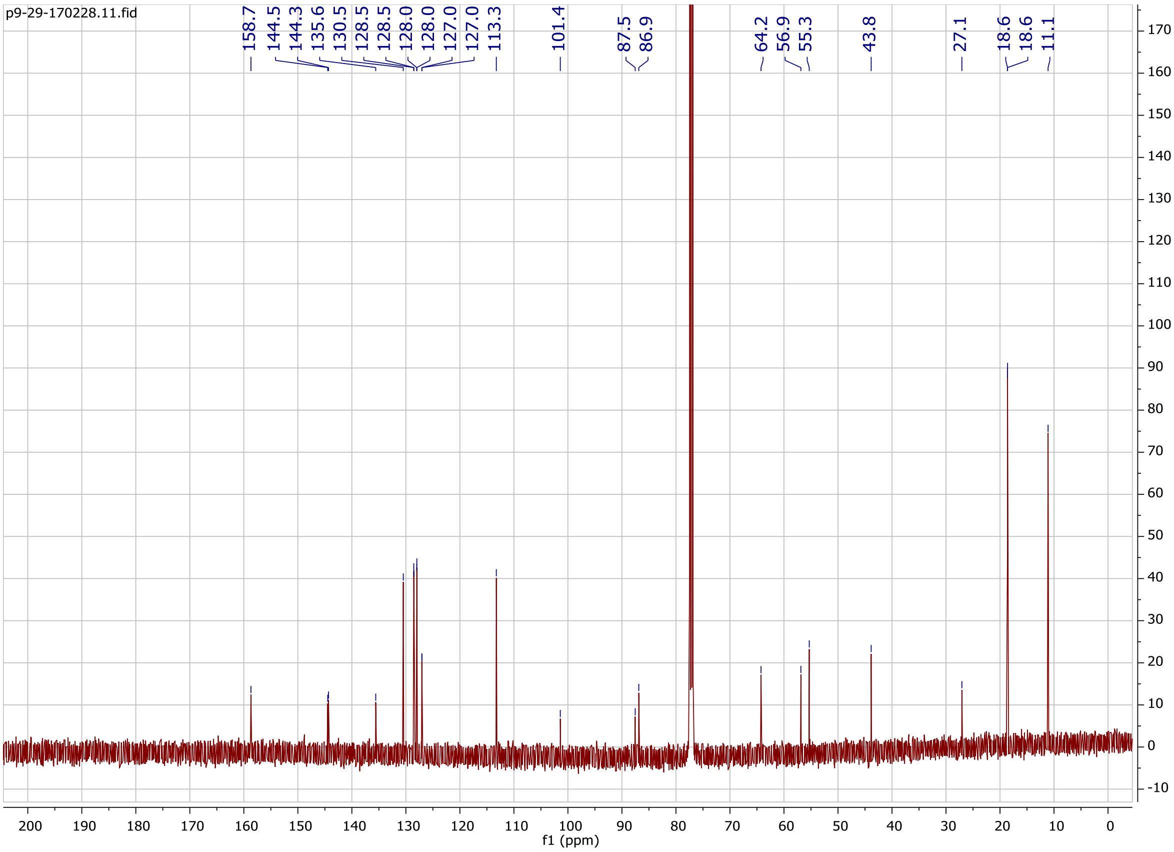

(*Z*)-Triisopropyl(5-((4-methoxyphenyl)diphenylmethoxy)-3-phenylpent-3-en-1-yn-1-yl)silane ((*Z*)-4)

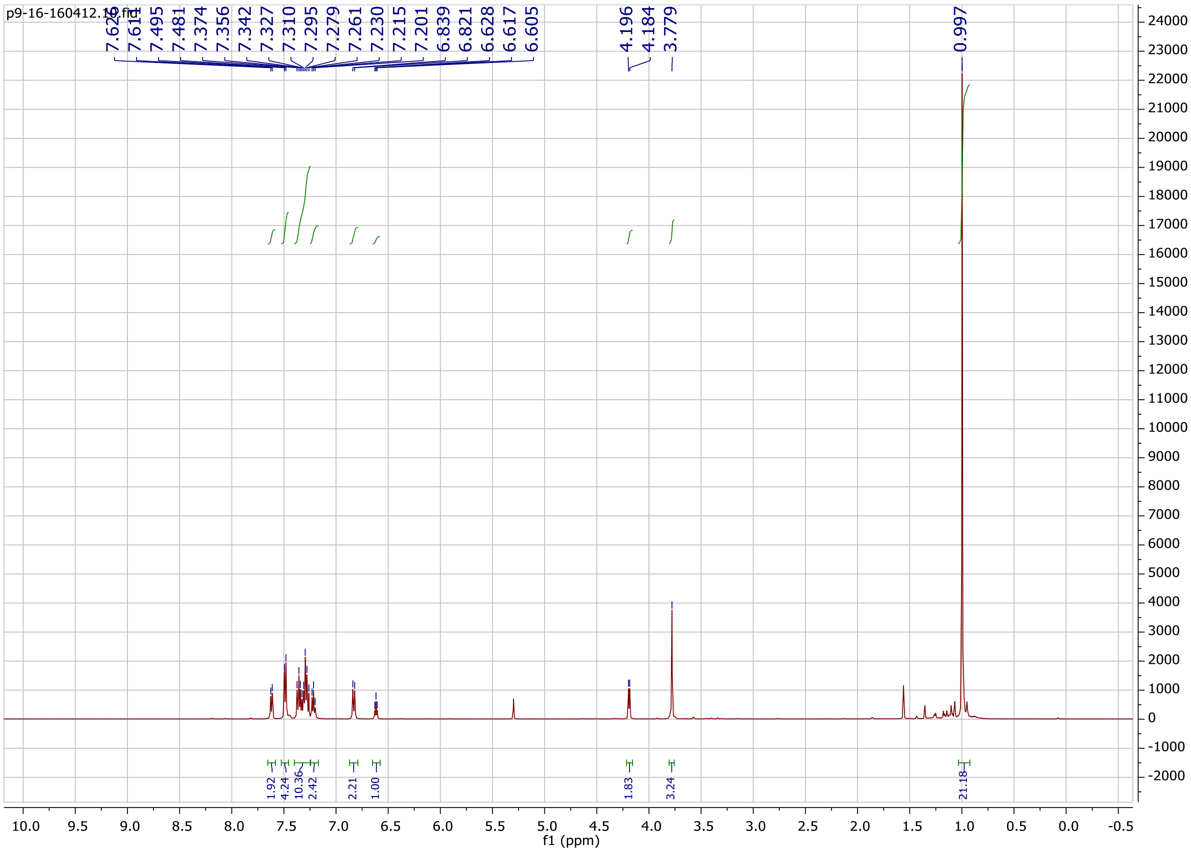

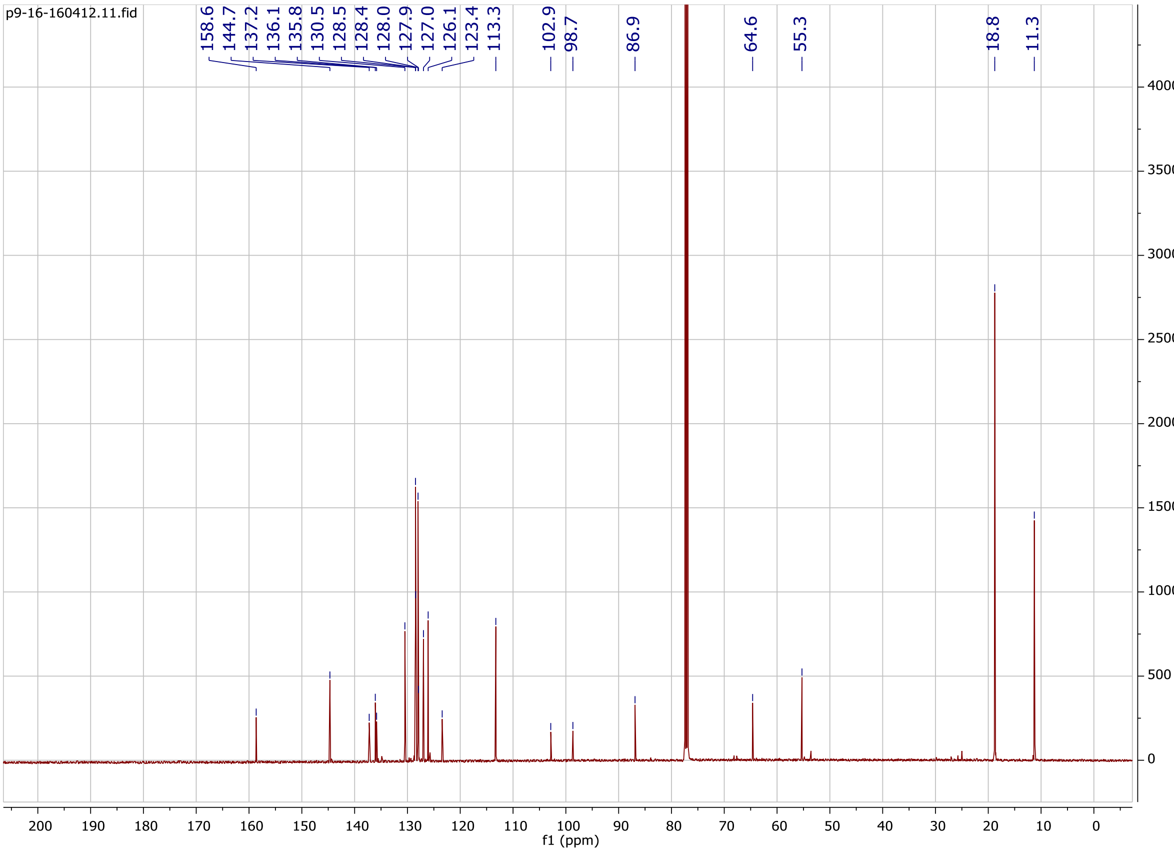

(*E*)-Triisopropyl(5-((4-methoxyphenyl)diphenylmethoxy)-3-phenylpent-3-en-1-yn-1-yl)silane ((*E*)-4)

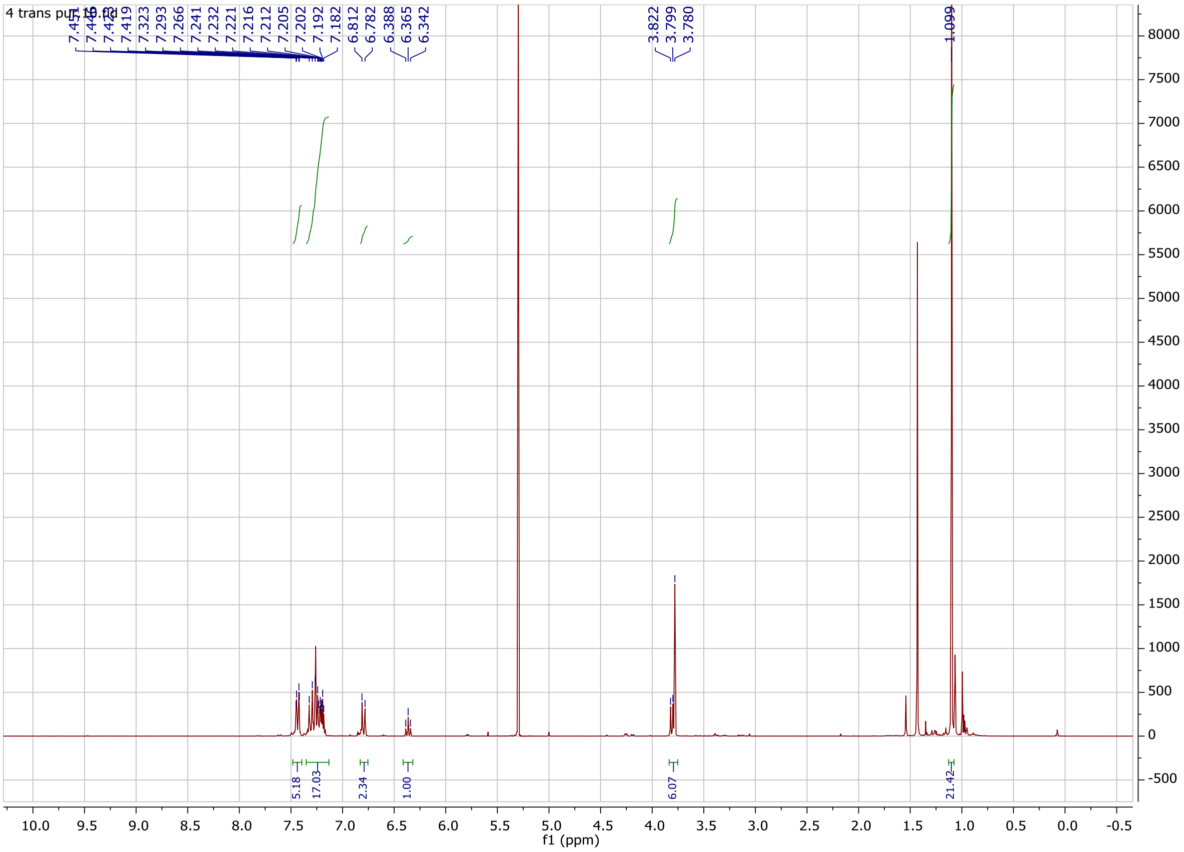

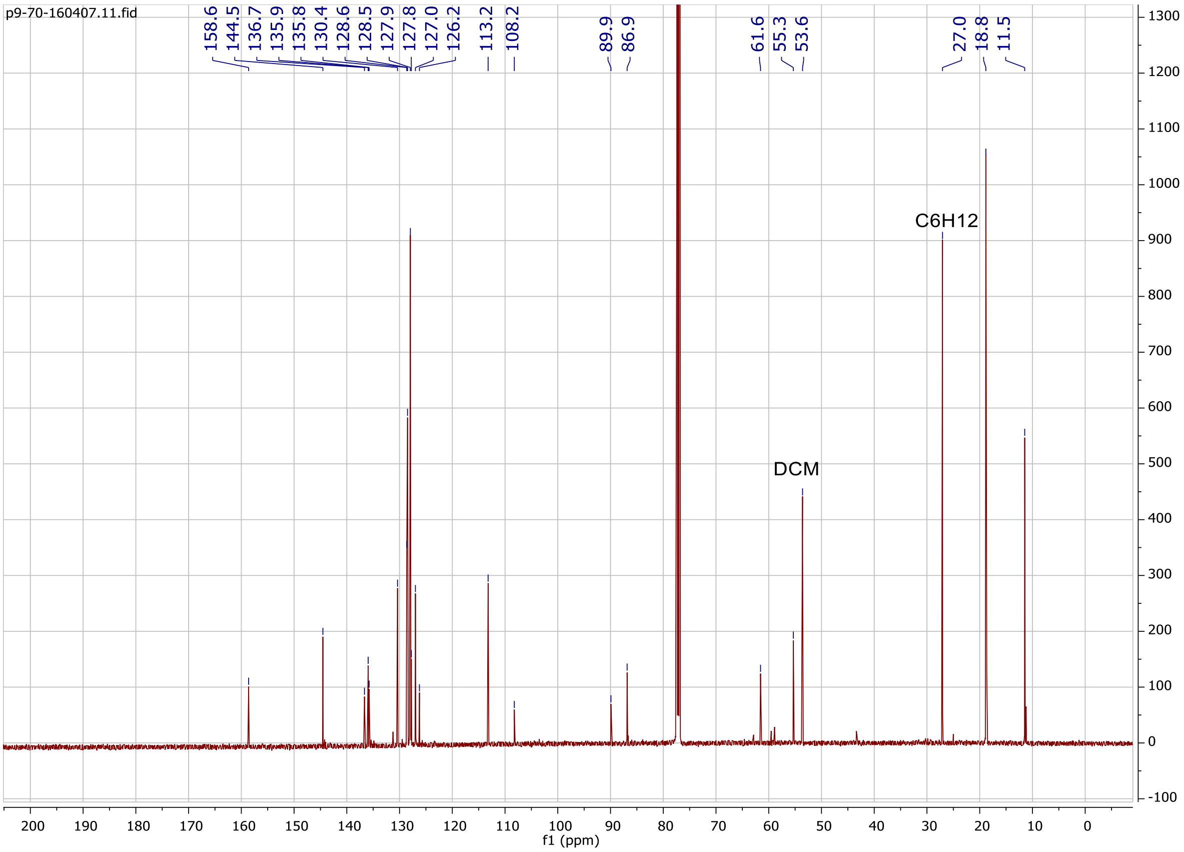

(*Z*)-Triisopropyl(3-(4-methoxyphenyl)-5-((4-methoxyphenyl)diphenylmethoxy)pent-3-en-1-yn-1-yl)silane ((*Z*)-5)

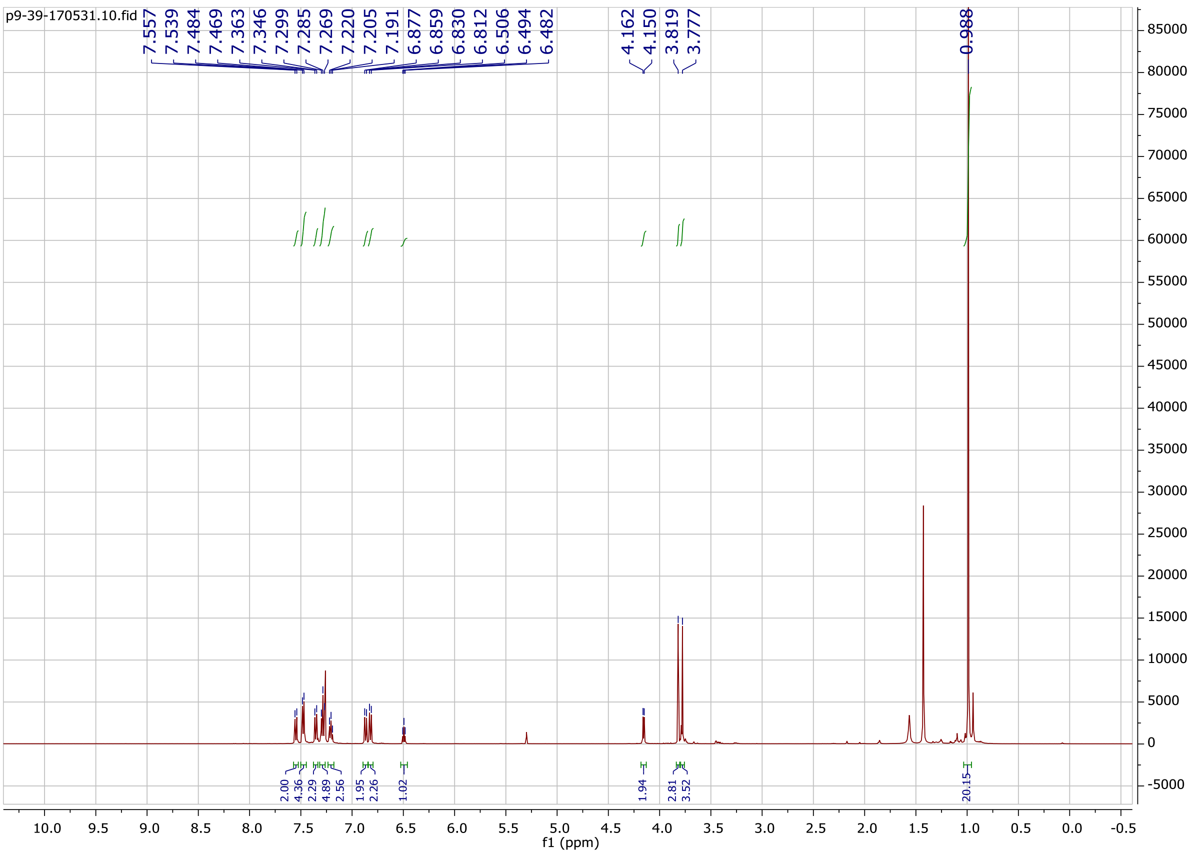

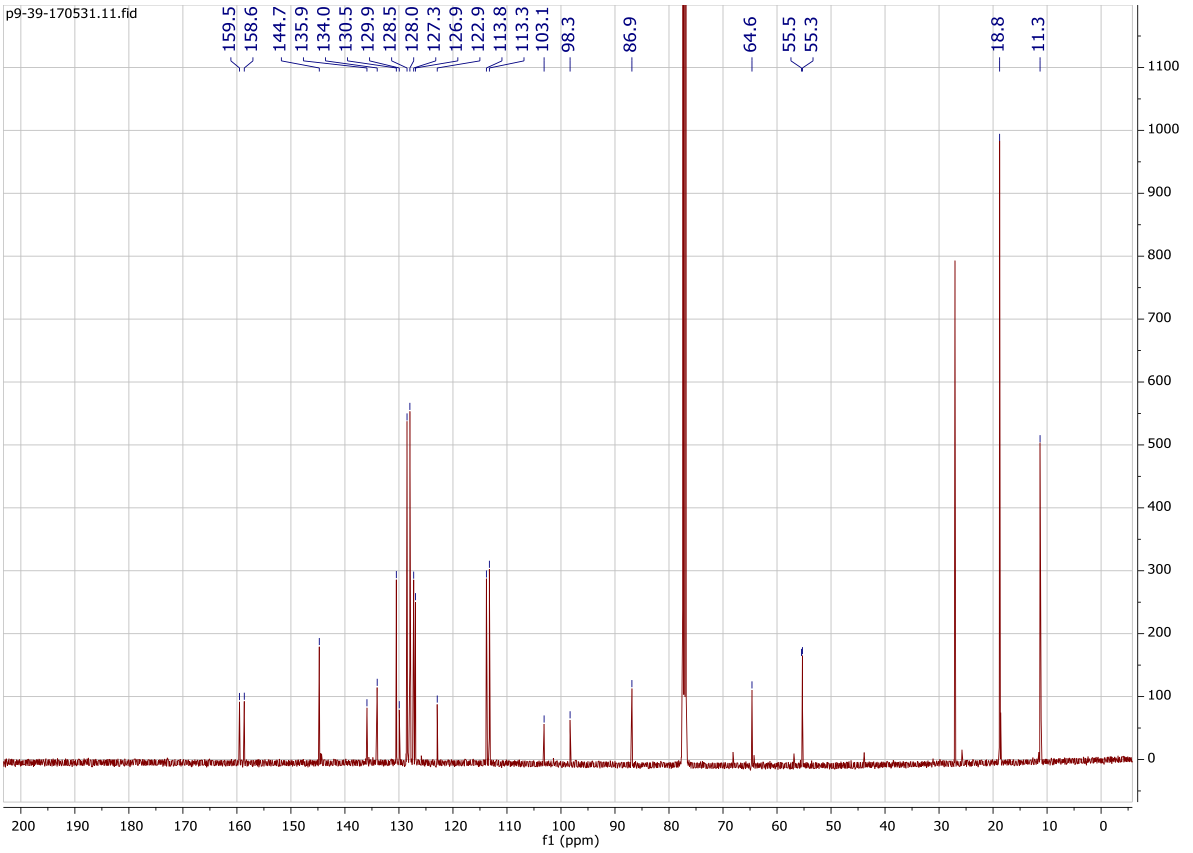

(*E*)-Triisopropyl(3-(4-methoxyphenyl)-5-((4-methoxyphenyl)diphenylmethoxy)pent-3-en-1-yn-1-yl)silane ((*E*)-5)

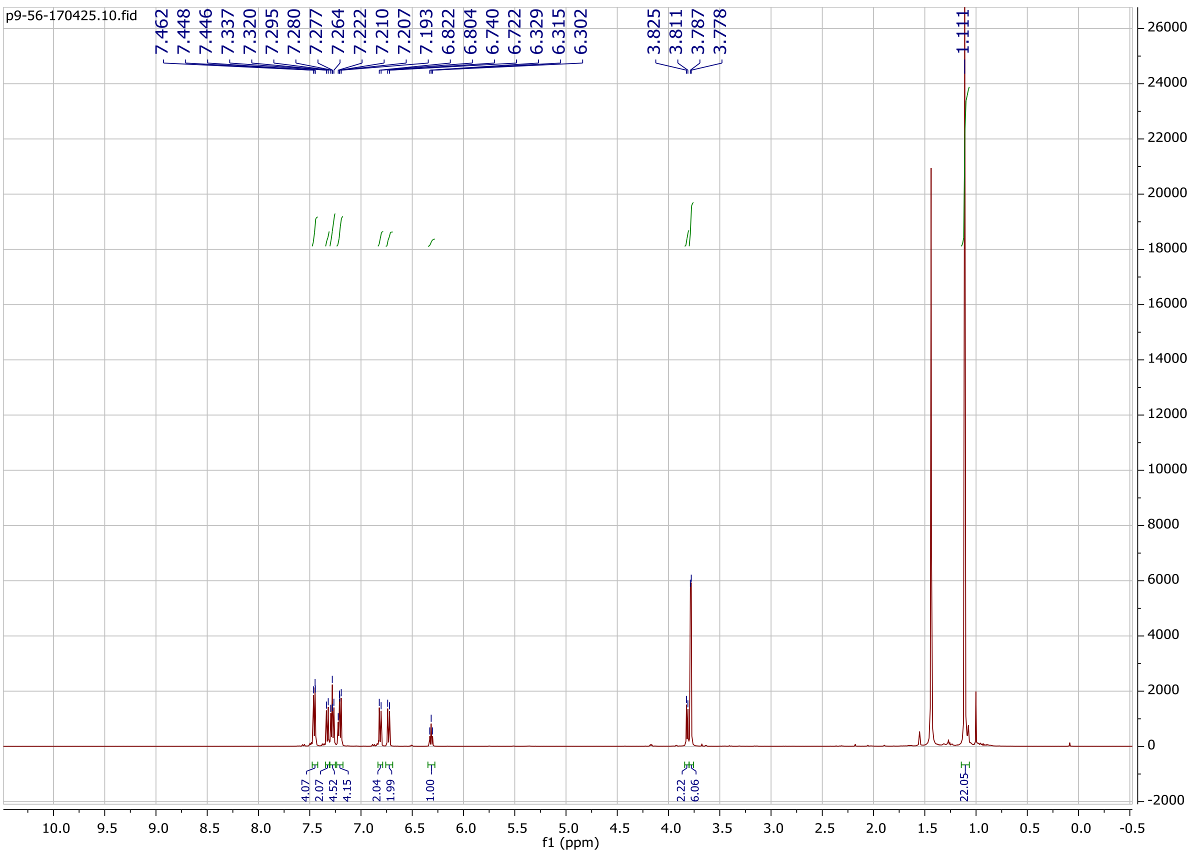

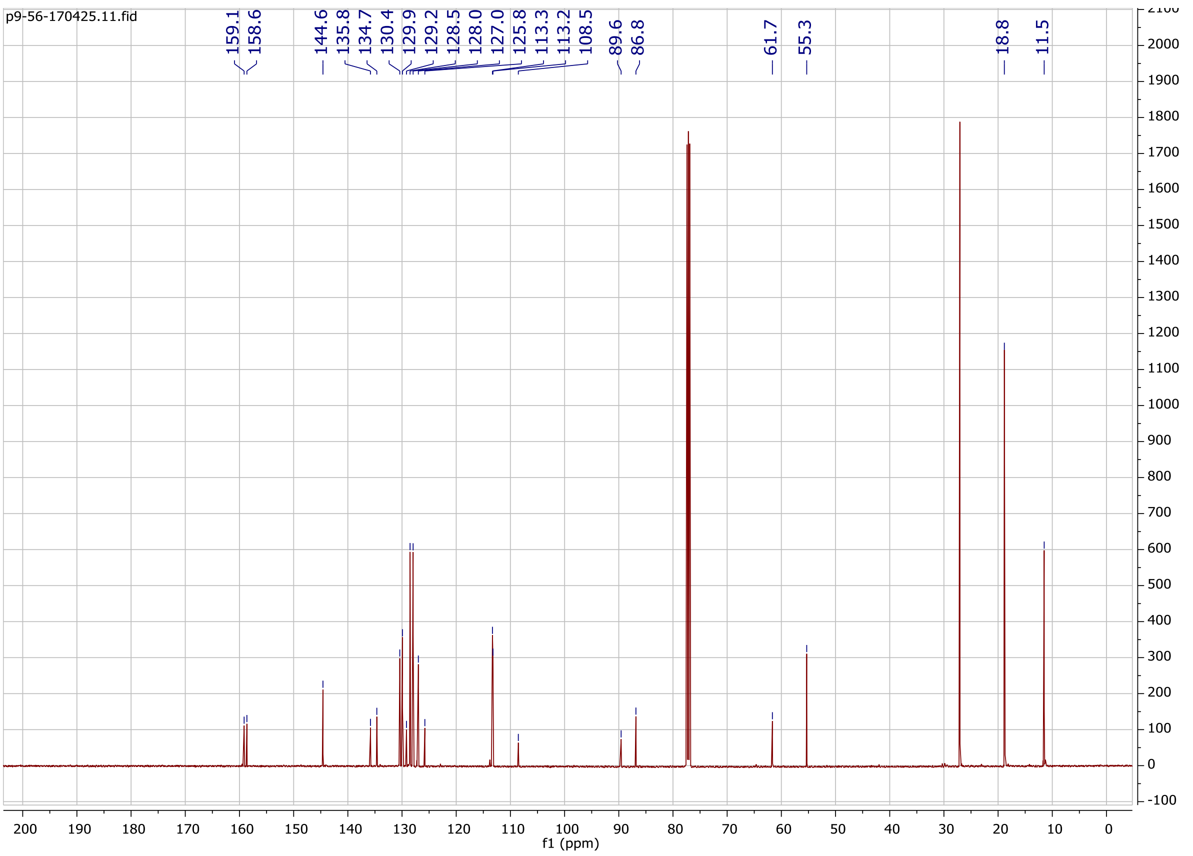

(*Z*)-(3-(4-Chlorophenyl)-5-((4-methoxyphenyl)diphenylmethoxy)pent-3-en-1-yn-1-yl)triisopropylsilane ((*Z*)-6)

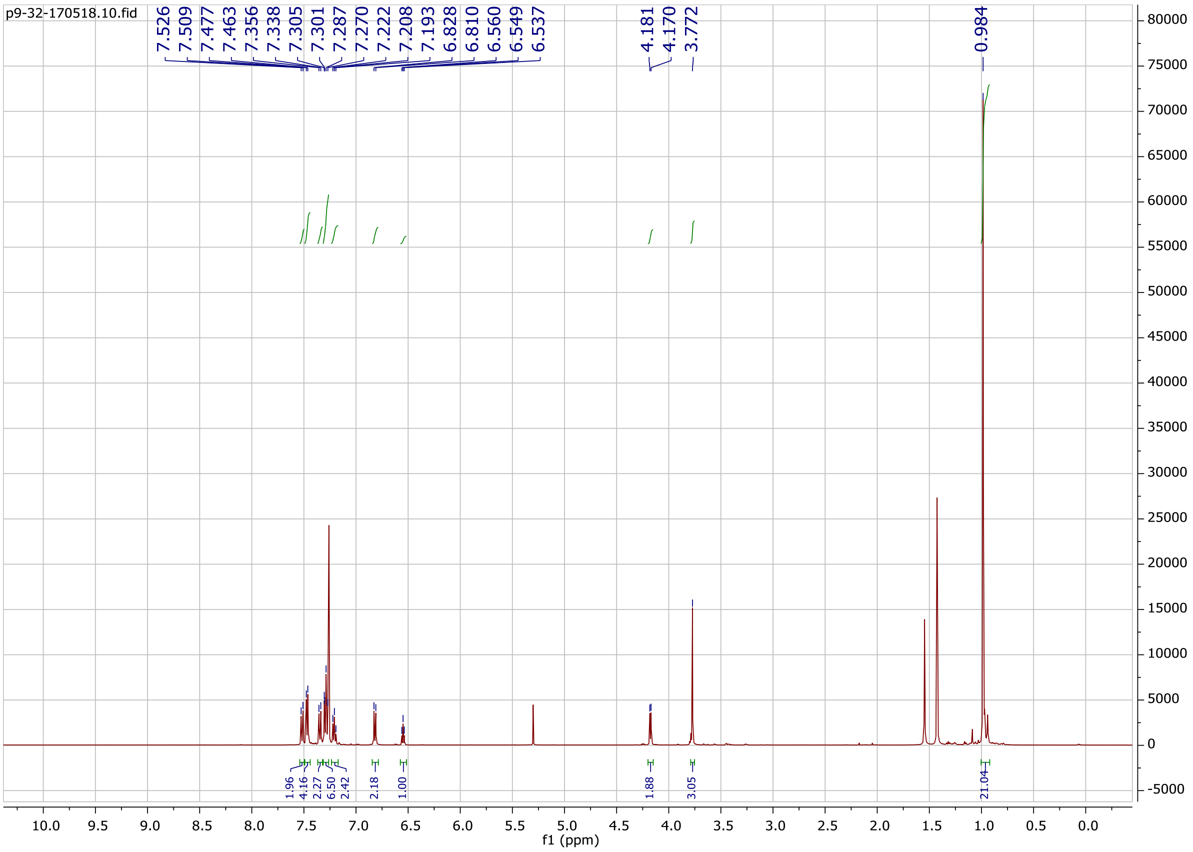

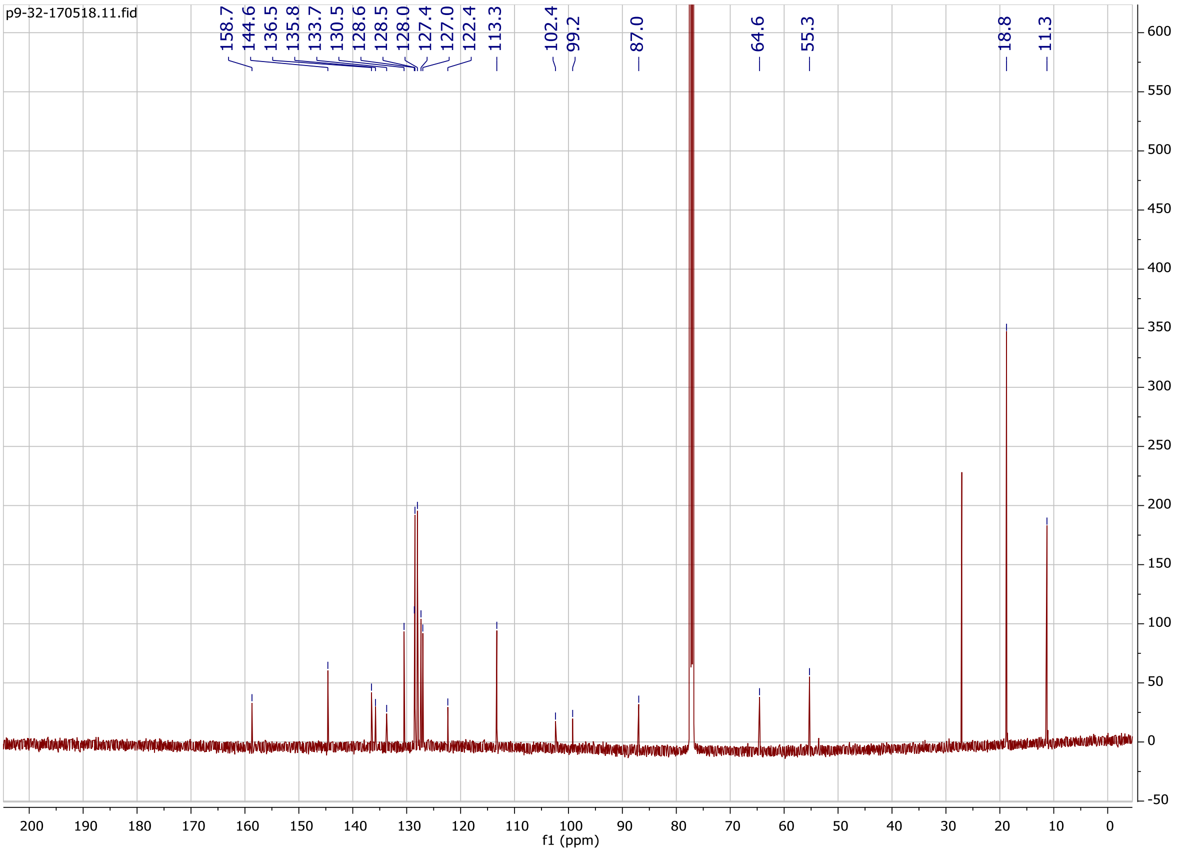

(*E*)-(3-(4-Chlorophenyl)-5-((4-methoxyphenyl)diphenylmethoxy)pent-3-en-1-yn-1-yl)triisopropylsilane ((*E*)-6)

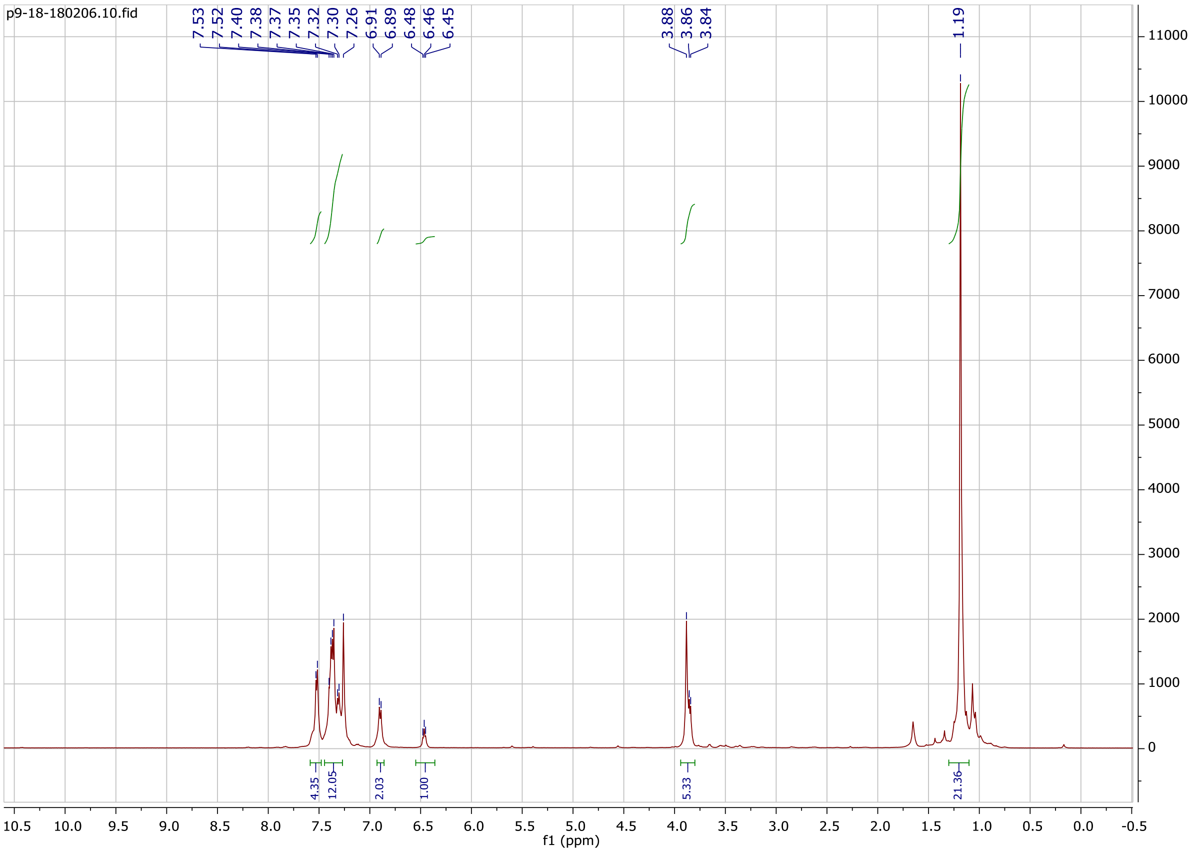

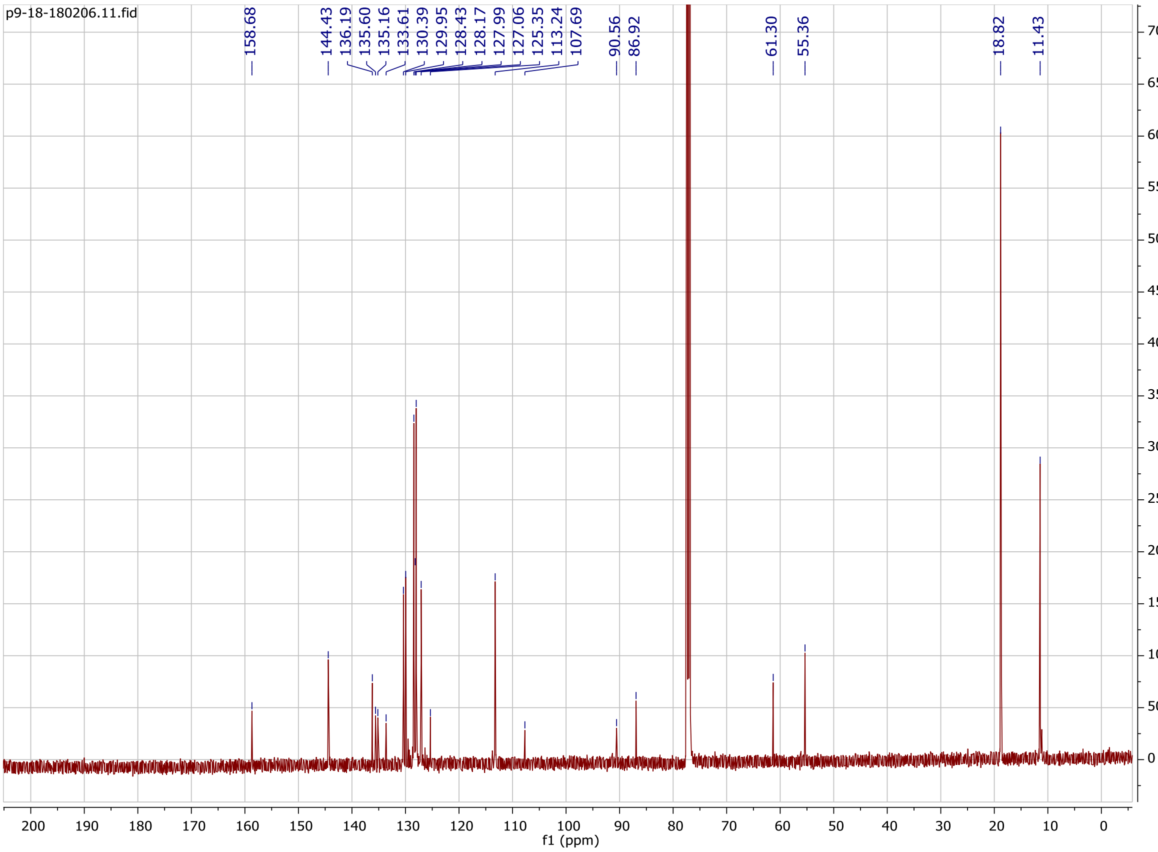

(*Z*)-(3-Benzyl-5-((4-methoxyphenyl)diphenylmethoxy)pent-3-en-1-yn-1-yl)triisopropylsilane ((*Z*)-7)

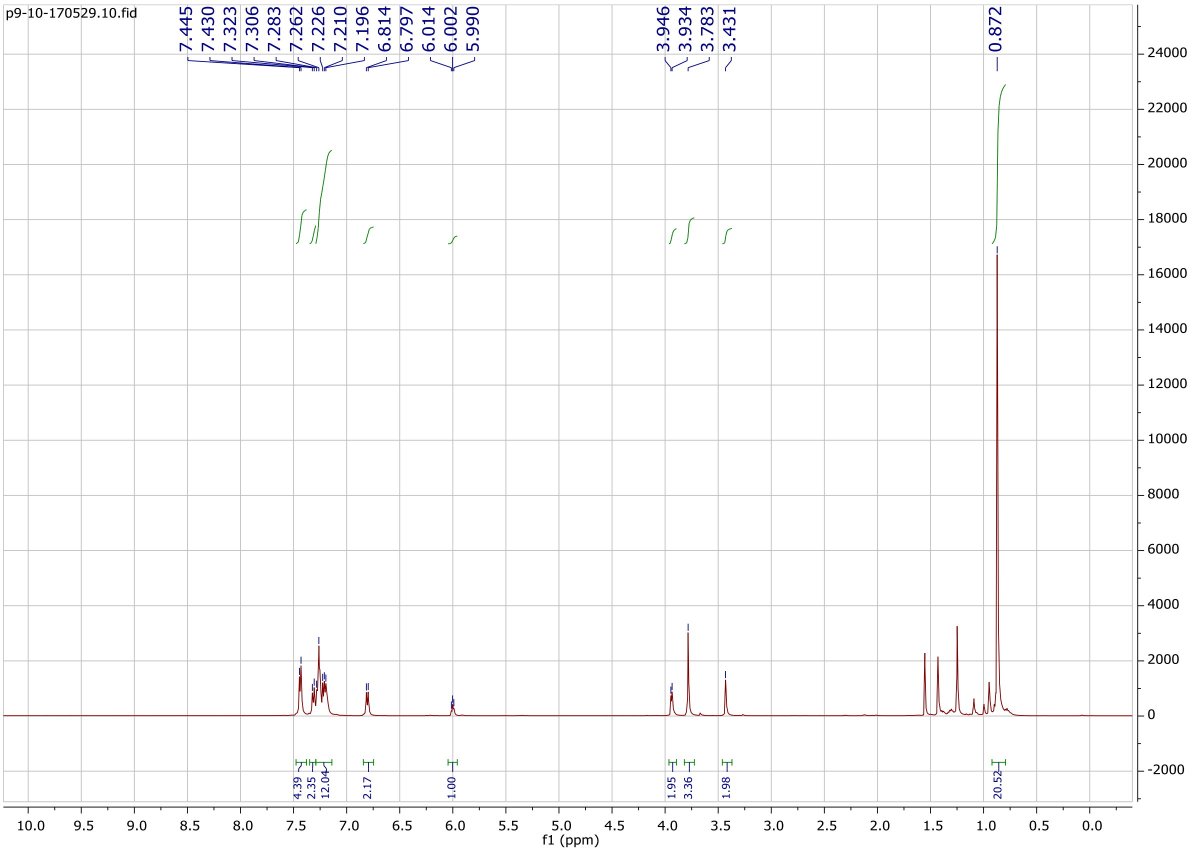

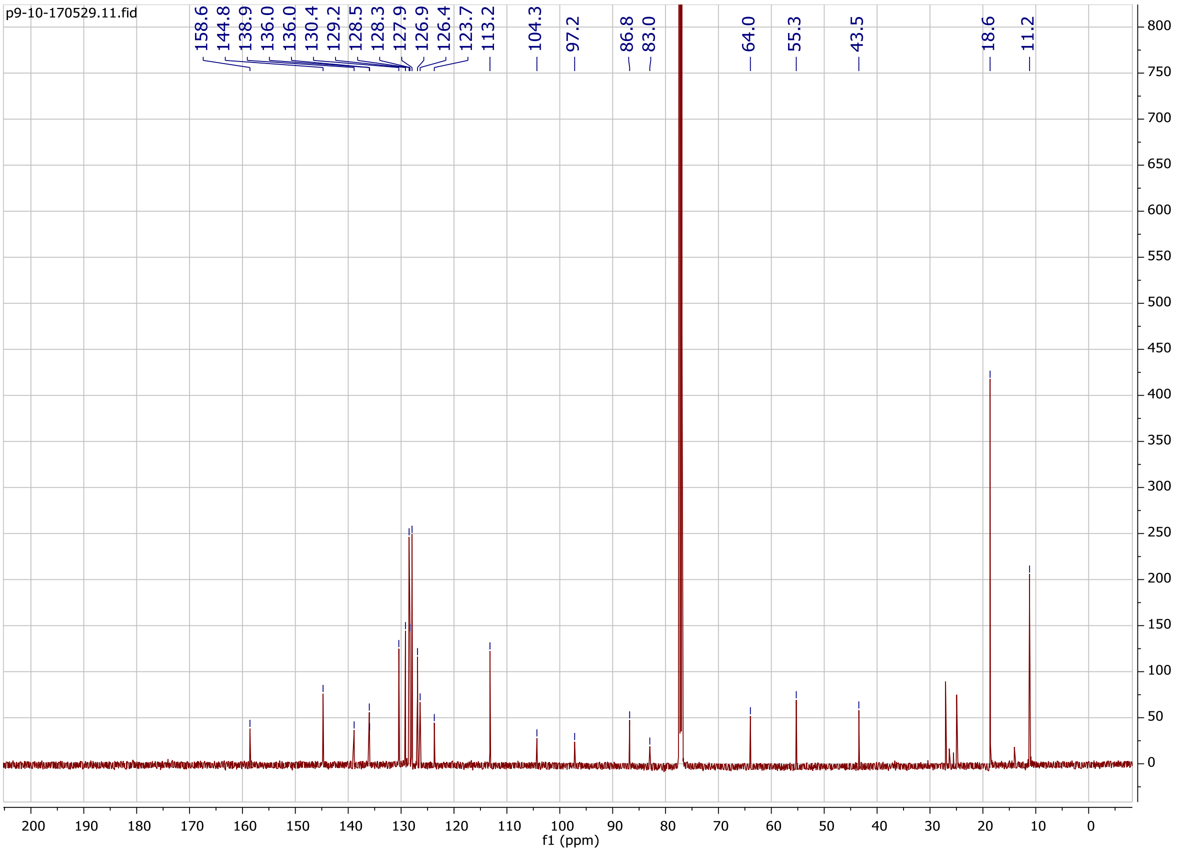

(*E*)-(3-Benzyl-5-((4-methoxyphenyl)diphenylmethoxy)pent-3-en-1-yn-1-yl)triisopropylsilane ((*E*)-7)

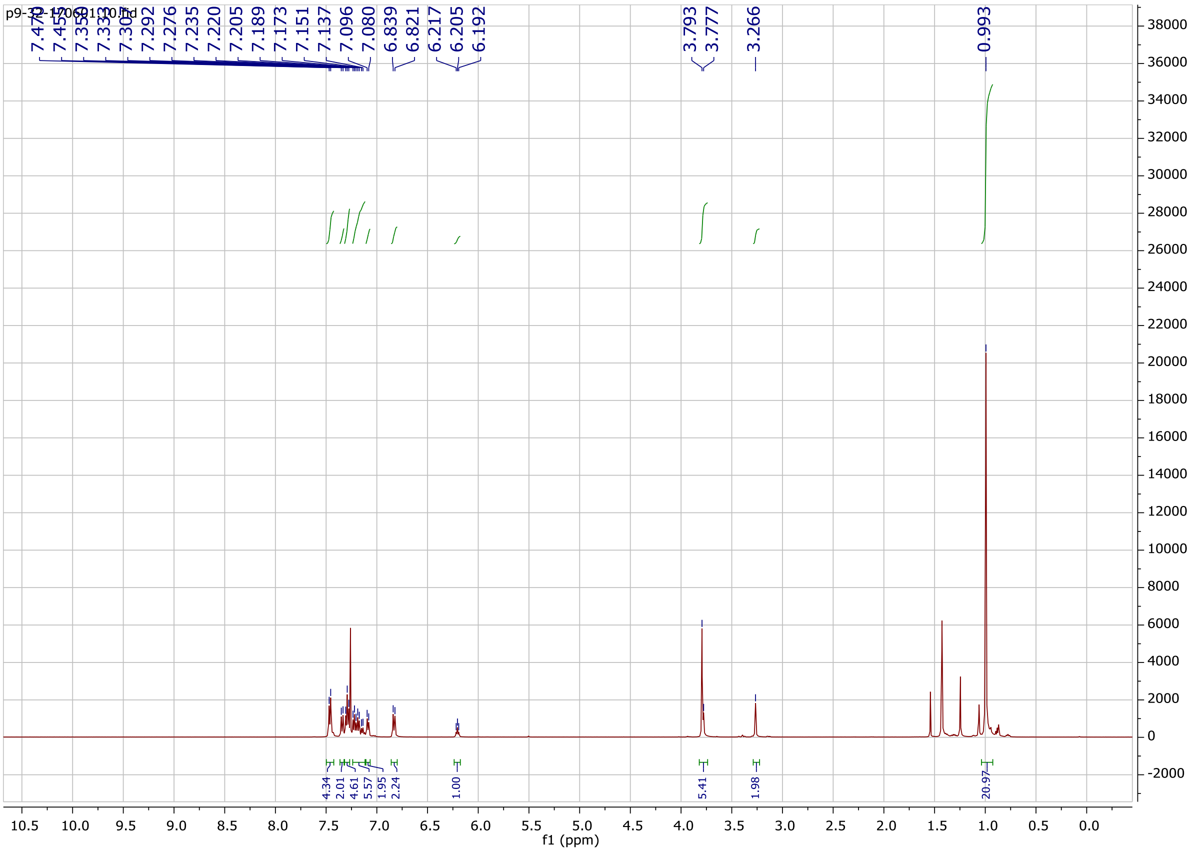

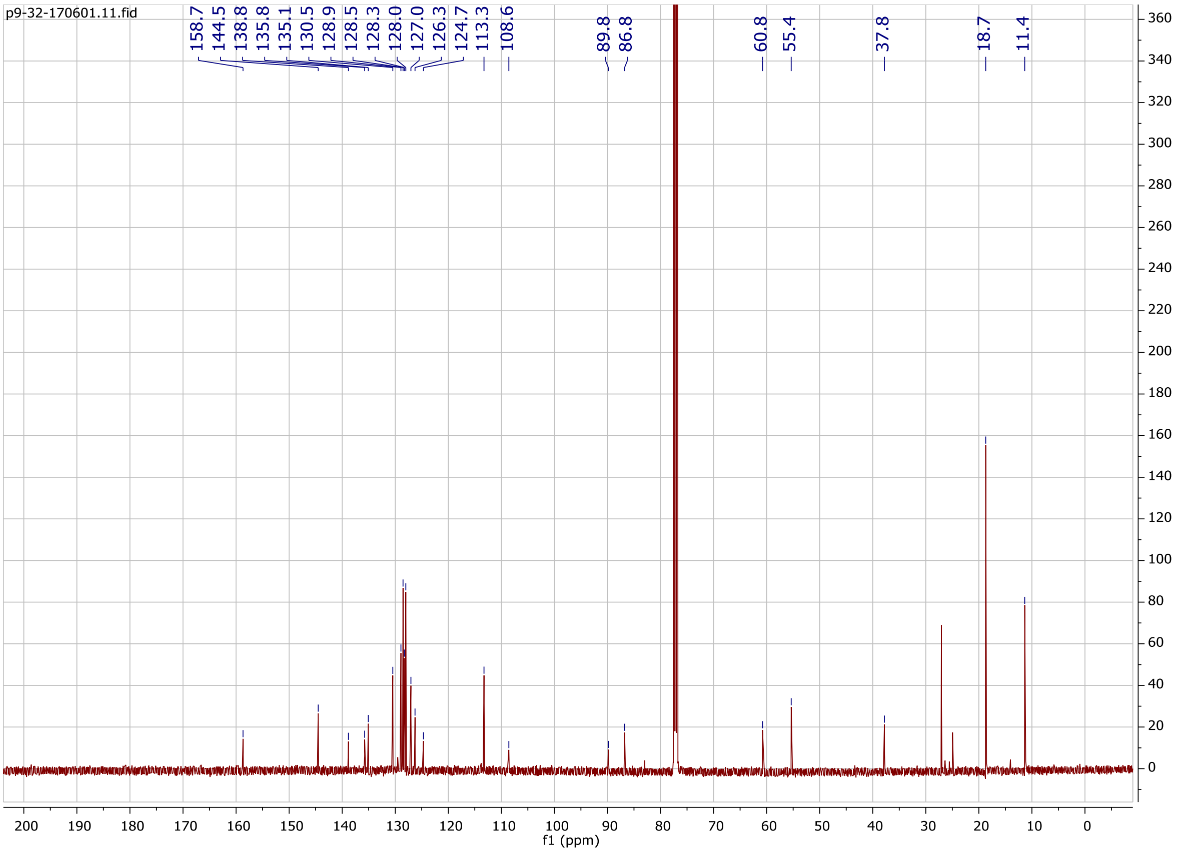

(*Z*)-Triisopropyl(3-(2-((4-methoxyphenyl)diphenylmethoxy)ethylidene)hex-5-en-1-yn-1-yl)silane ((*Z*)-8)

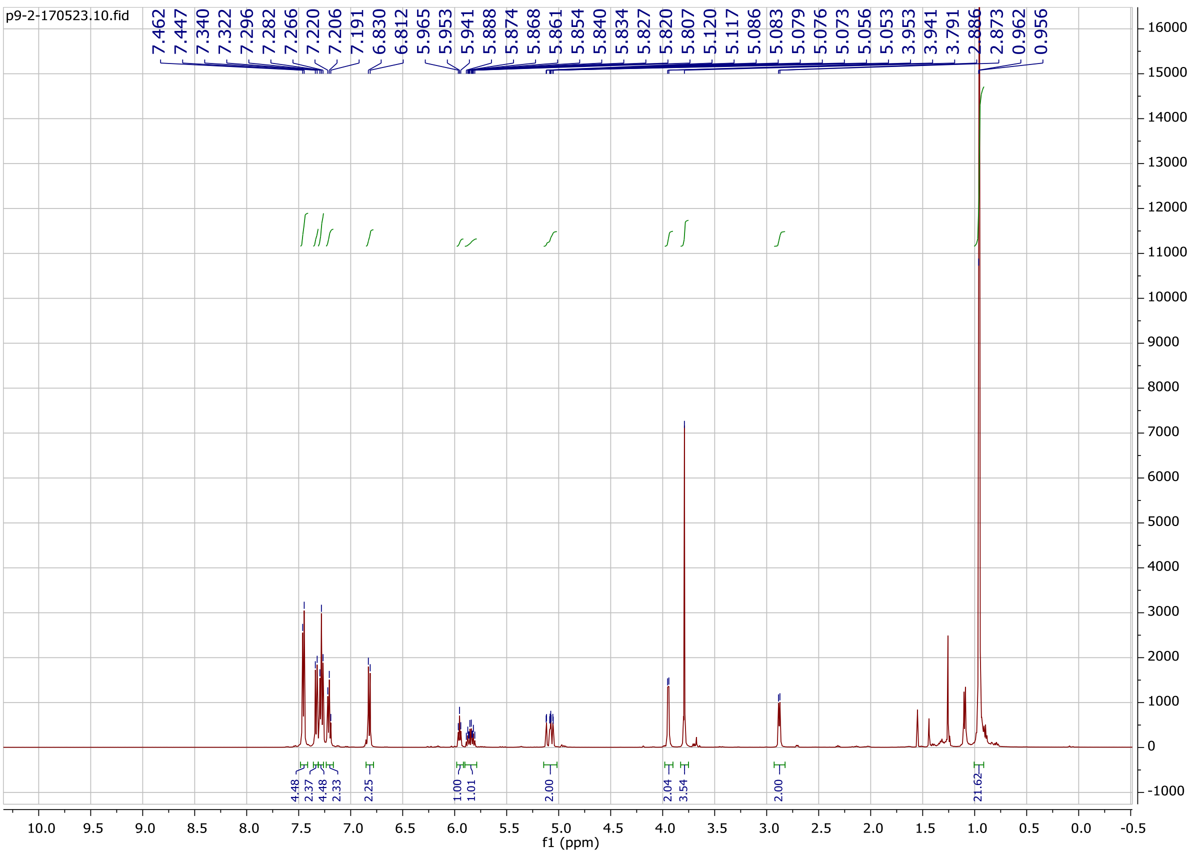

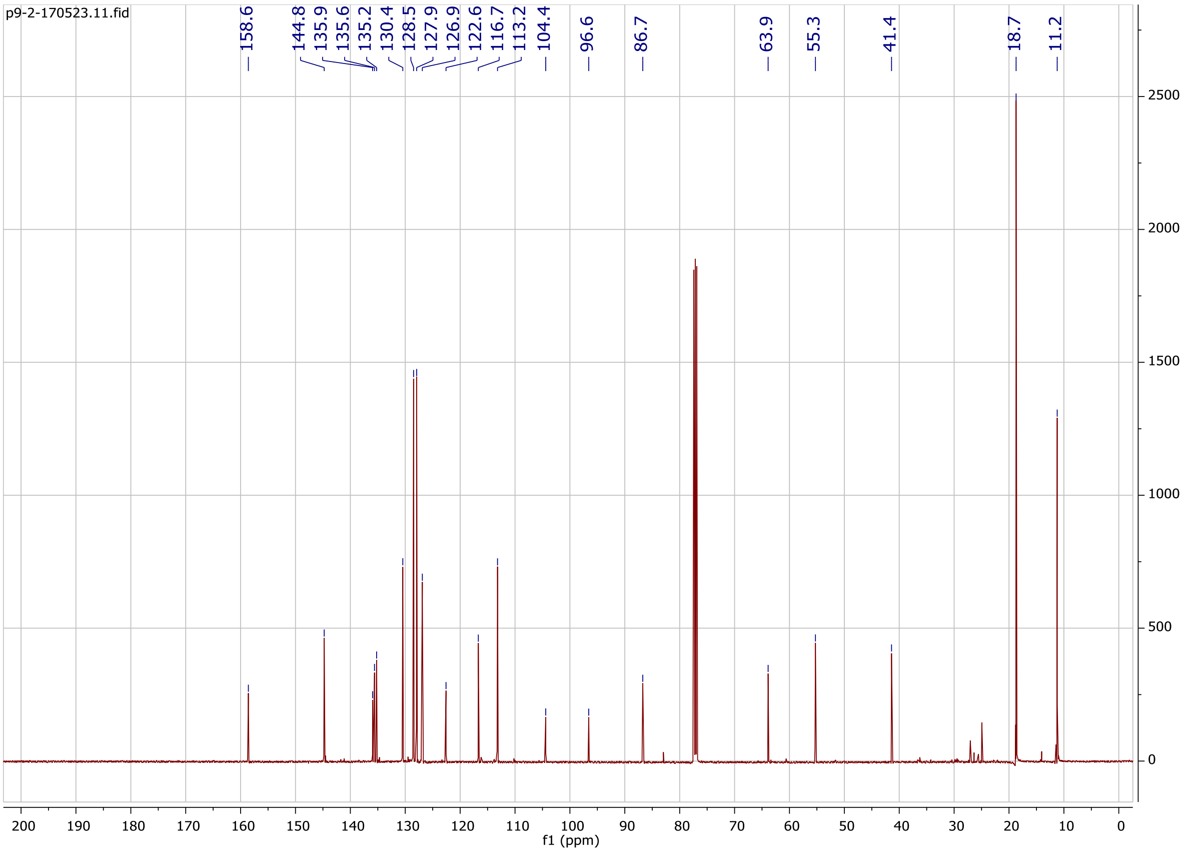

(*E*)-Triisopropyl(3-(2-((4-methoxyphenyl)diphenylmethoxy)ethylidene)hex-5-en-1-yn-1-yl)silane ((*E*)-8)

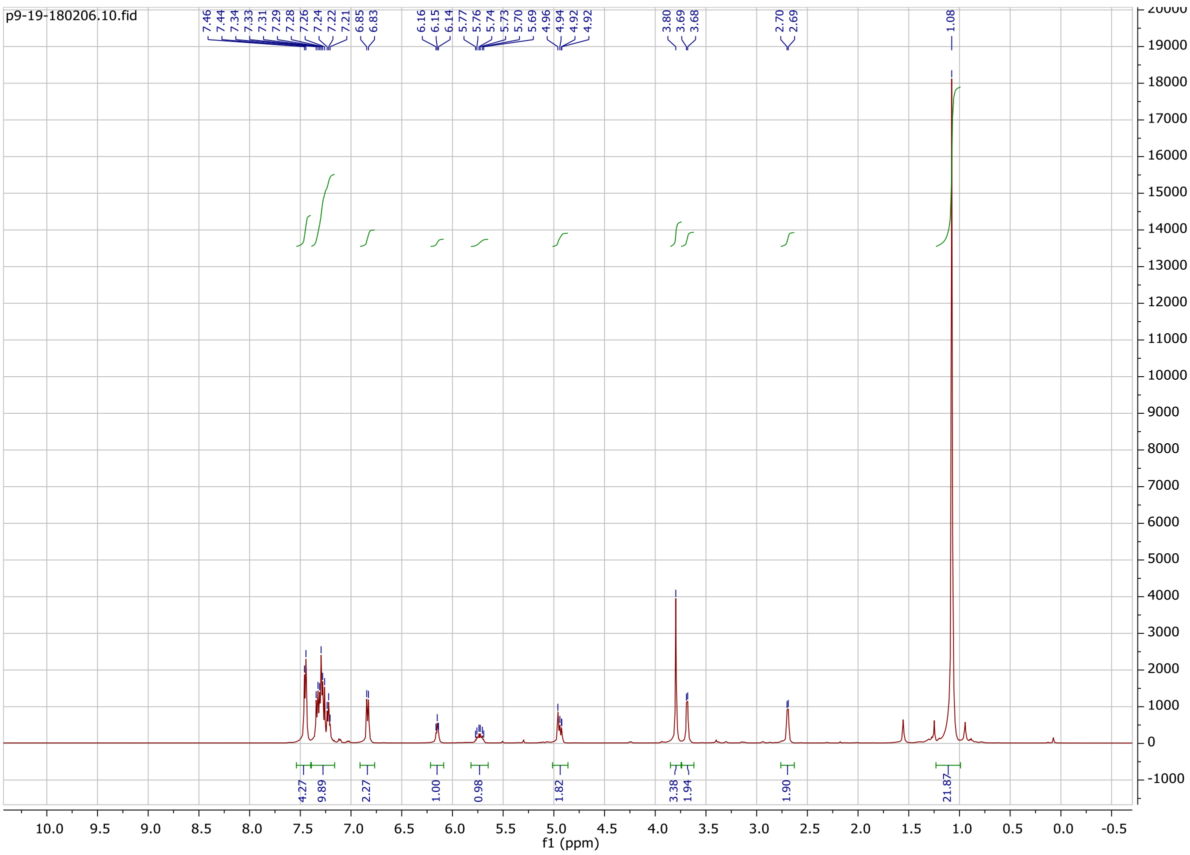

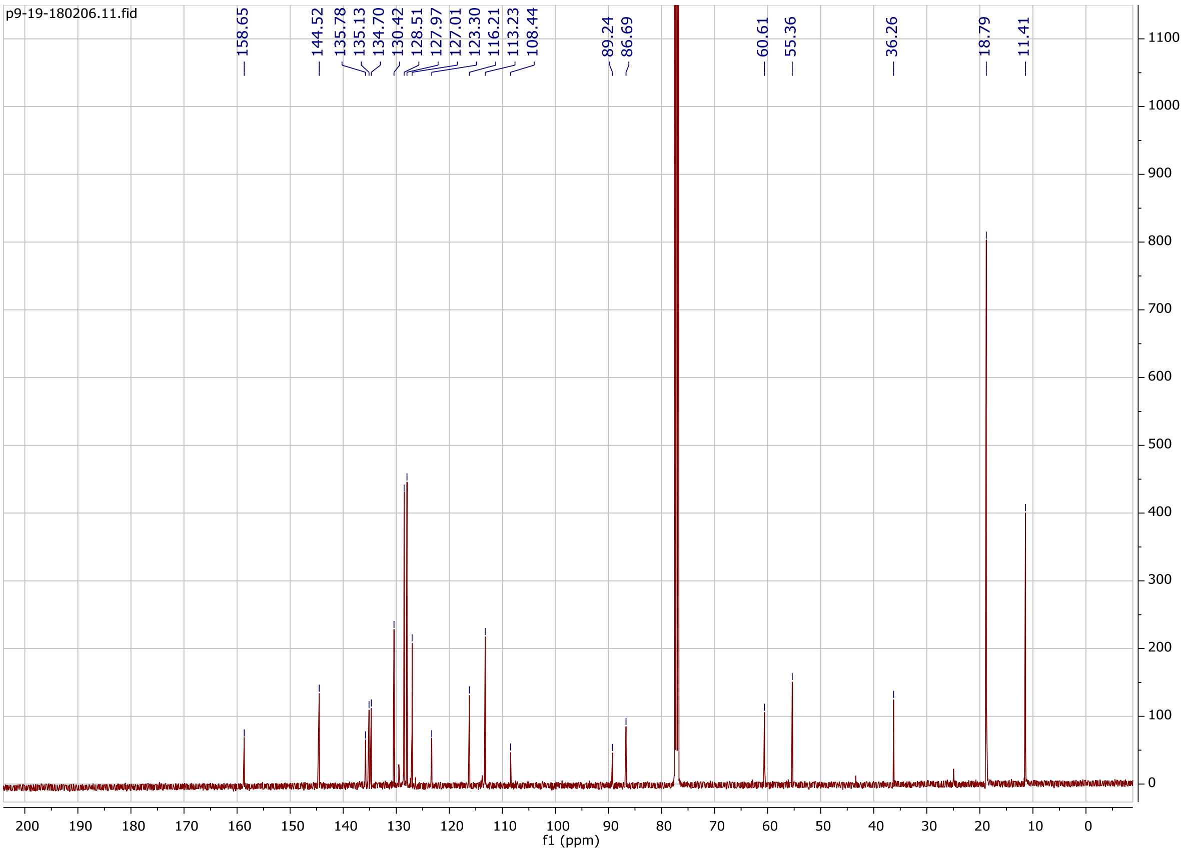

(*Z*)-Triisopropyl(3-(2-((4-methoxyphenyl)diphenylmethoxy)ethylidene)hept-1-yn-1-yl)silane ((*Z*)-9)

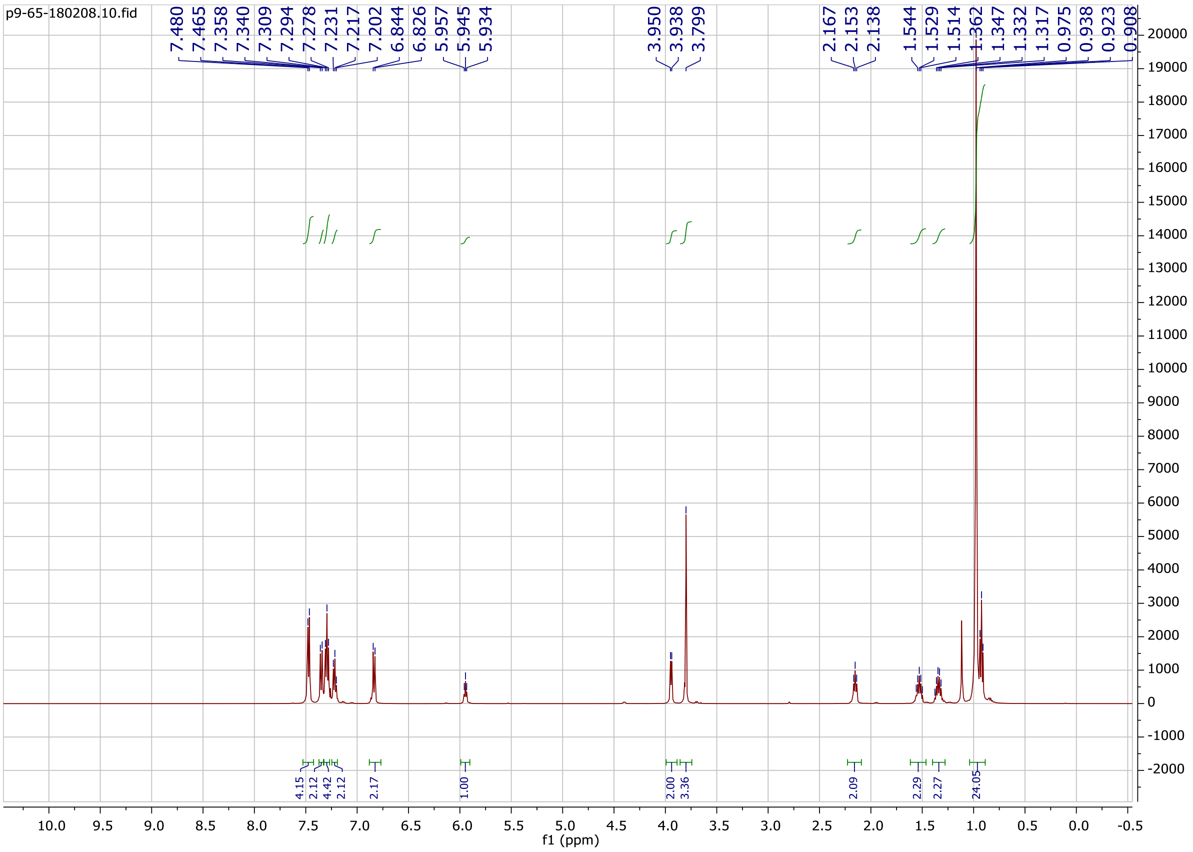

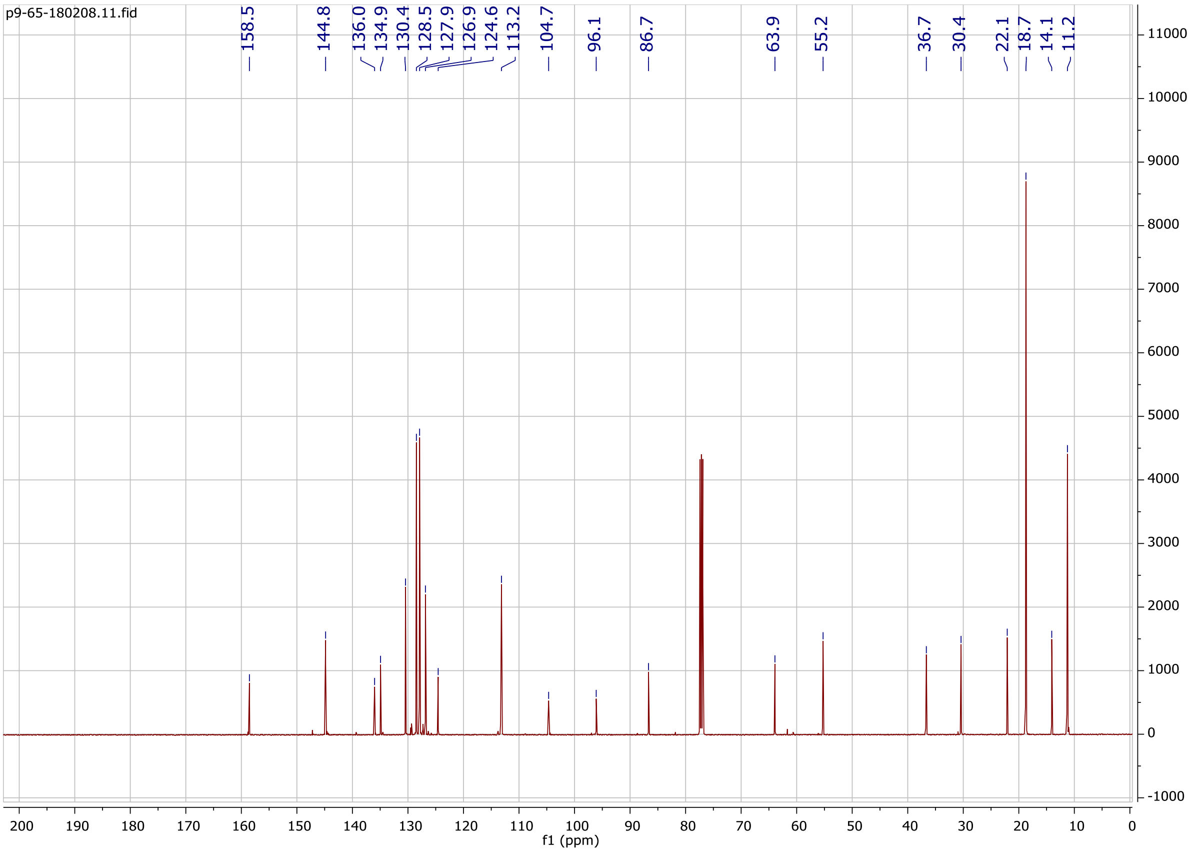

(*E*)-Triisopropyl(3-(2-((4-methoxyphenyl)diphenylmethoxy)ethylidene)hept-1-yn-1-yl)silane ((*E*)-9)

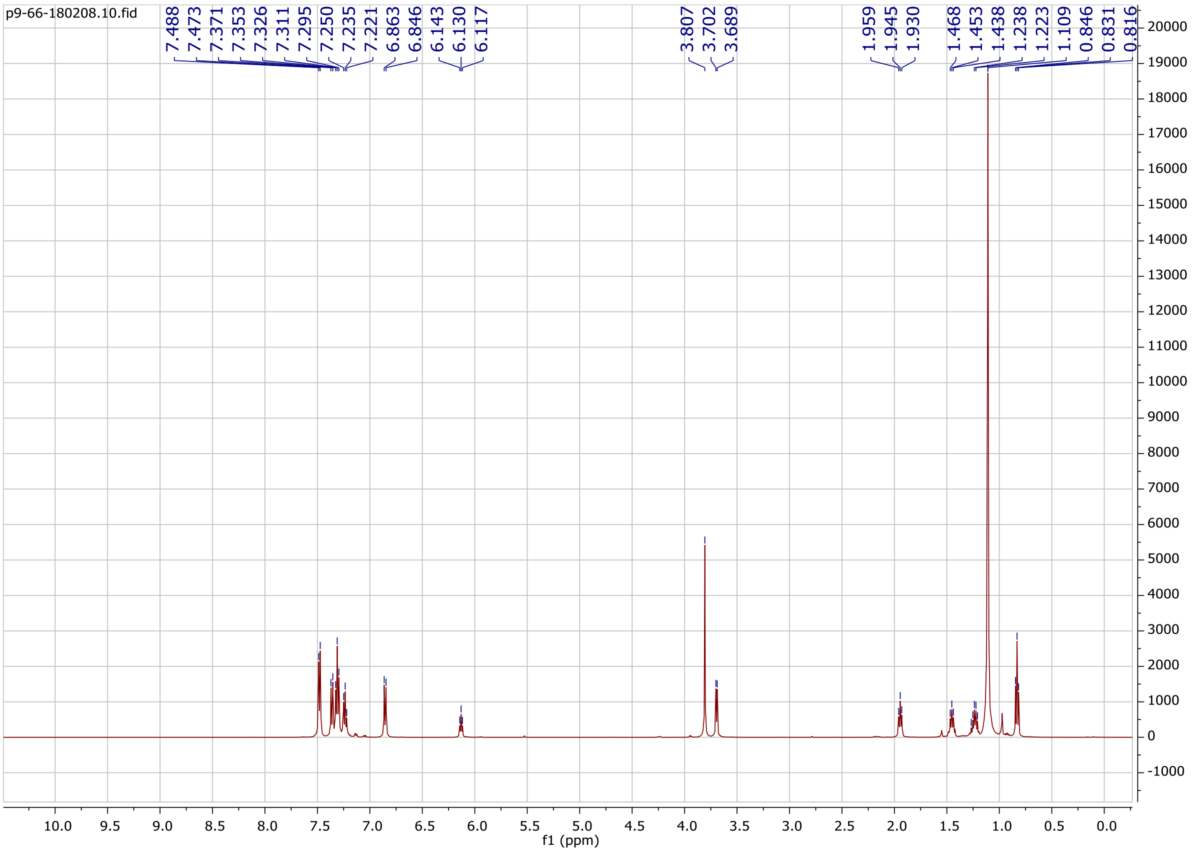

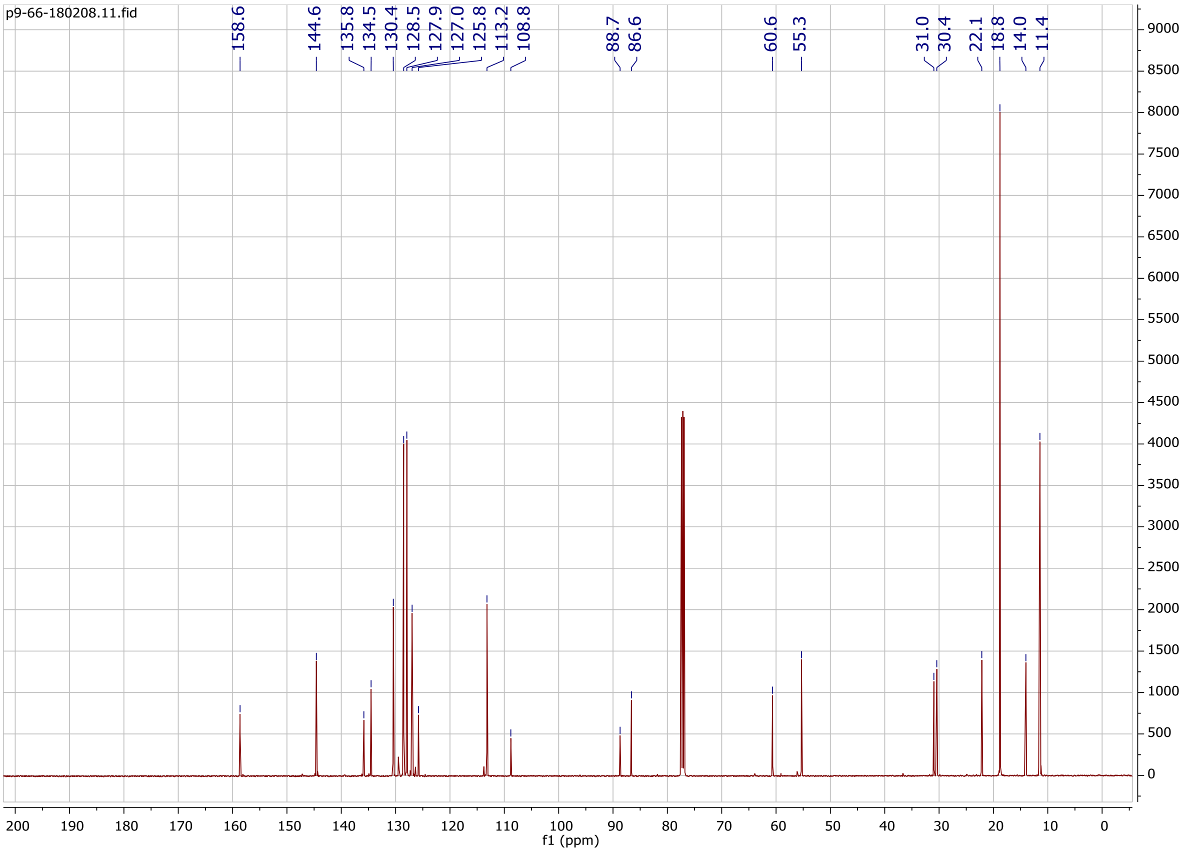

trans-2-Ethynyl-3-phenyloxirane (M)

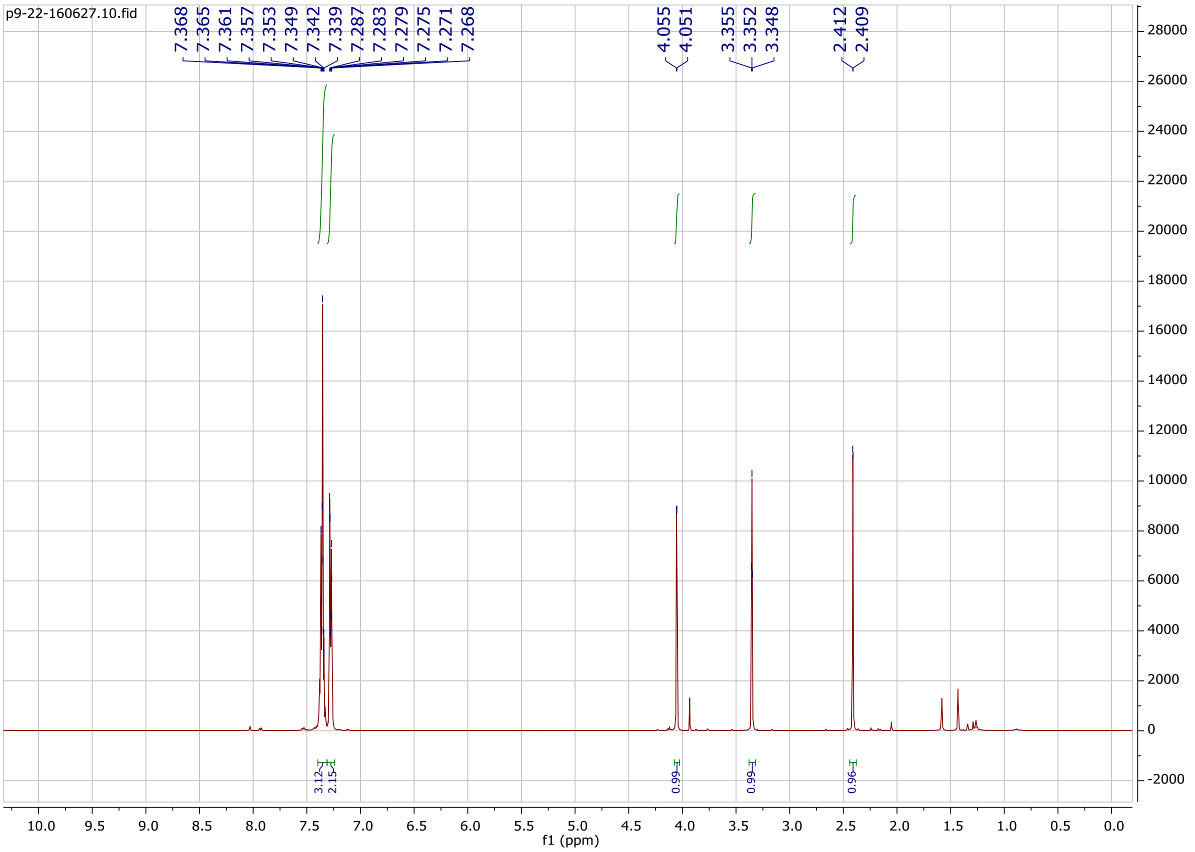

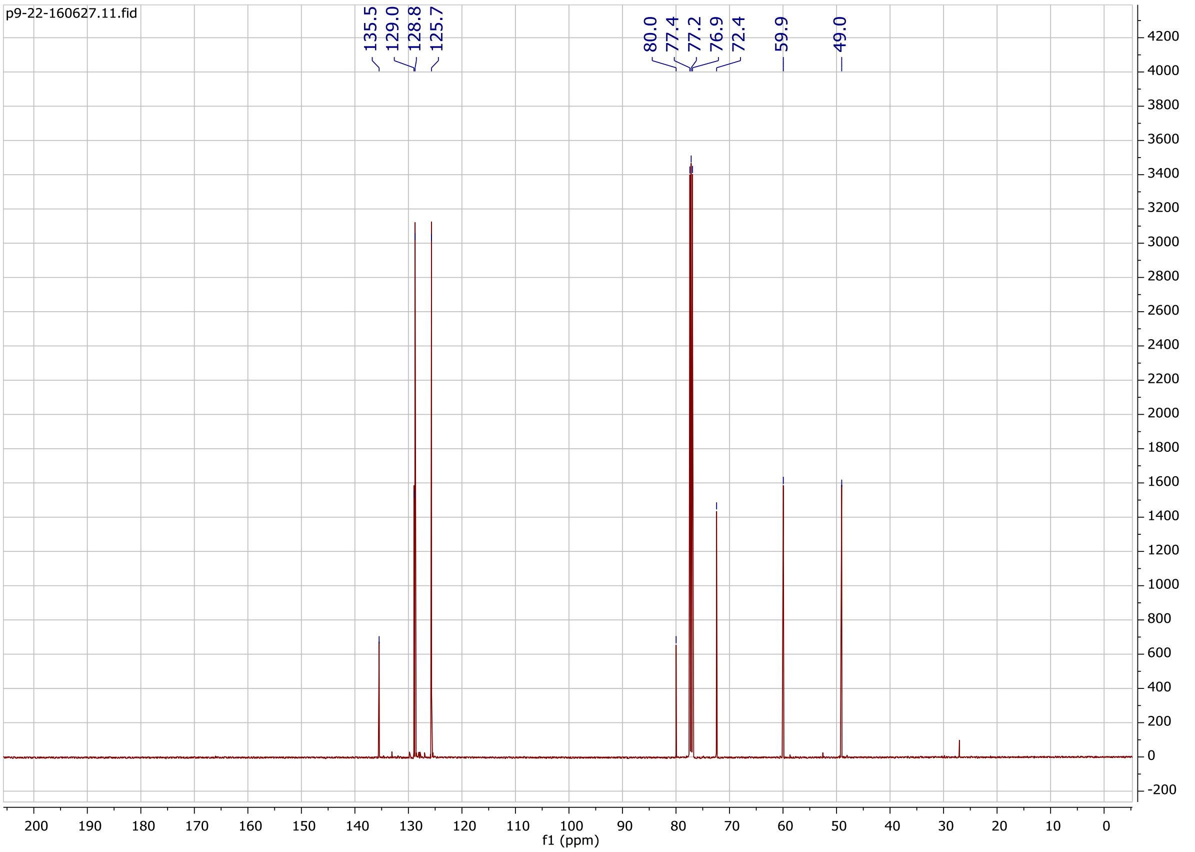

***trans*-Triisopropyl((3-phenyloxiran-2-yl)ethynyl)silane (*trans*-1d)**

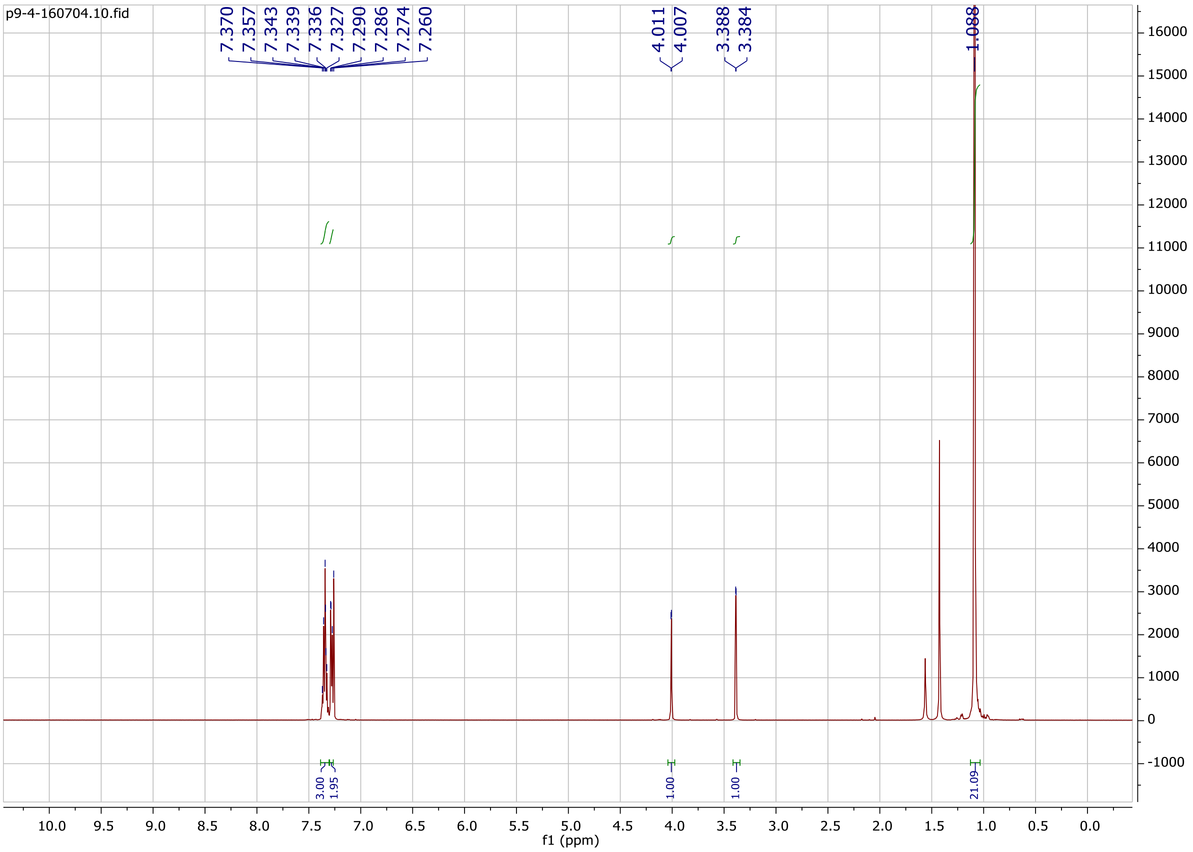

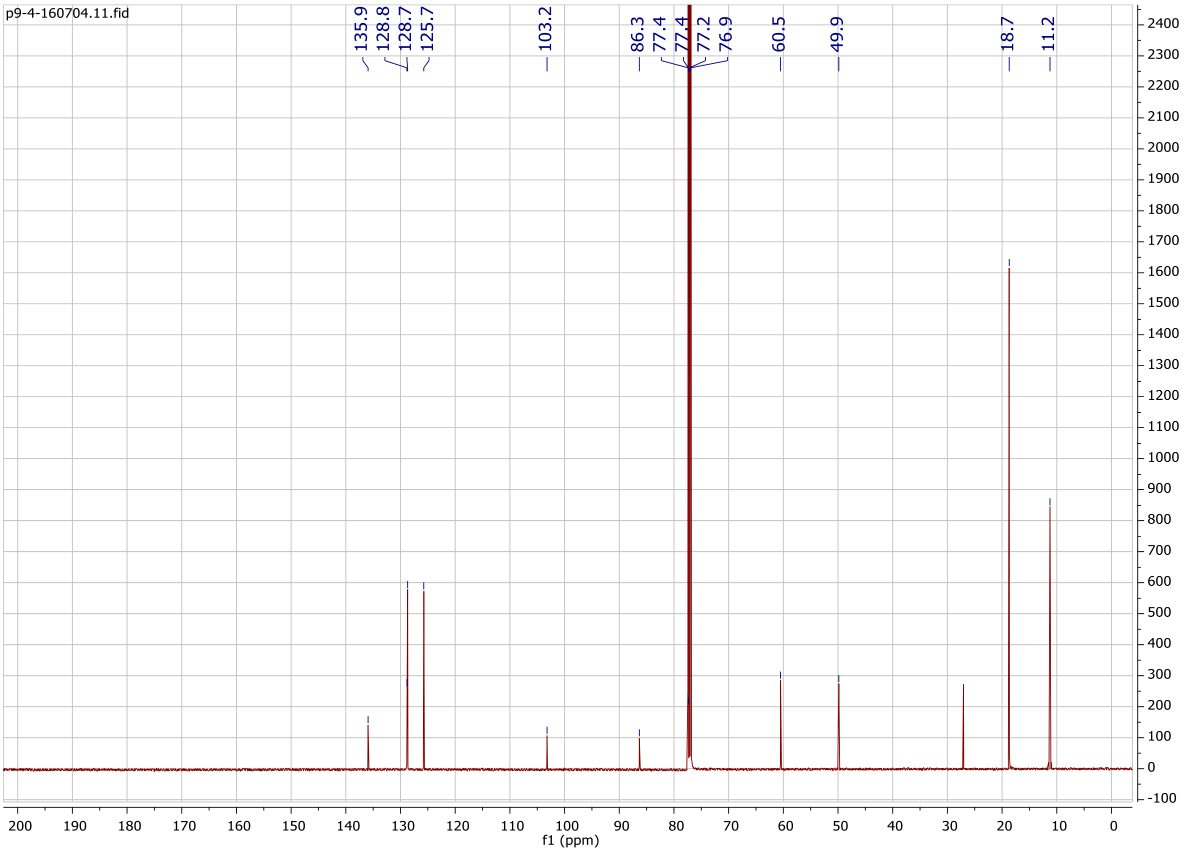

***cis*-2-Ethynyl-3-phenyloxirane (O)**

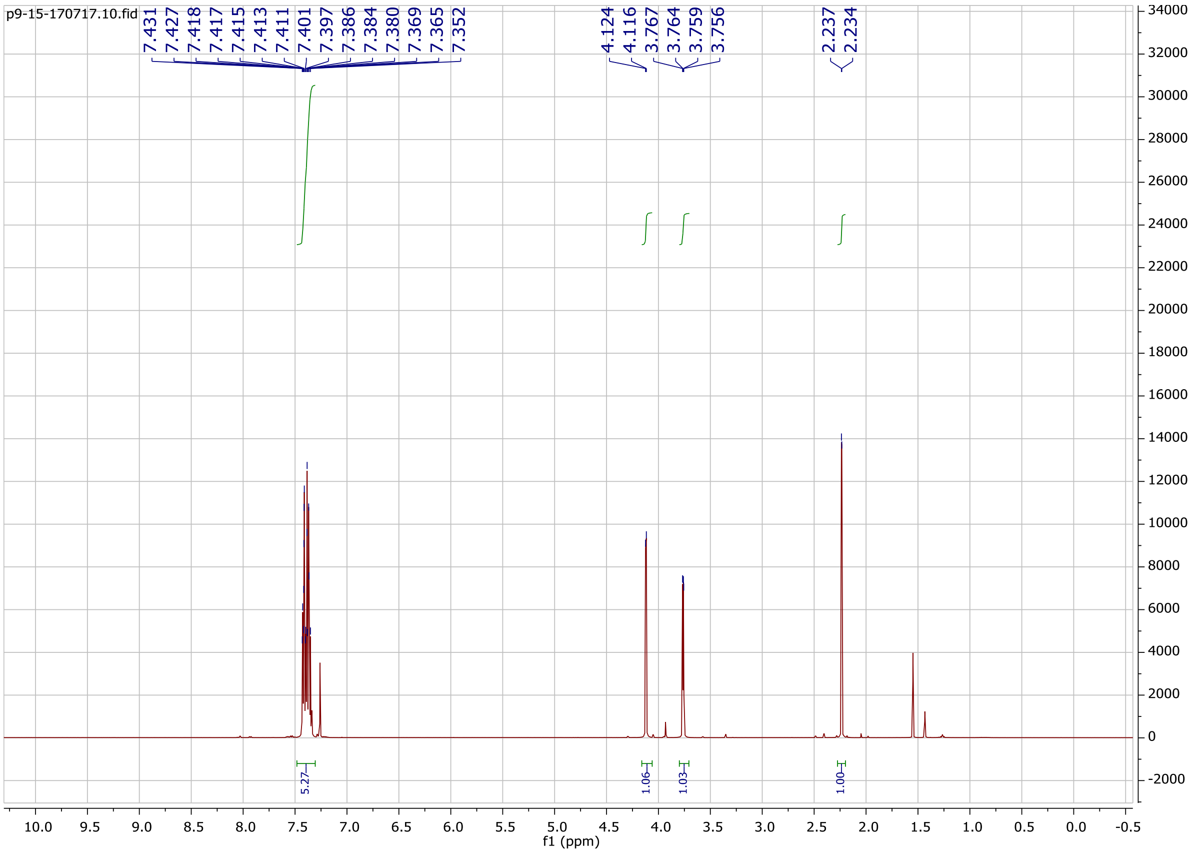

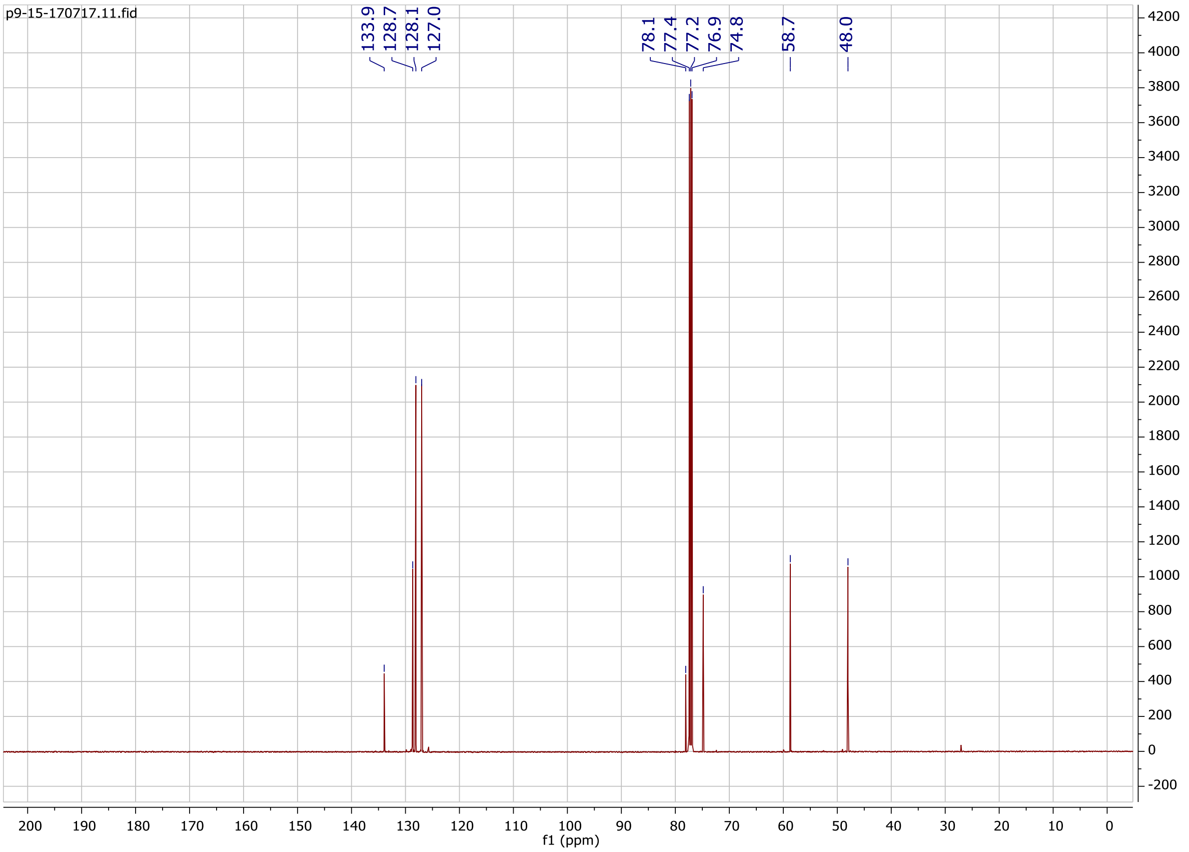

cis-Triisopropyl((3-phenyloxiran-2-yl)ethynyl)silane (*cis*-1d)

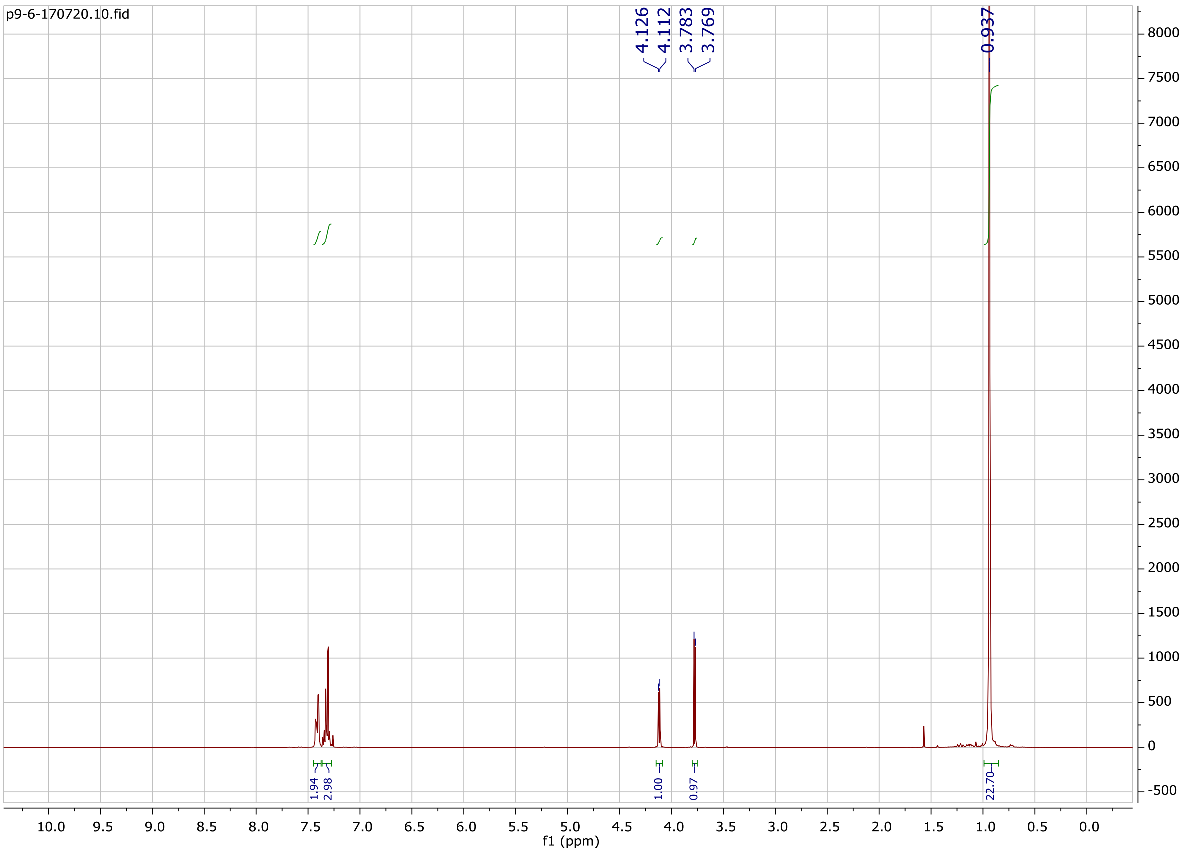

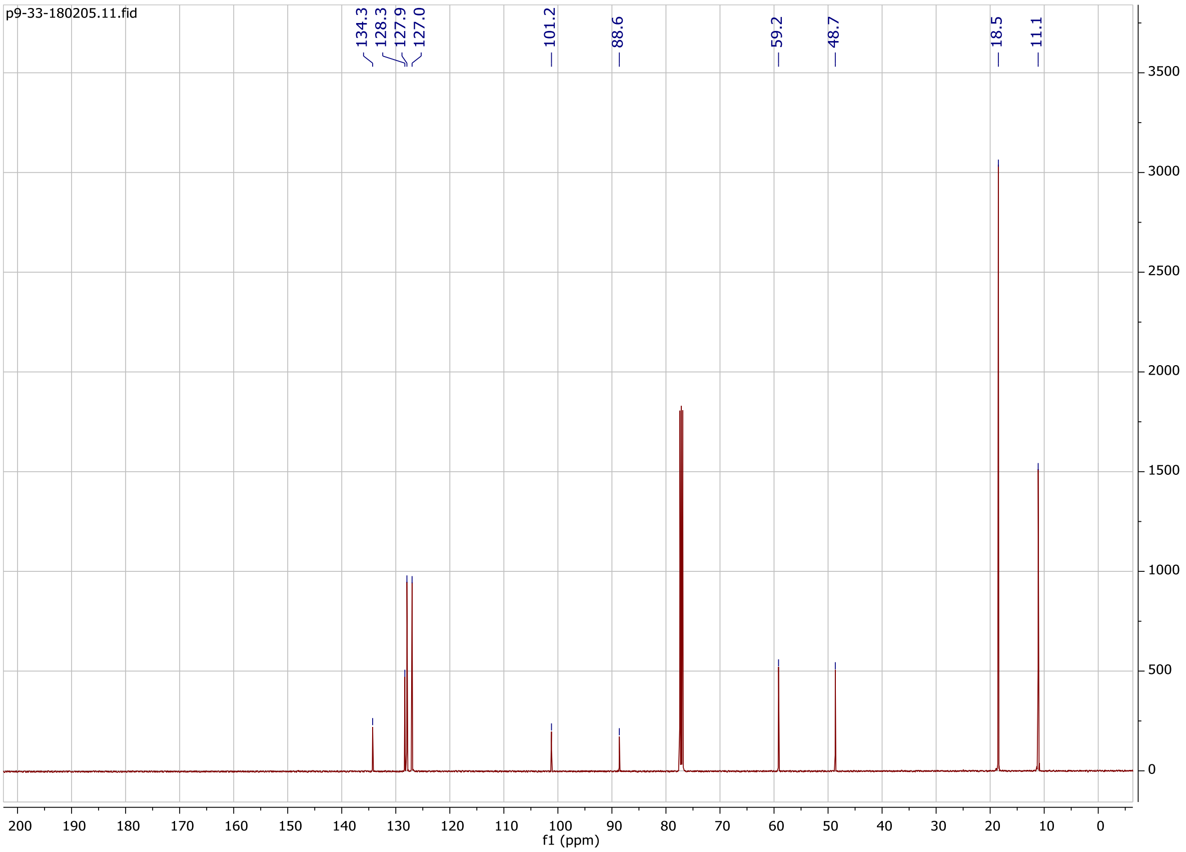

cis-2-Ethynyl-3-hexyloxirane (Q)

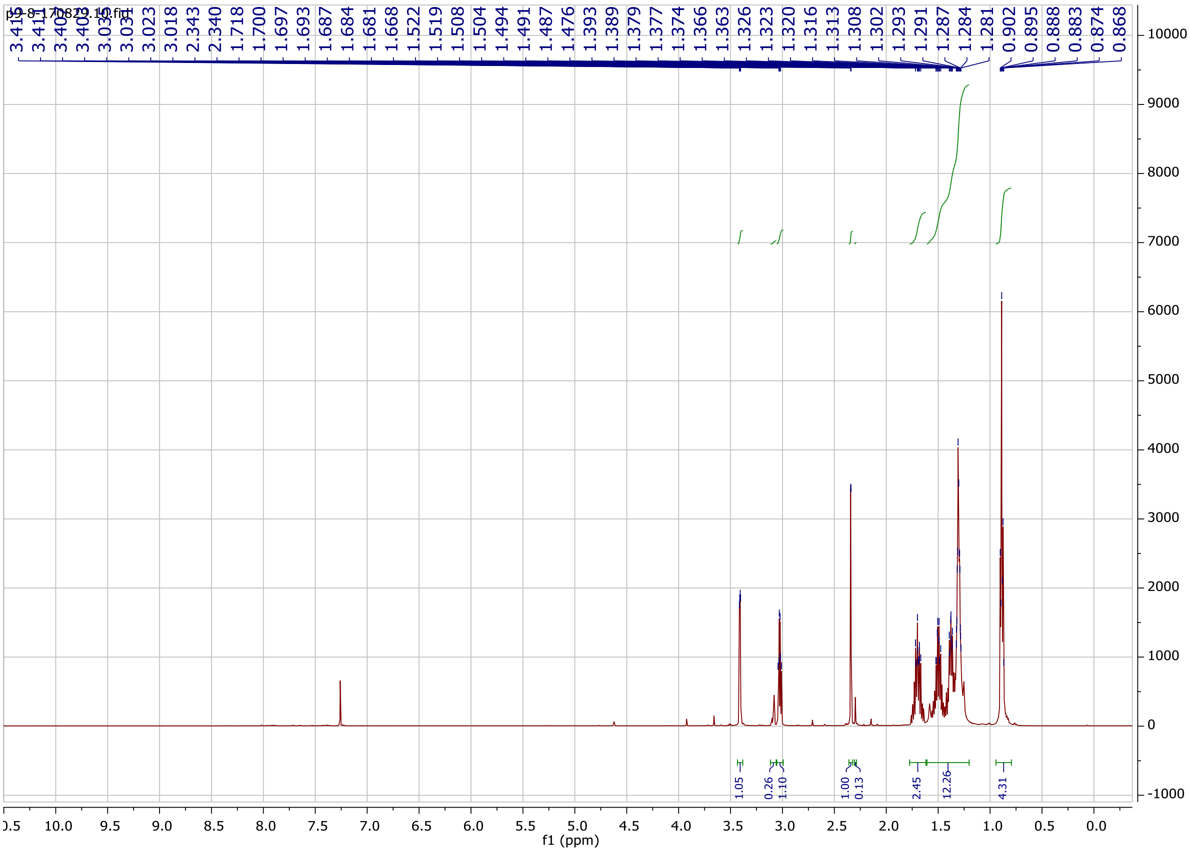

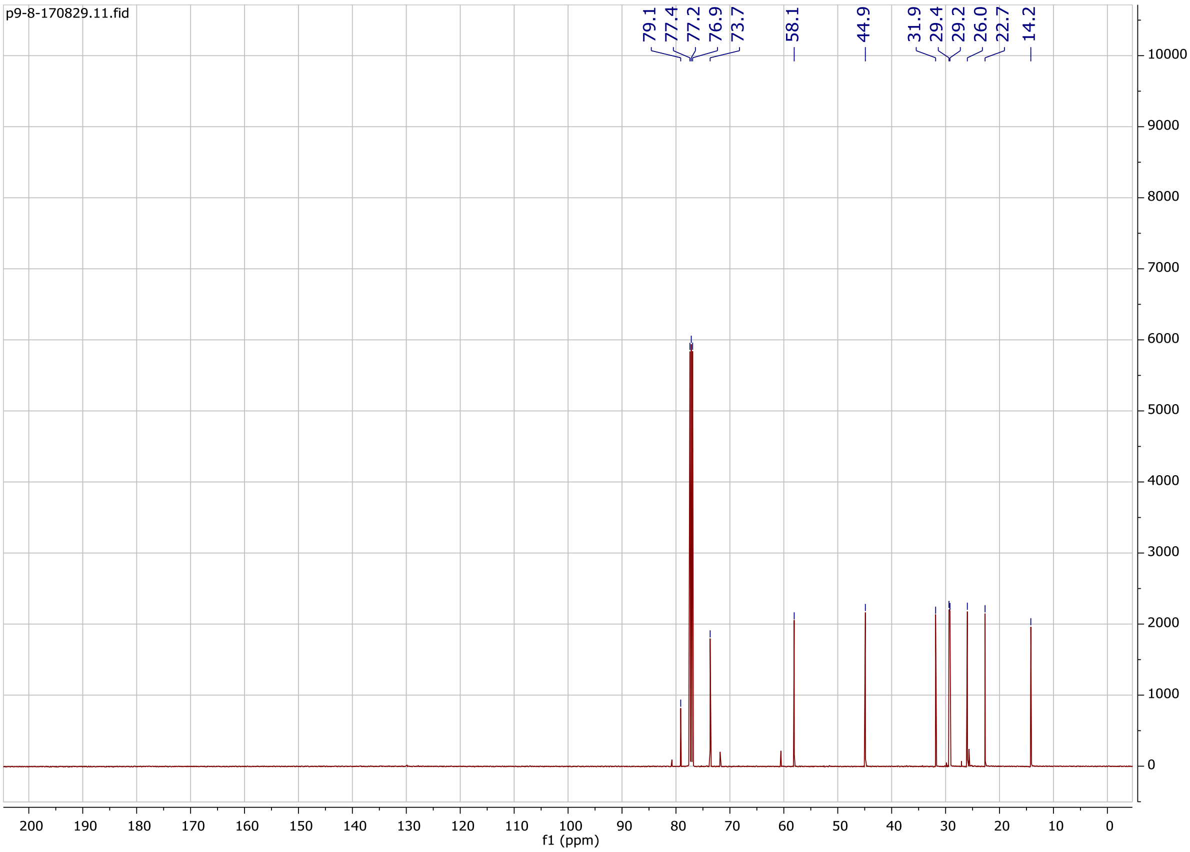

cis-Triisopropyl((3-hexyloxiran-2-yl)ethynyl)silane (*cis*-1c)

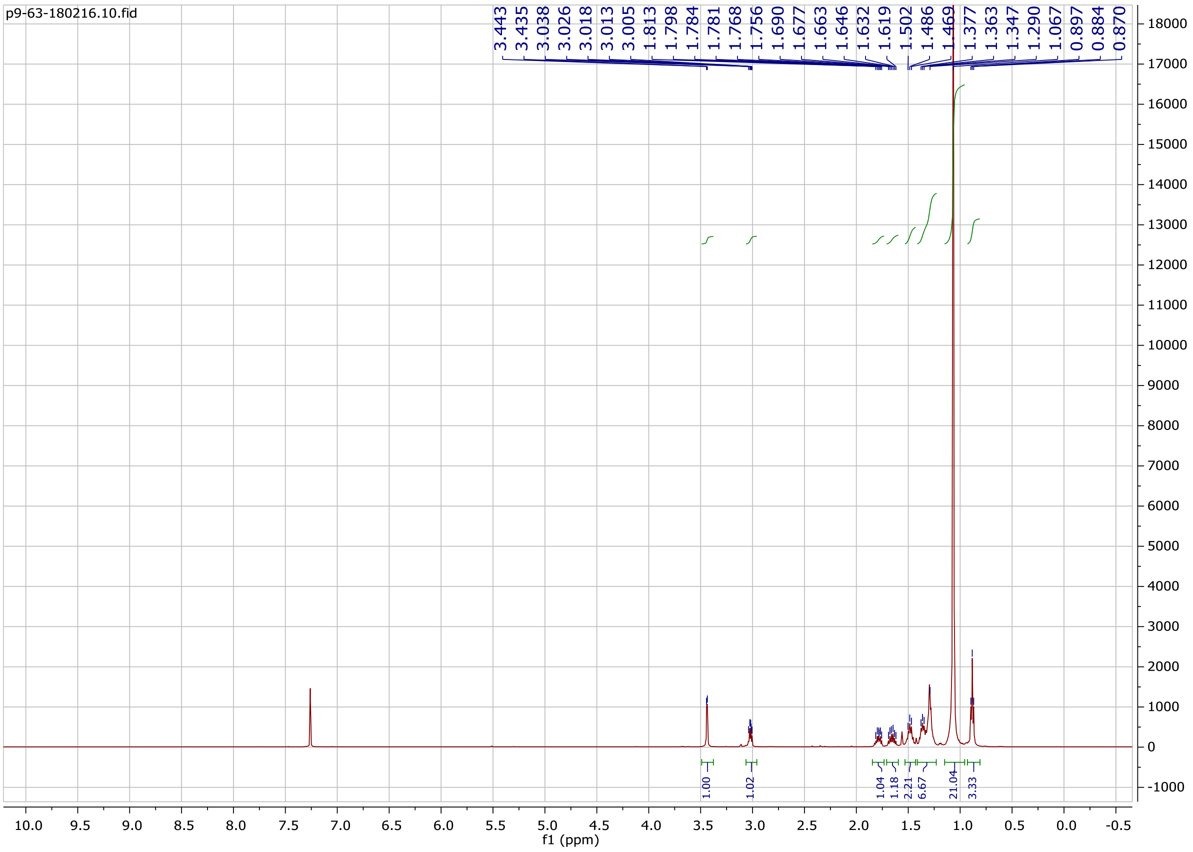

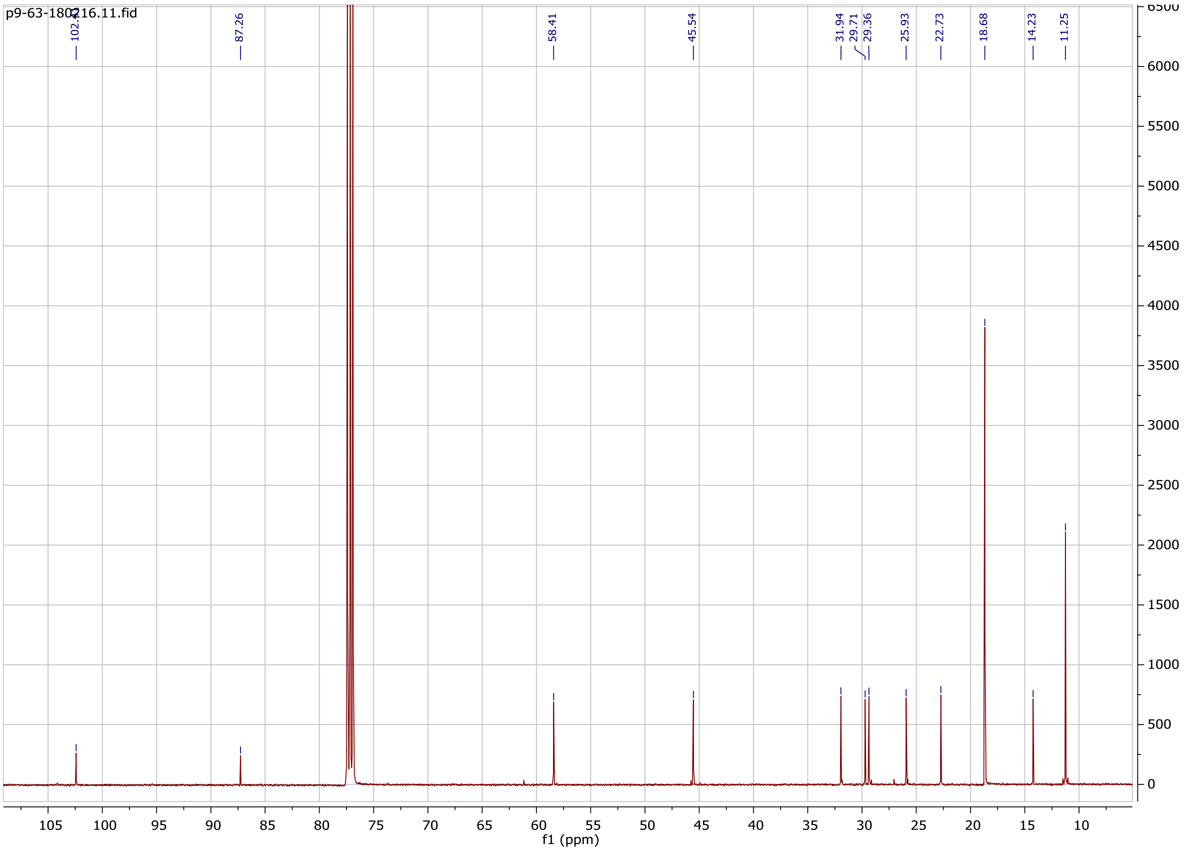

trans-2-Ethynyl-3-hexyloxirane (S)

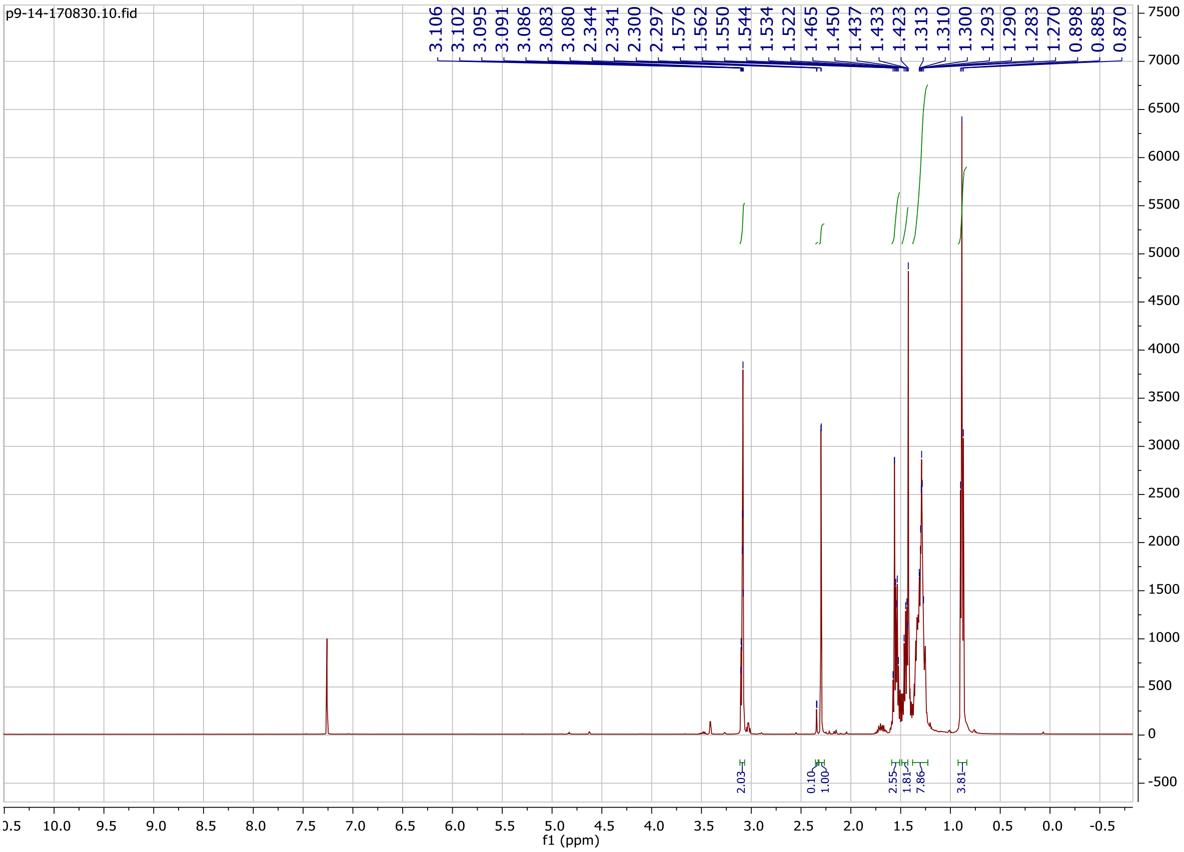

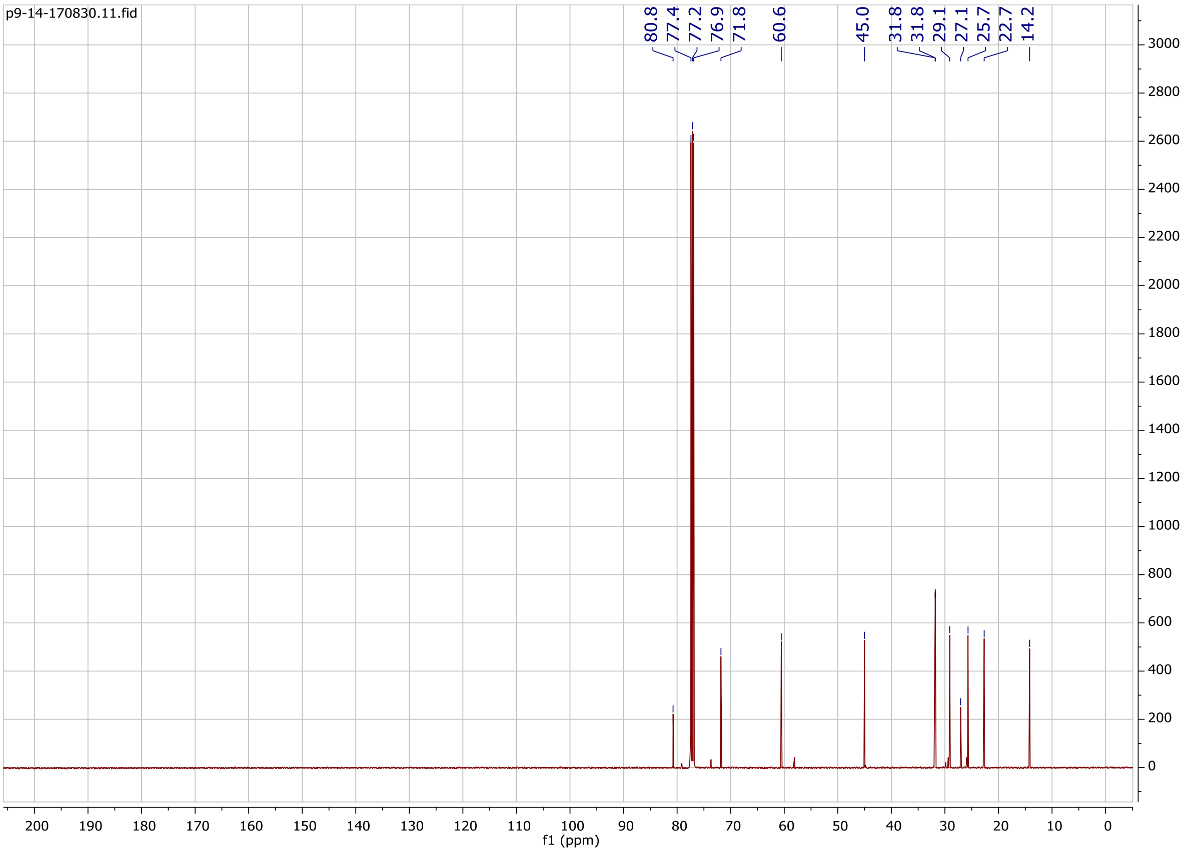

trans-Triisopropyl((3-hexyloxiran-2-yl)ethynyl)silane (_trans_-1c)

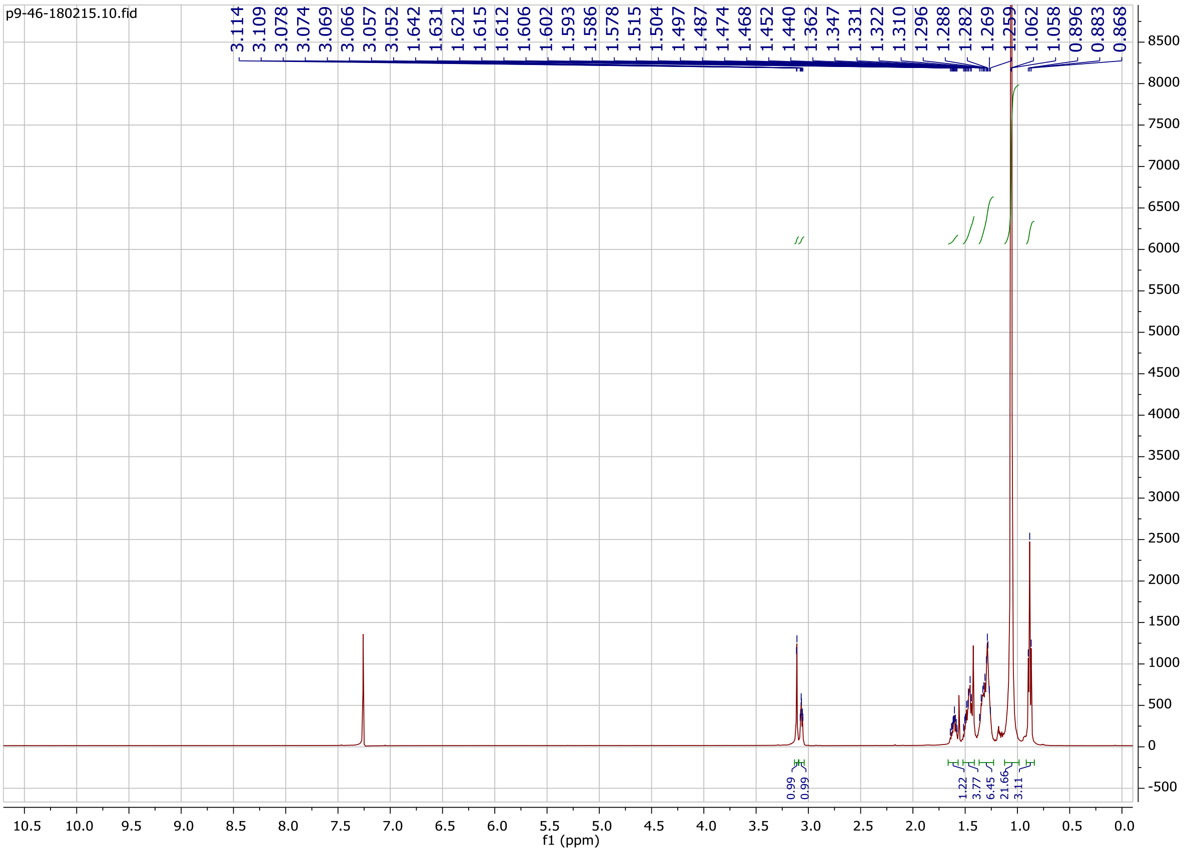

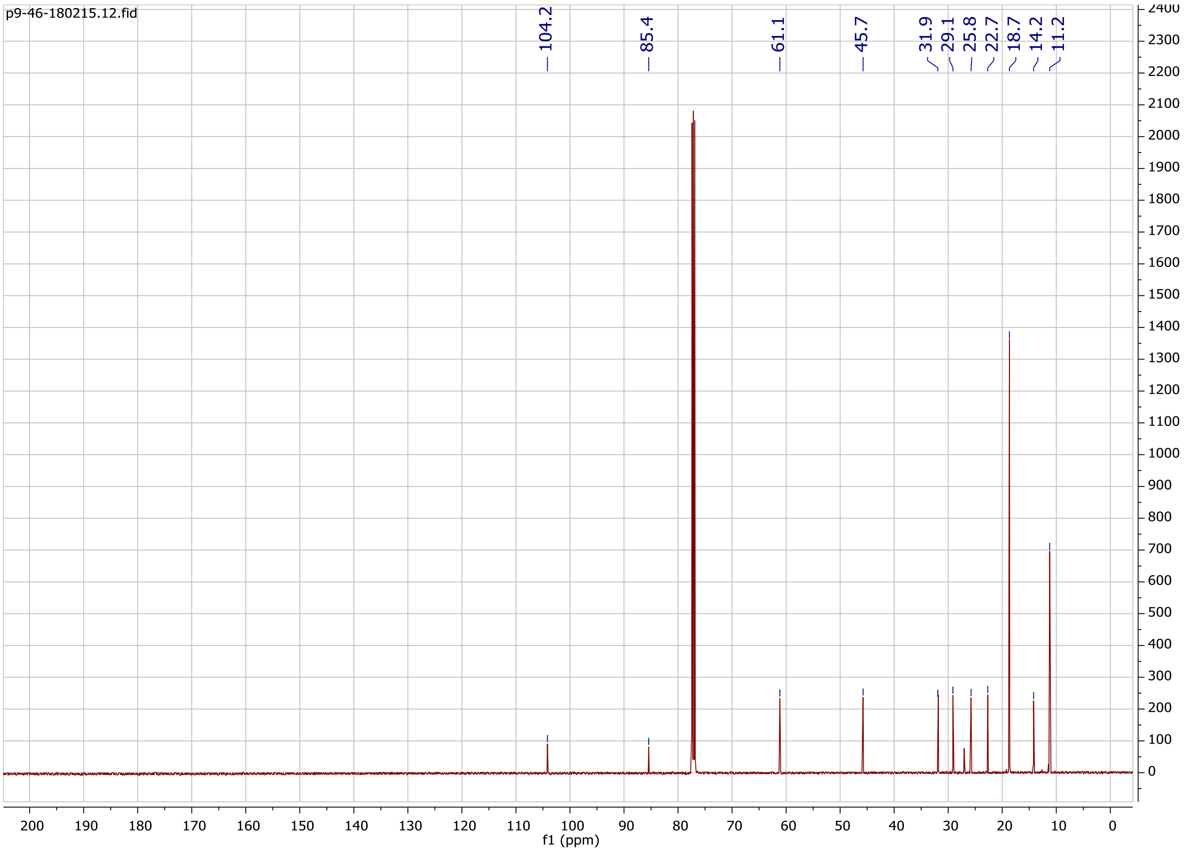

cis-((3-Ethynyloxiran-2-yl)ethynyl)triisopropylsilane (T)

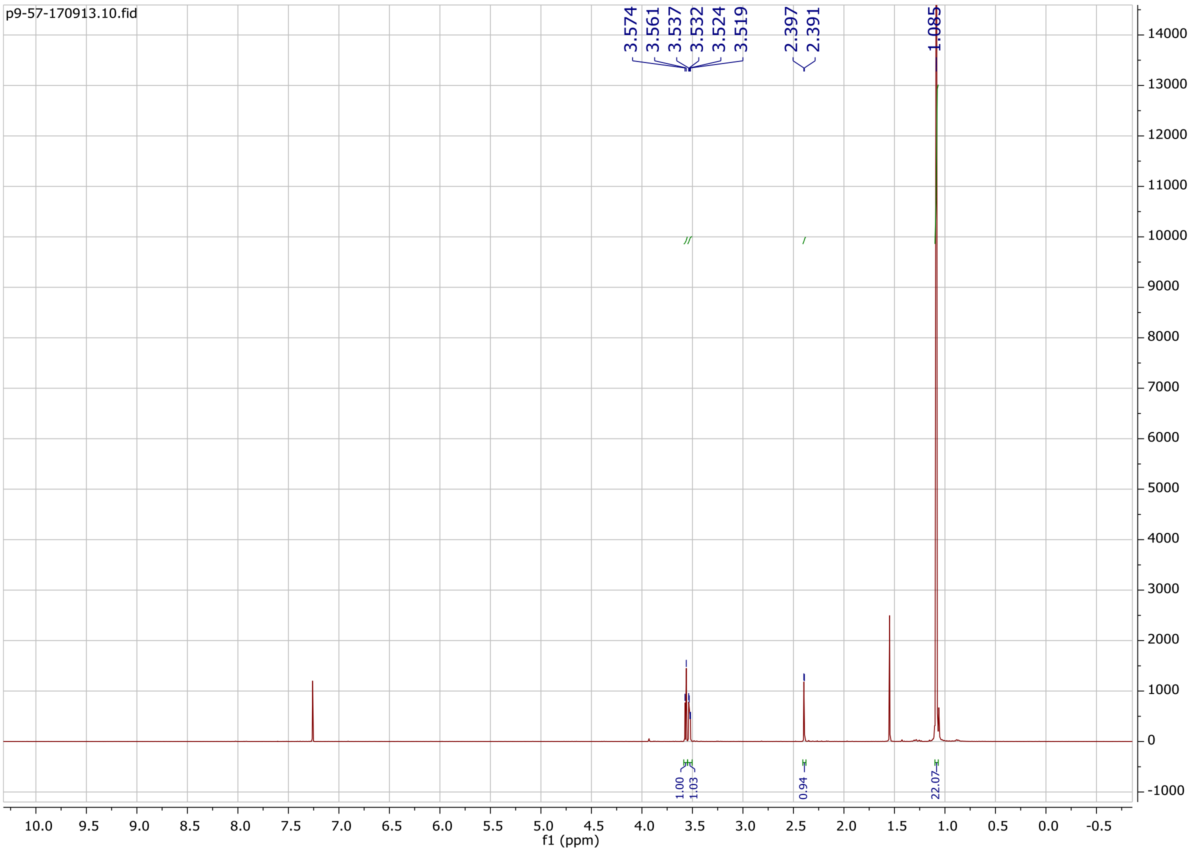

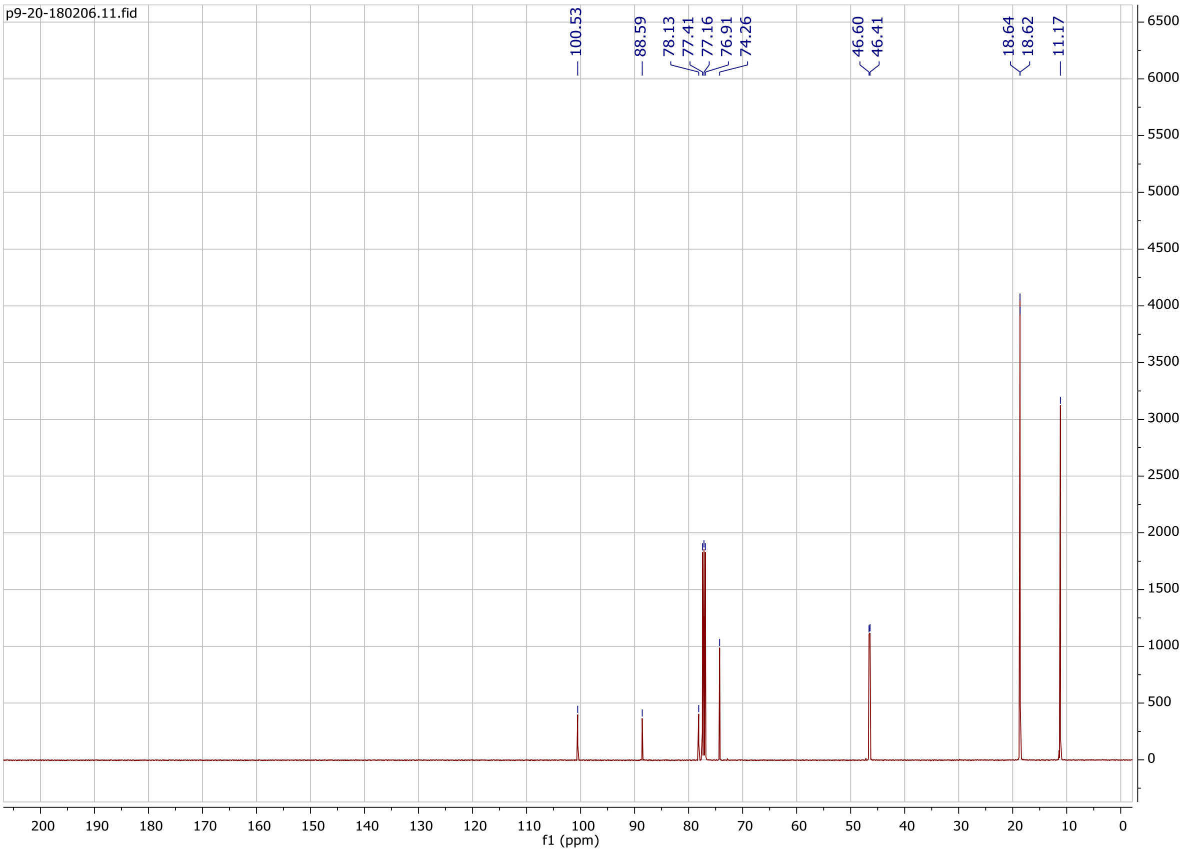

***cis*-2,3-Bis((triisopropylsilyl)ethynyl)oxirane (*cis*-1e)**

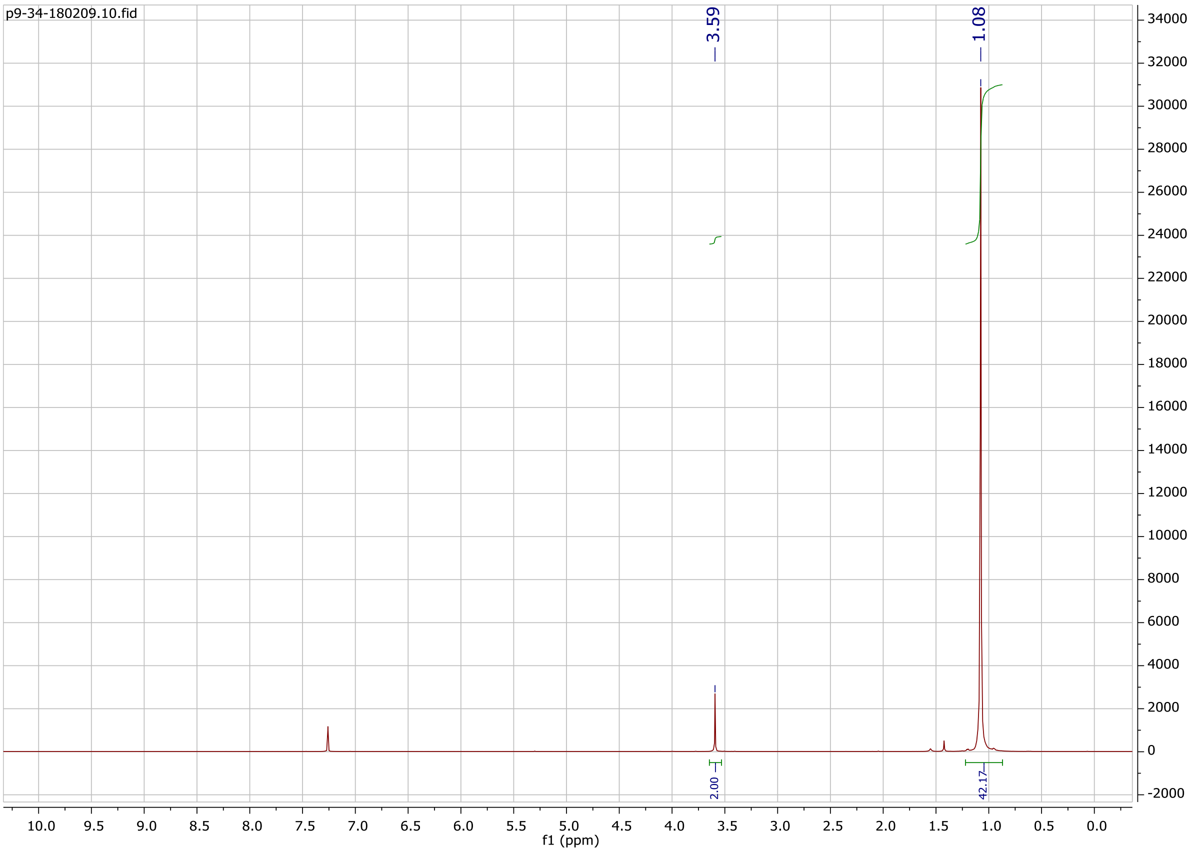

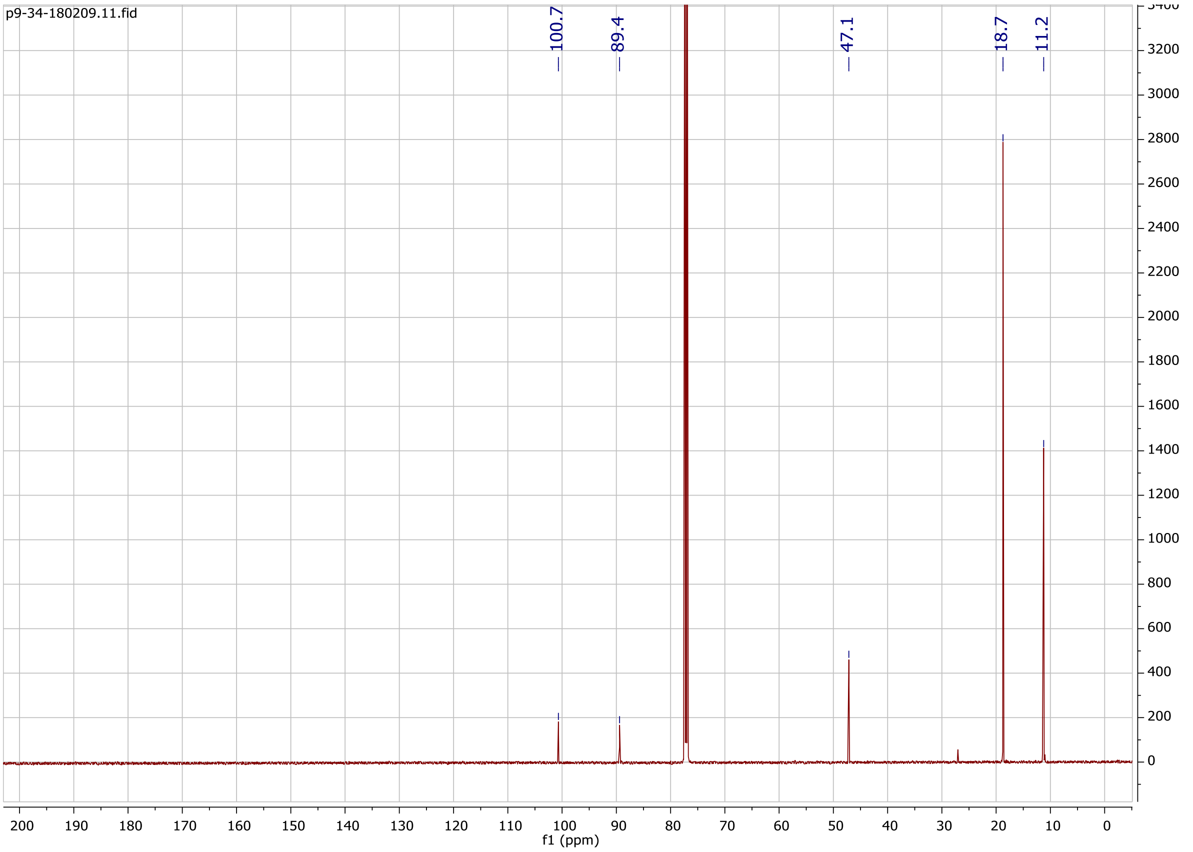

trans-((3-Ethynyloxiran-2-yl)ethynyl)triisopropylsilane (U)

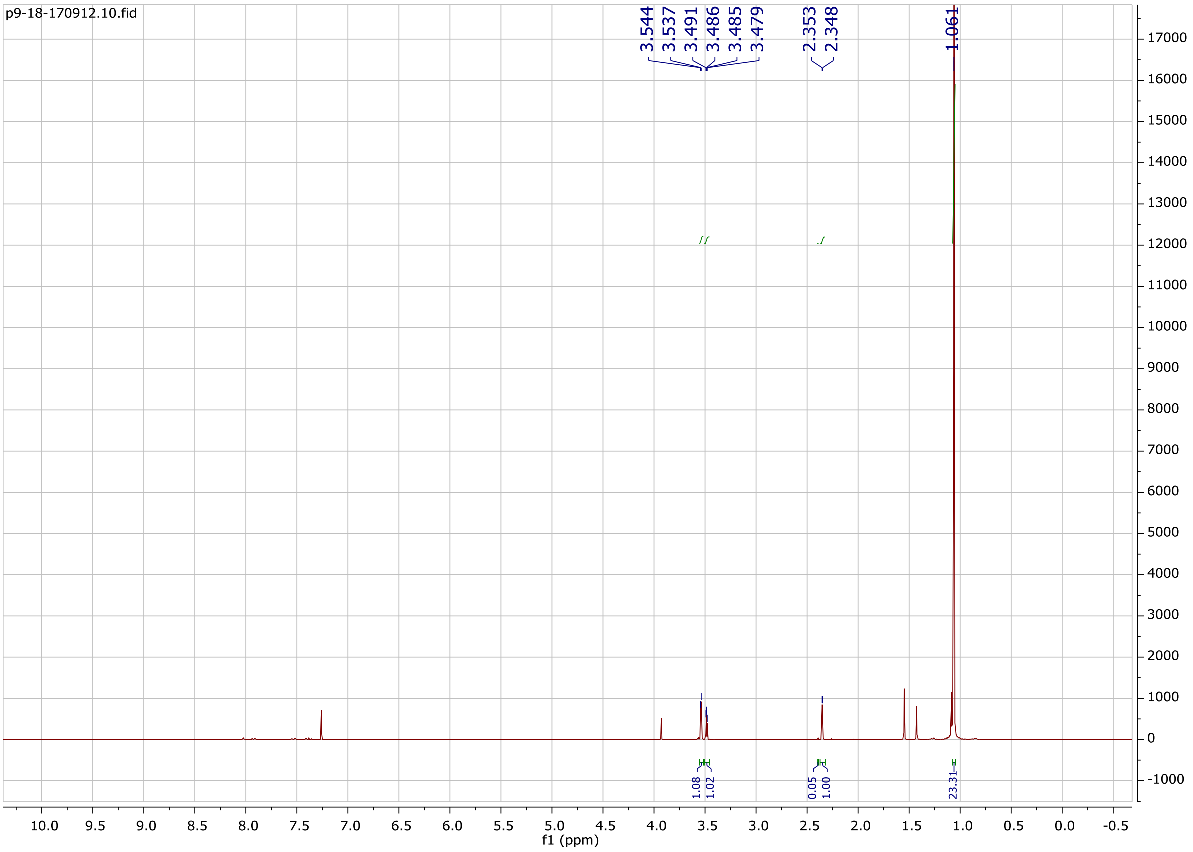

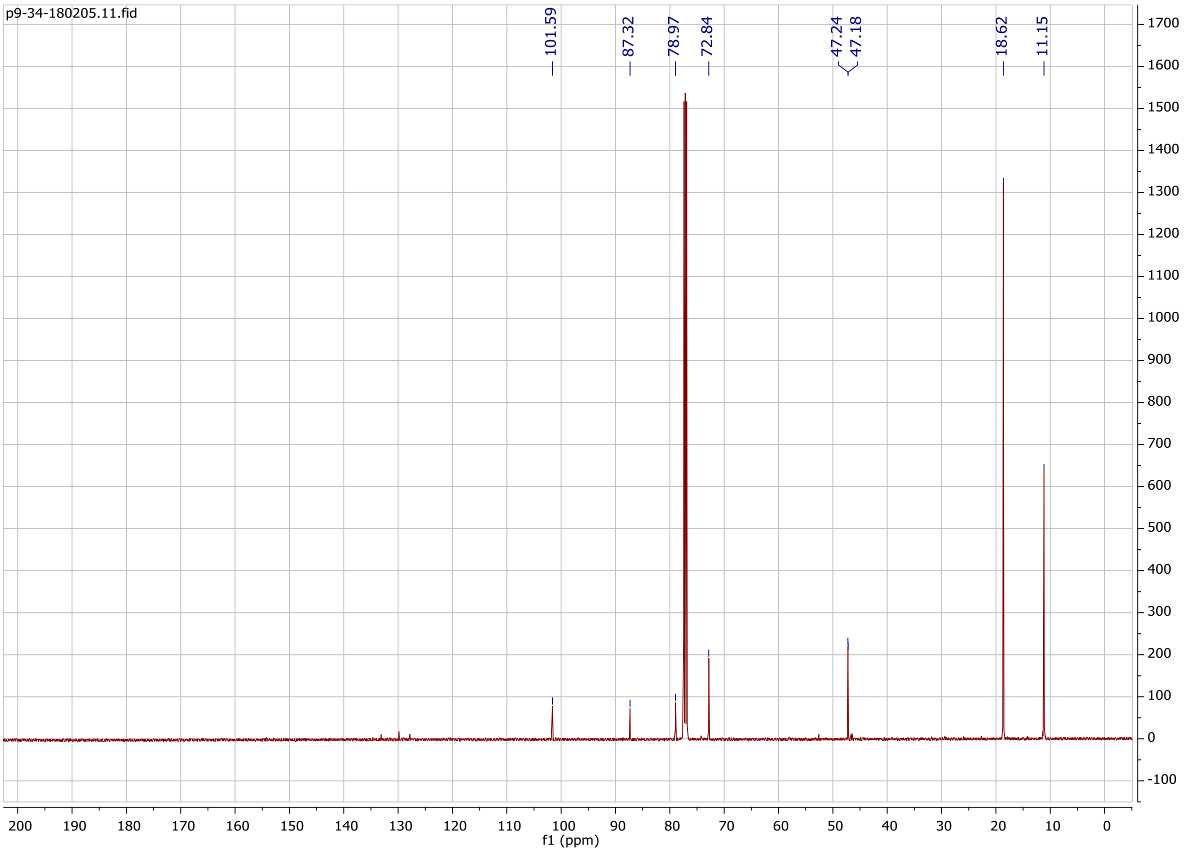

***trans*-2,3-Bis((triisopropylsilyl)ethynyl)oxirane (*trans*-1e)**

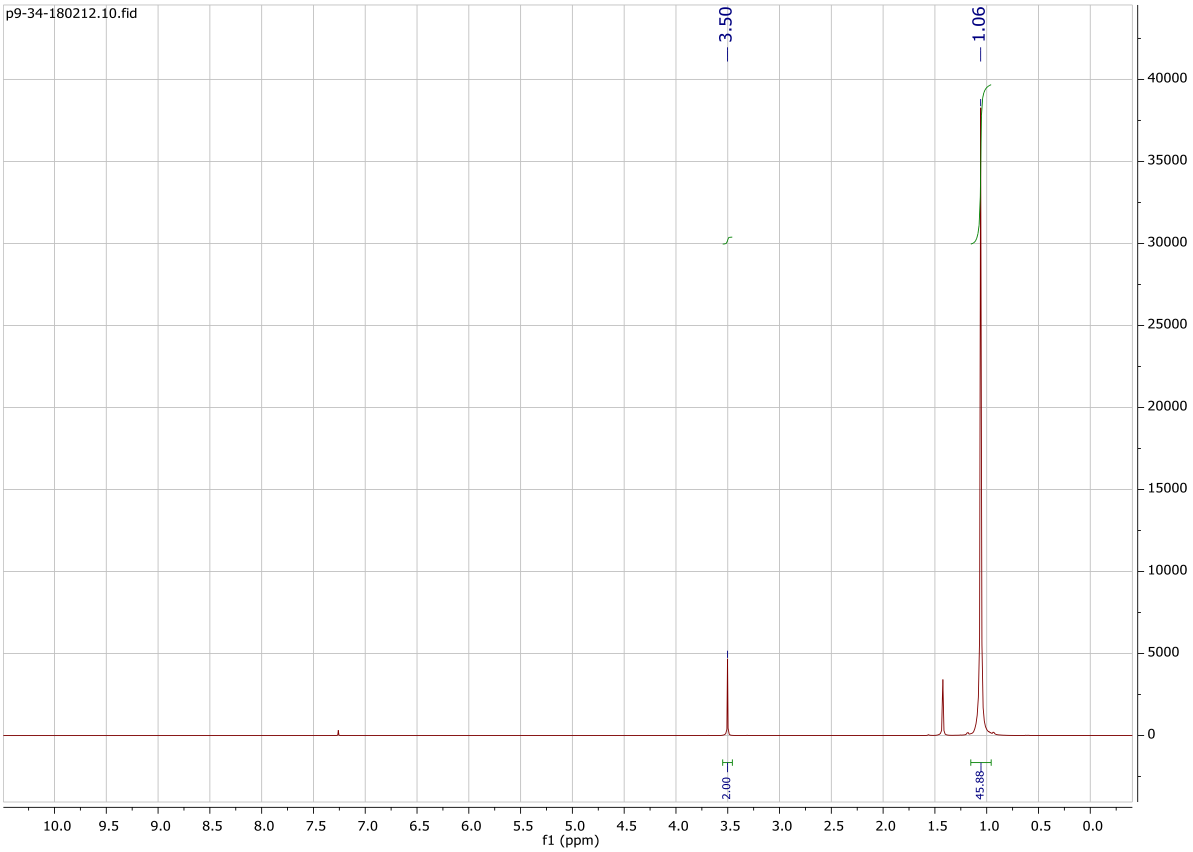

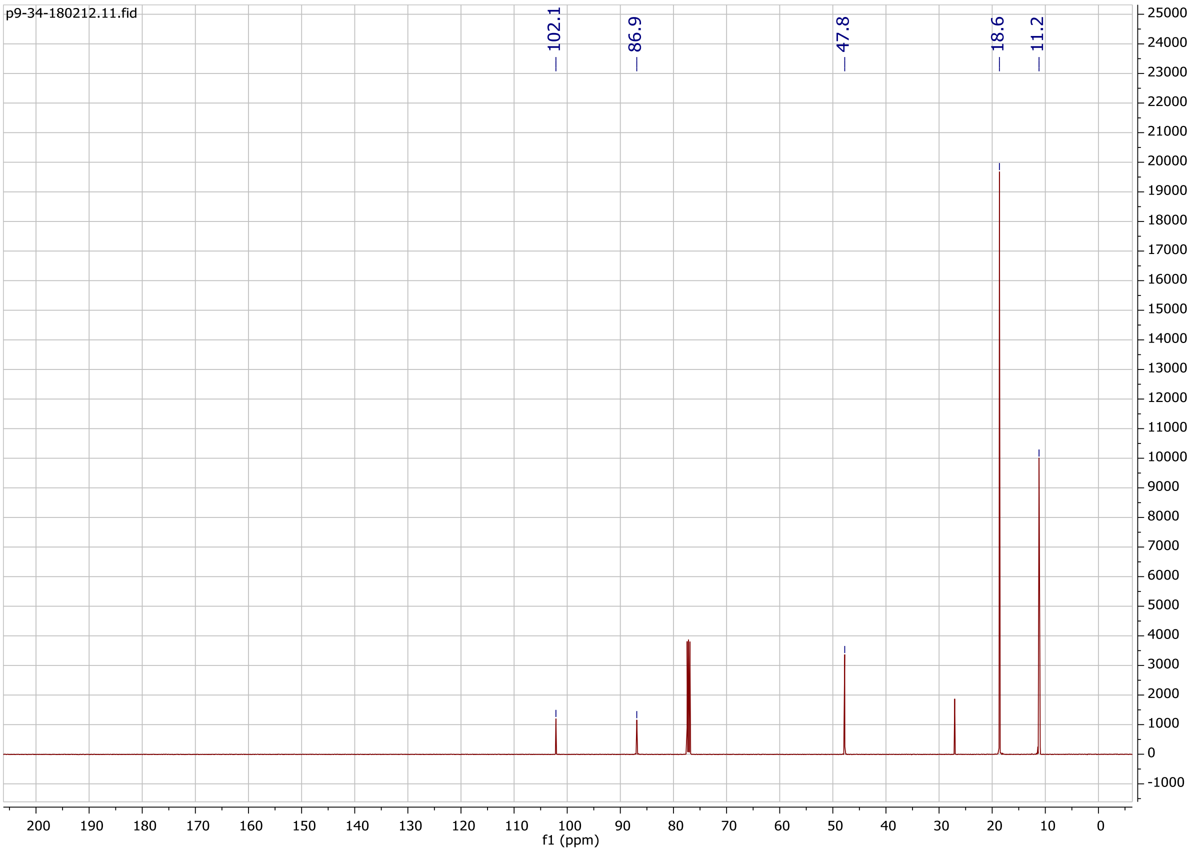

trans-2-Ethyl-3-ethynyl-2-phenyloxirane (W)

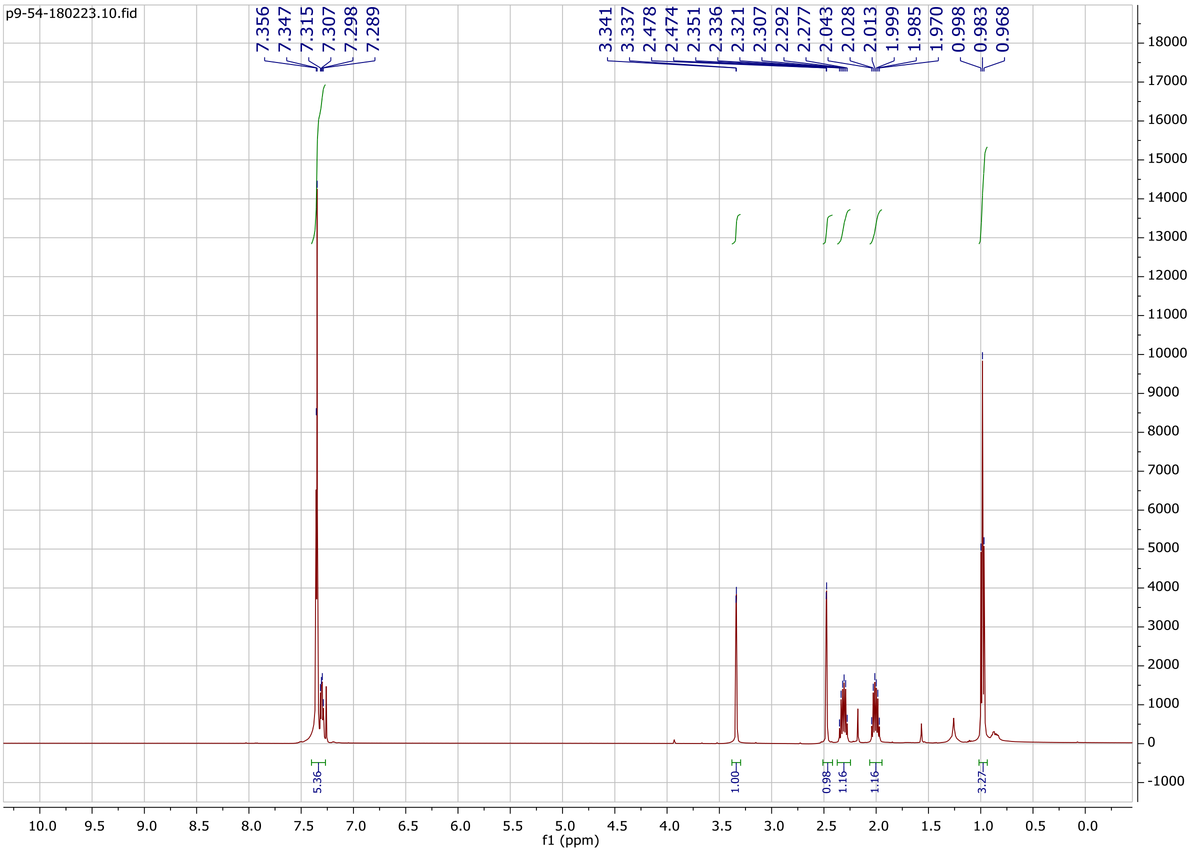

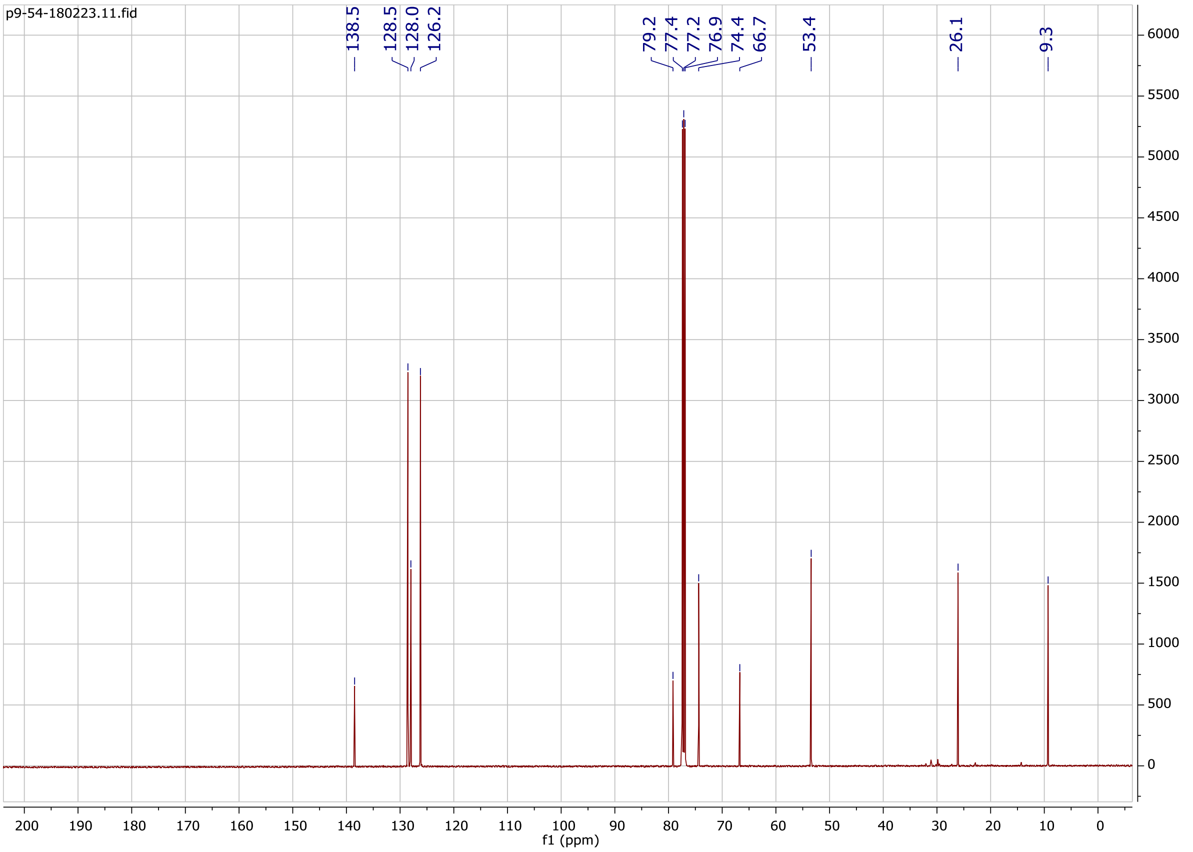

***trans*-((3-Ethyl-3-phenyloxiran-2-yl)ethynyl)triisopropylsilane (*trans*-1f)**

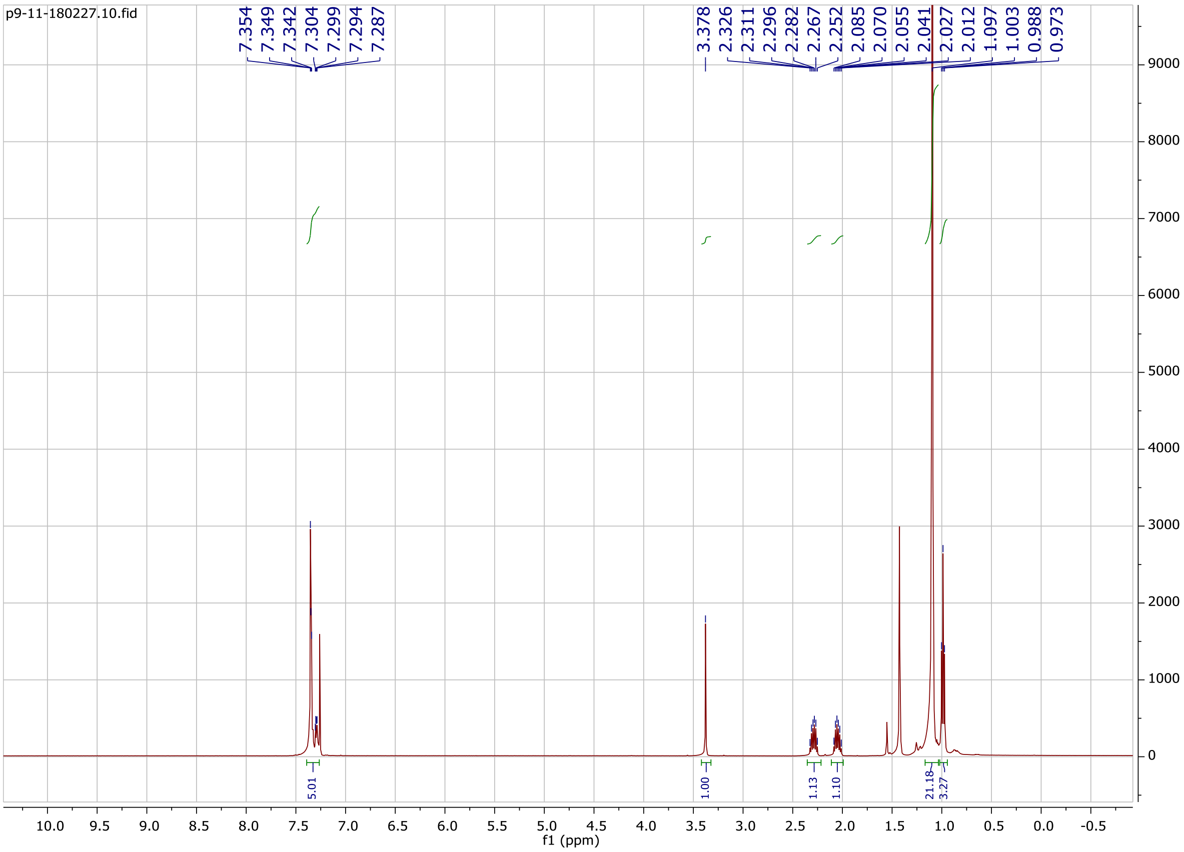

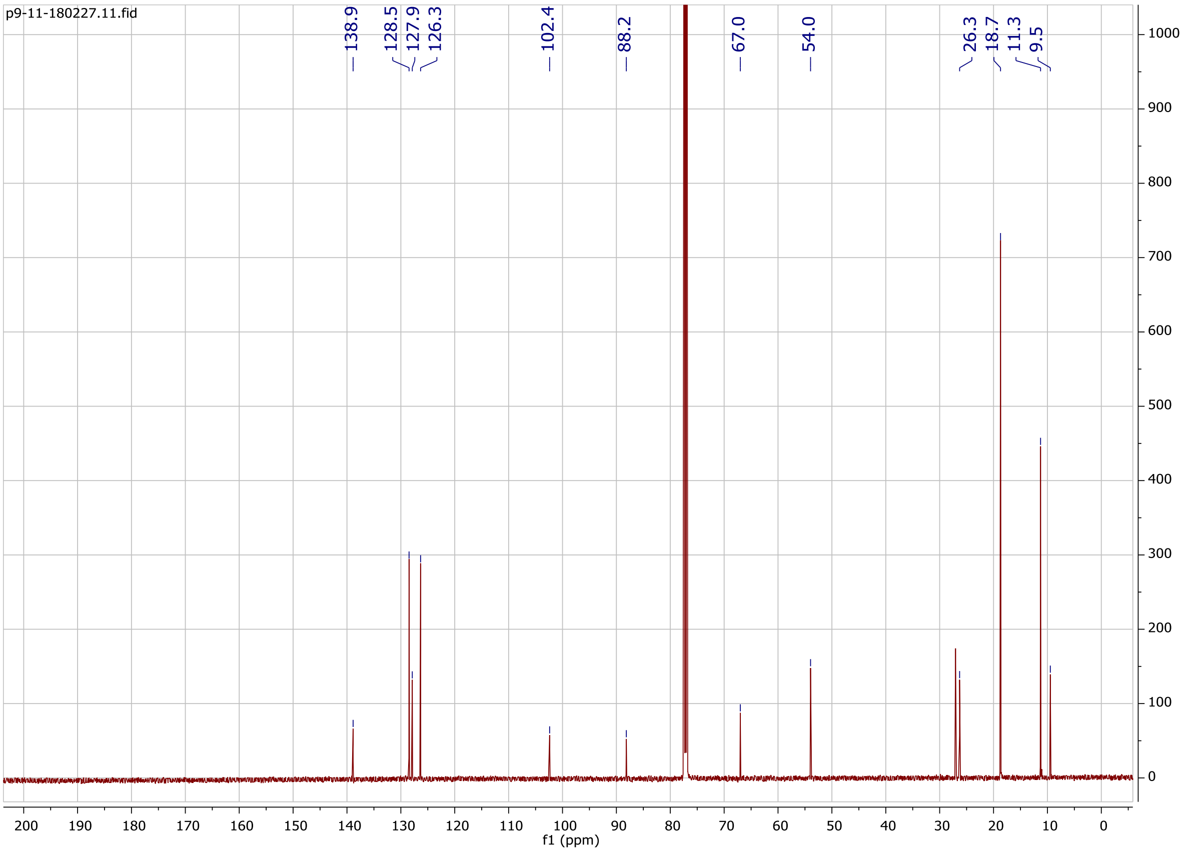

cis-2-Ethyl-3-ethynyl-2-phenyloxirane (Y)

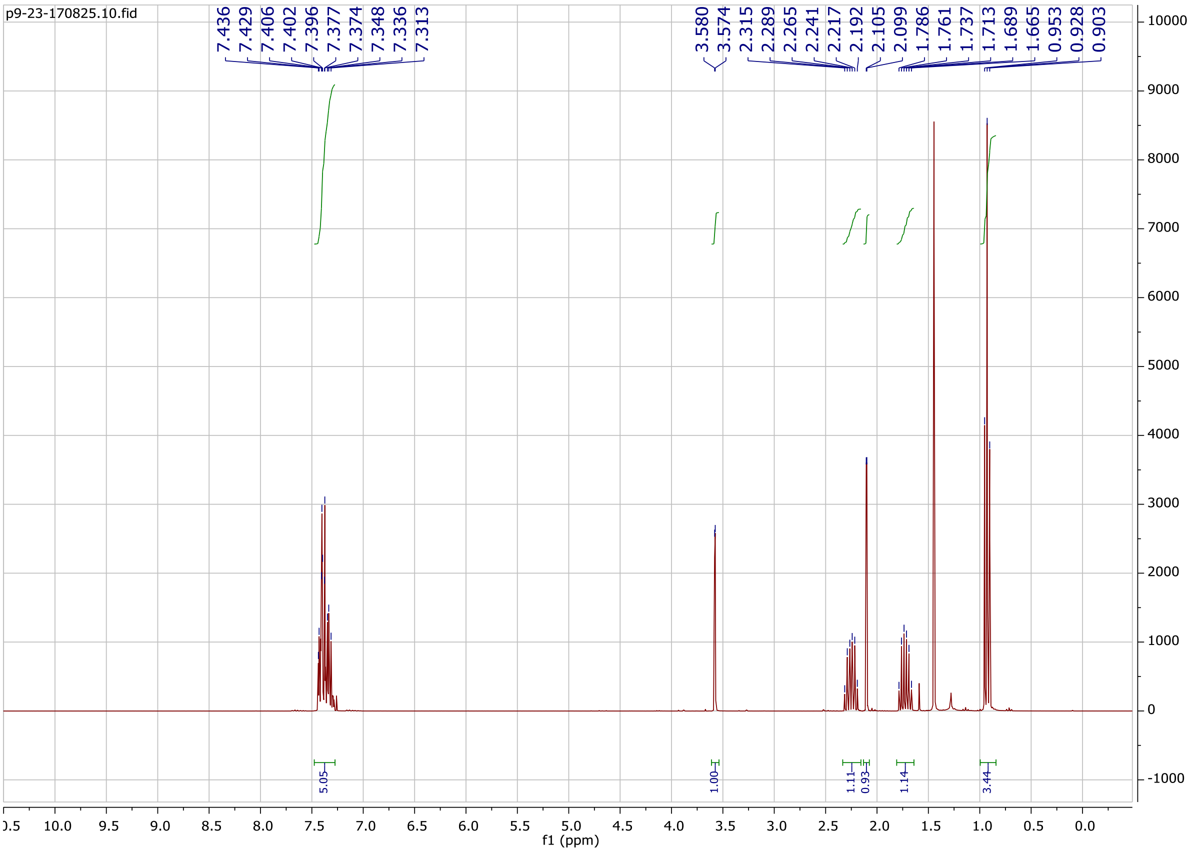

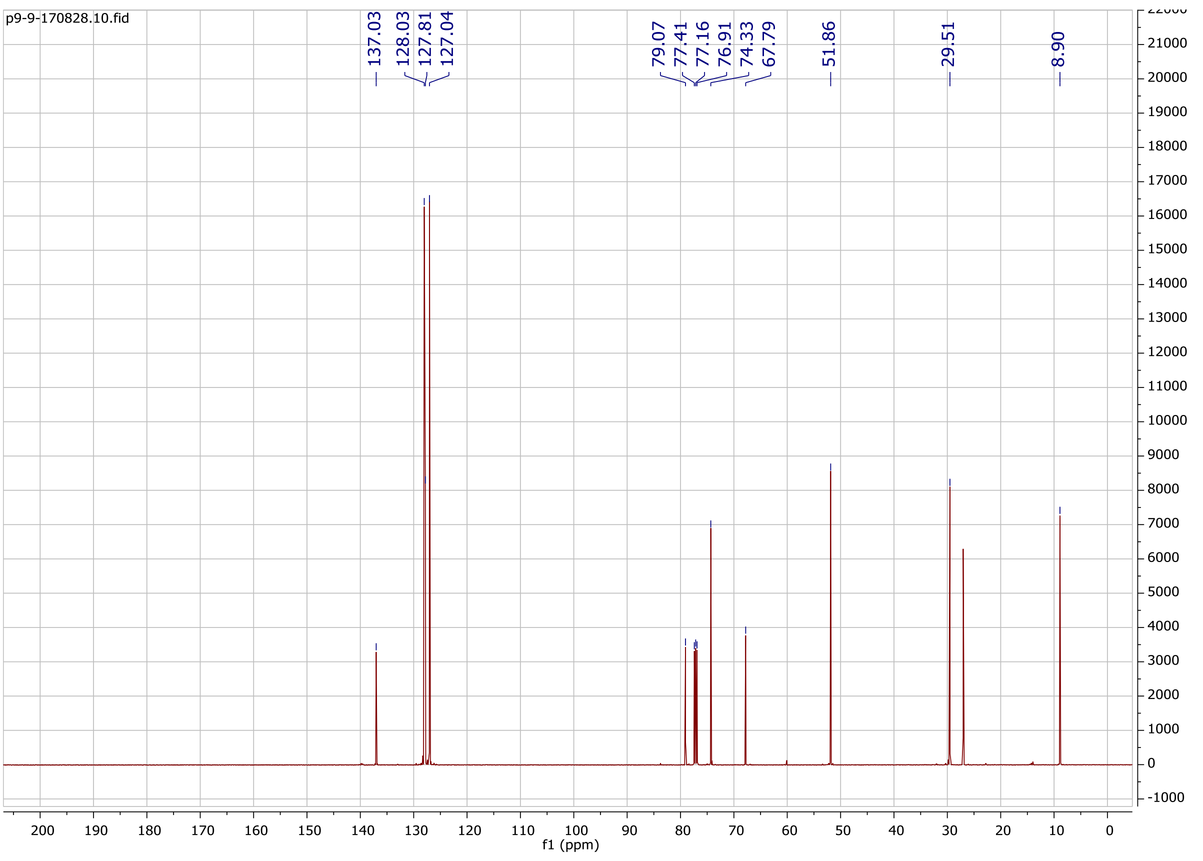

cis-((3-Ethyl-3-phenyloxiran-2-yl)ethynyl)triisopropylsilane (*cis*-1f)

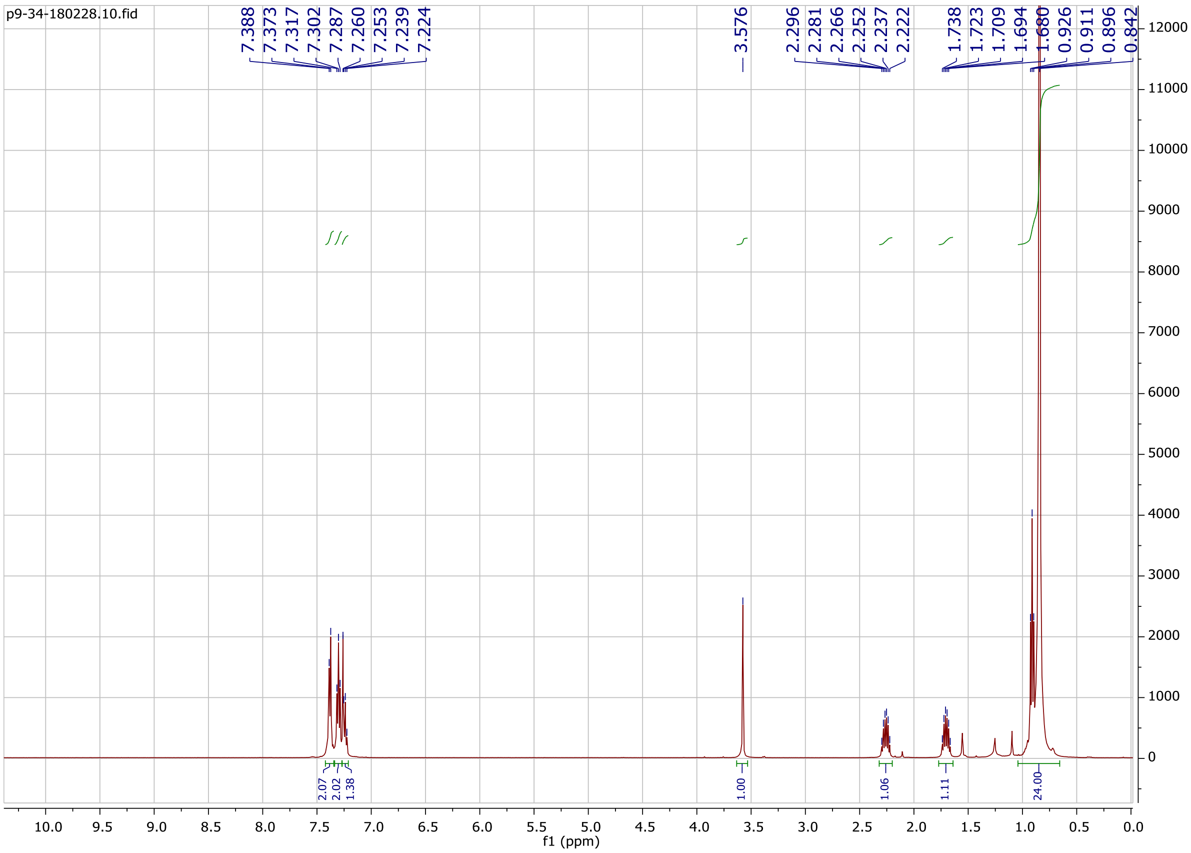

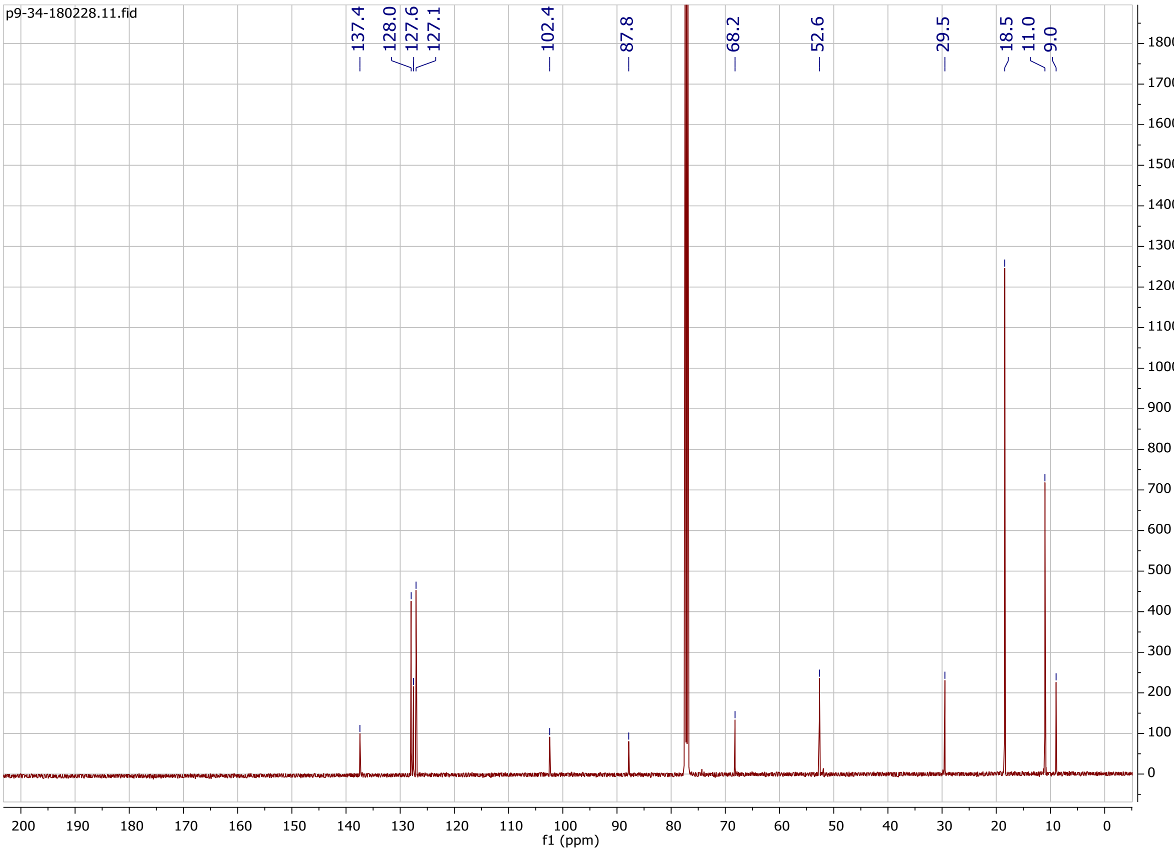

(*Z*)-(3,4-Diphenylbut-3-en-1-yn-1-yl)triisopropylsilane ((*Z*)-11)

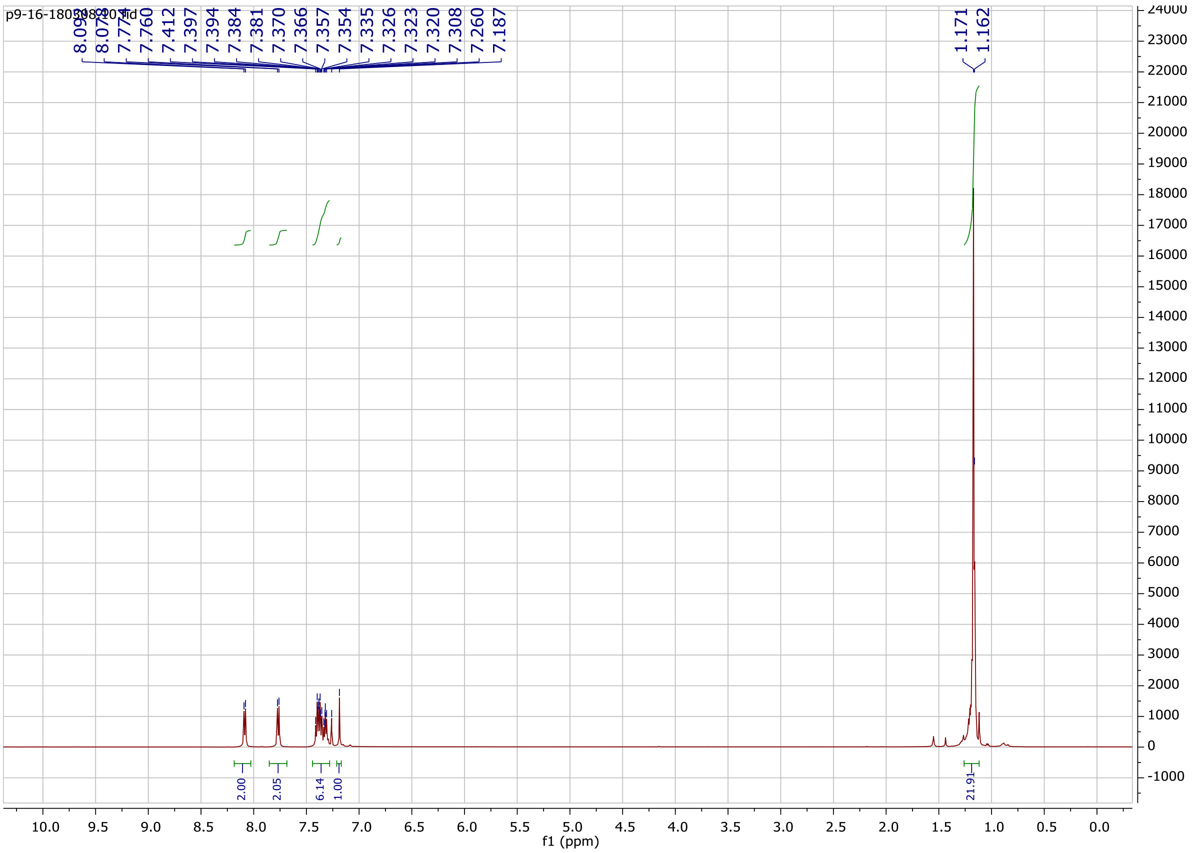

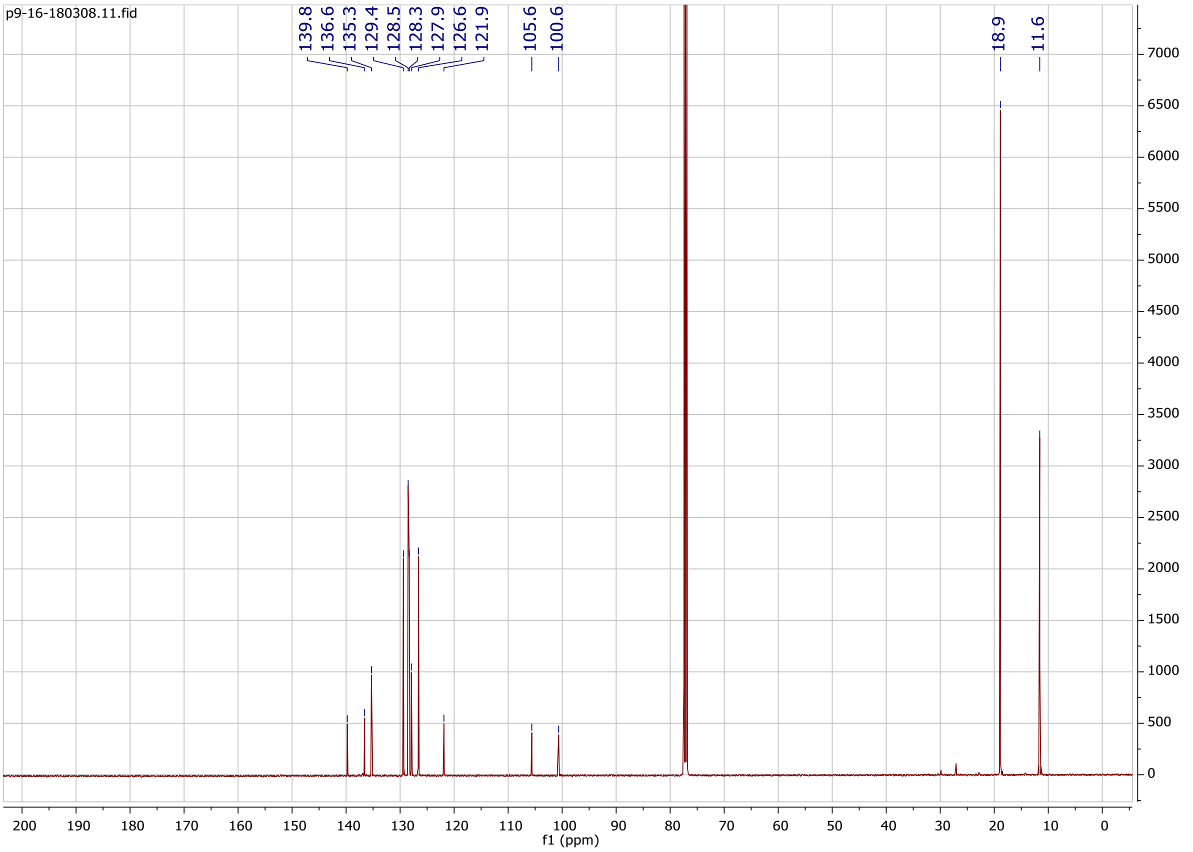

(*E*)-(3,4-Diphenylbut-3-en-1-yn-1-yl)triisopropylsilane ((*E*)-11)

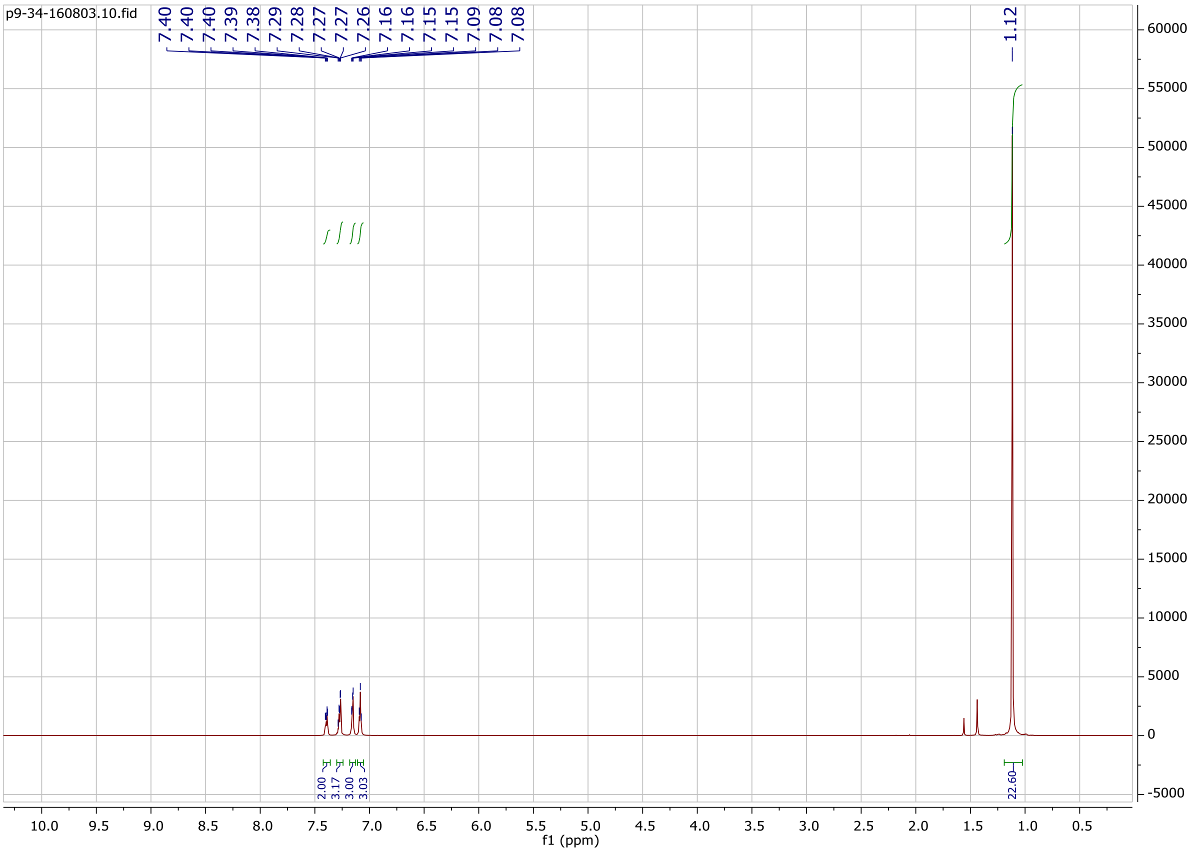

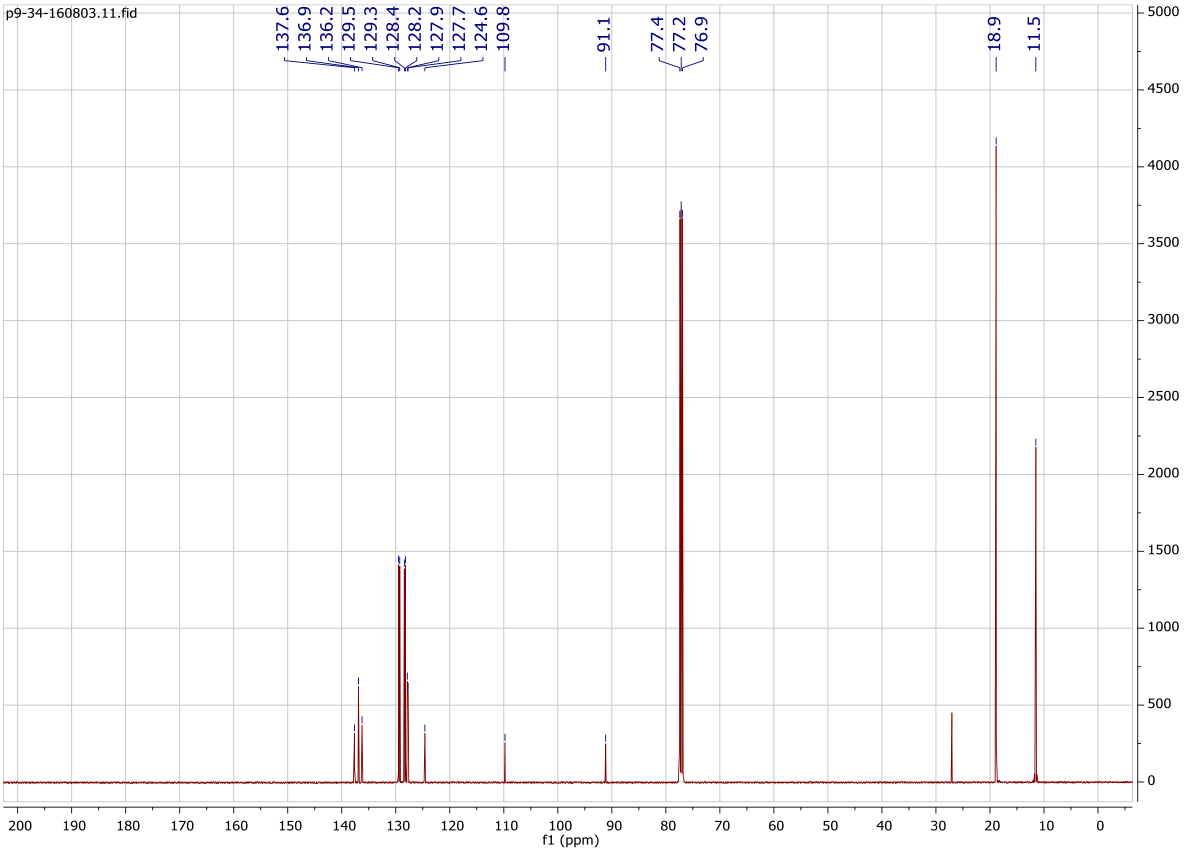

(Z)-Triisopropyl(3-phenyldec-3-en-1-yn-1-yl)silane ((Z)-10)

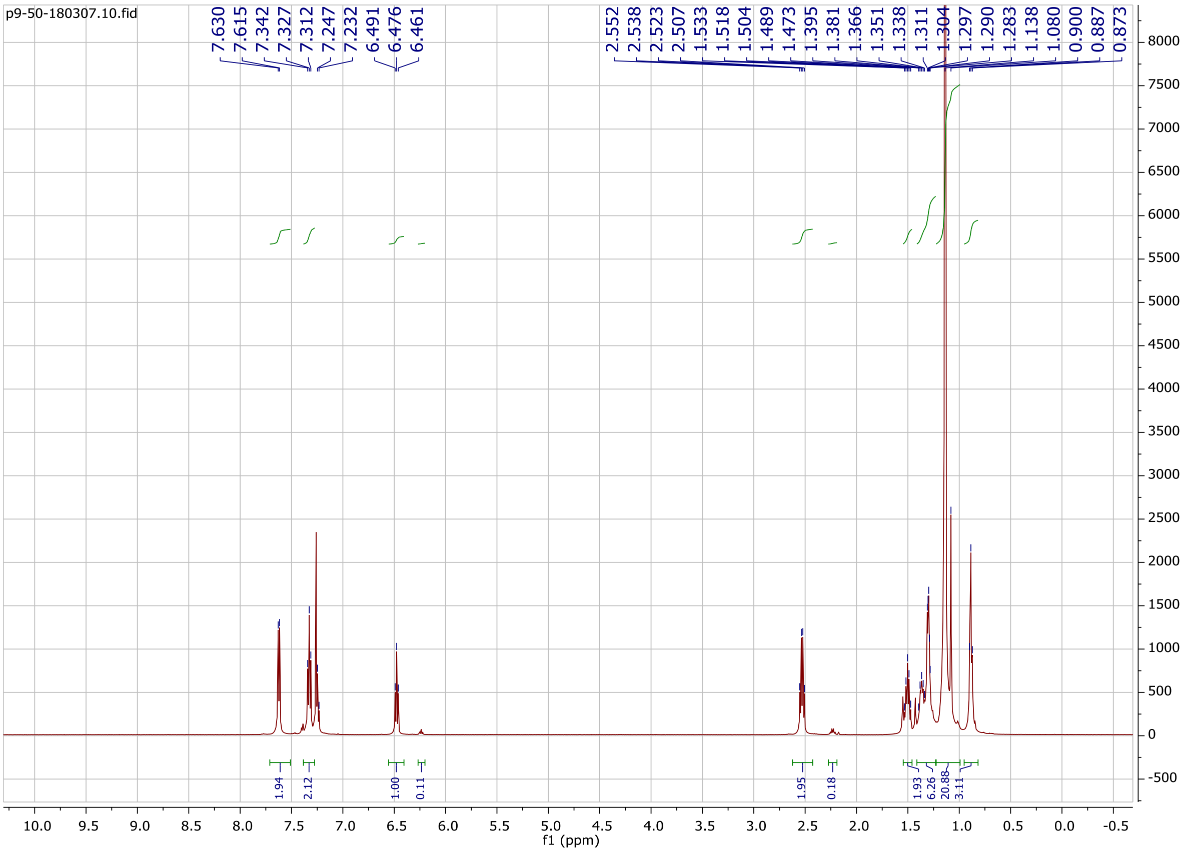

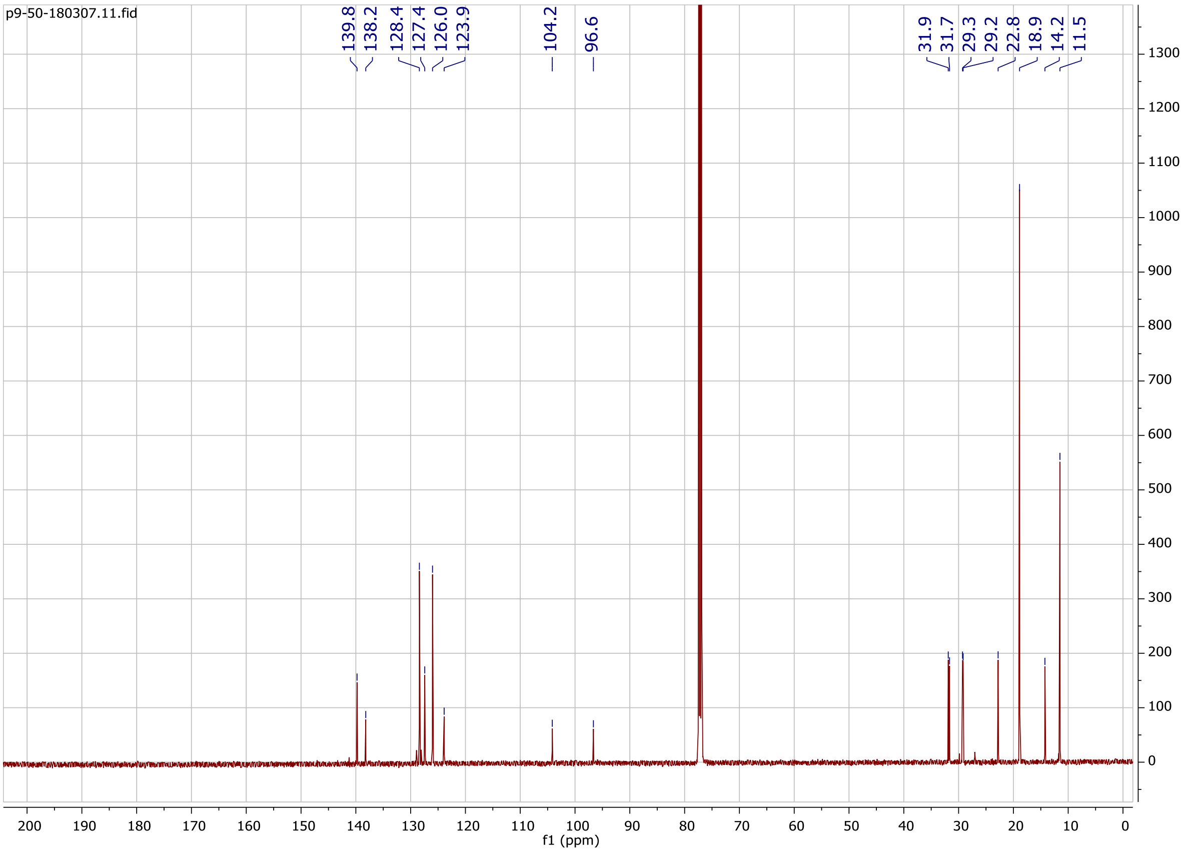

(*E*)-Triisopropyl(3-phenyldec-3-en-1-yn-1-yl)silane ((*E*)-10)

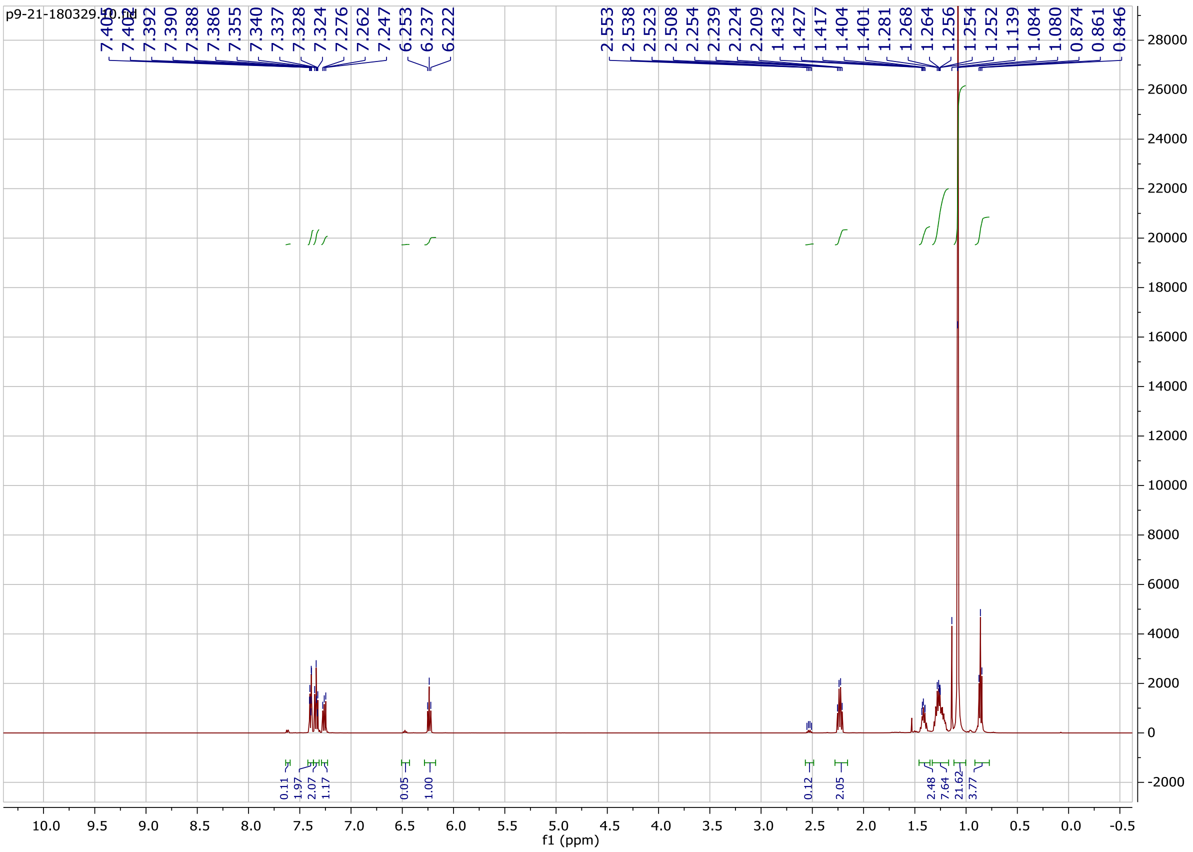

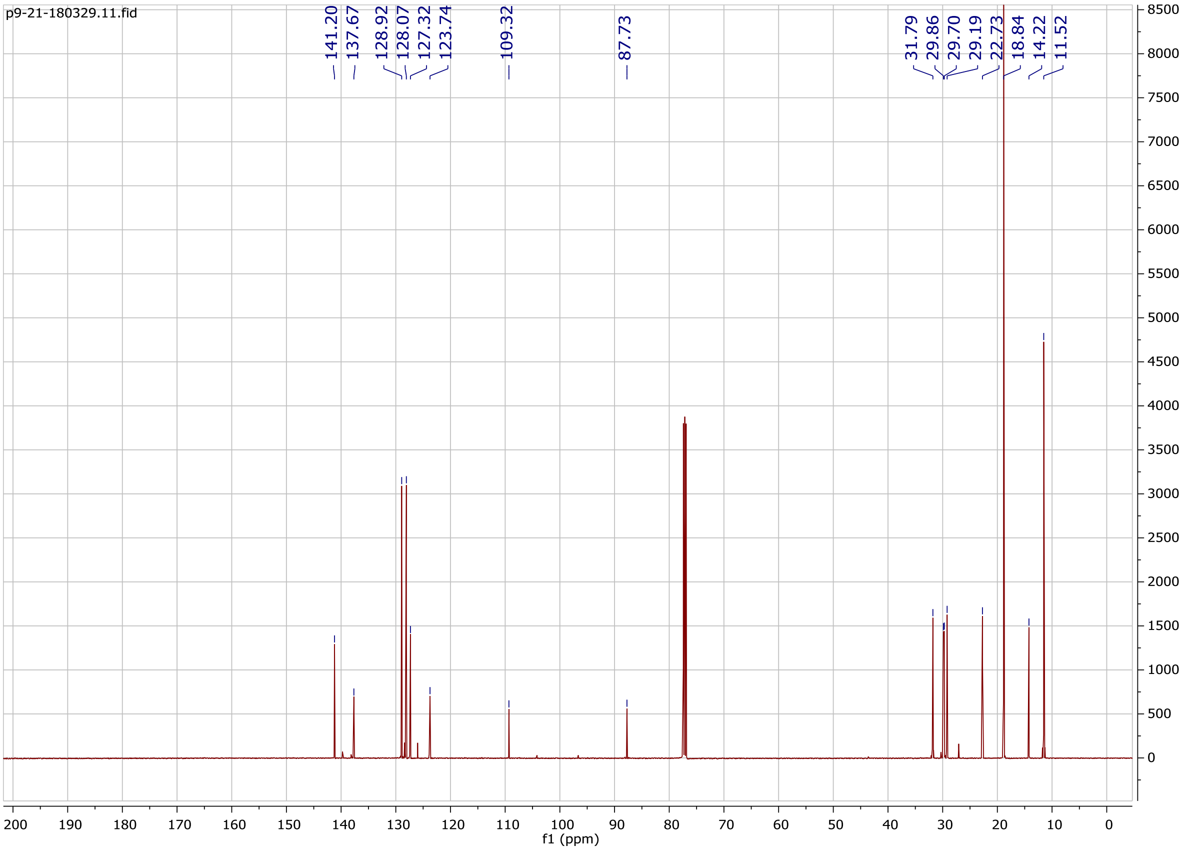

(*Z*)-(3-Phenylhexa-3-en-1,5-diyne-1,6-diyl)bis(triisopropylsilane) ((*Z*)-12)

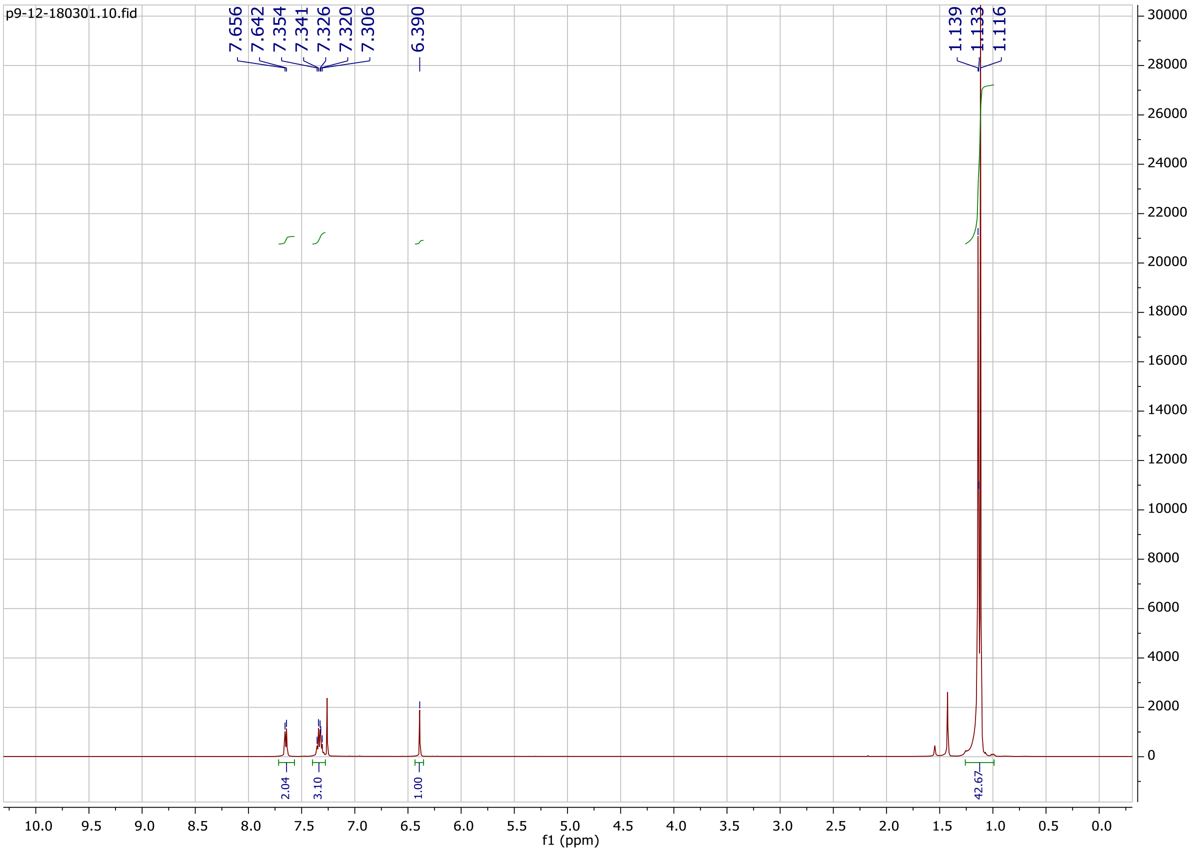

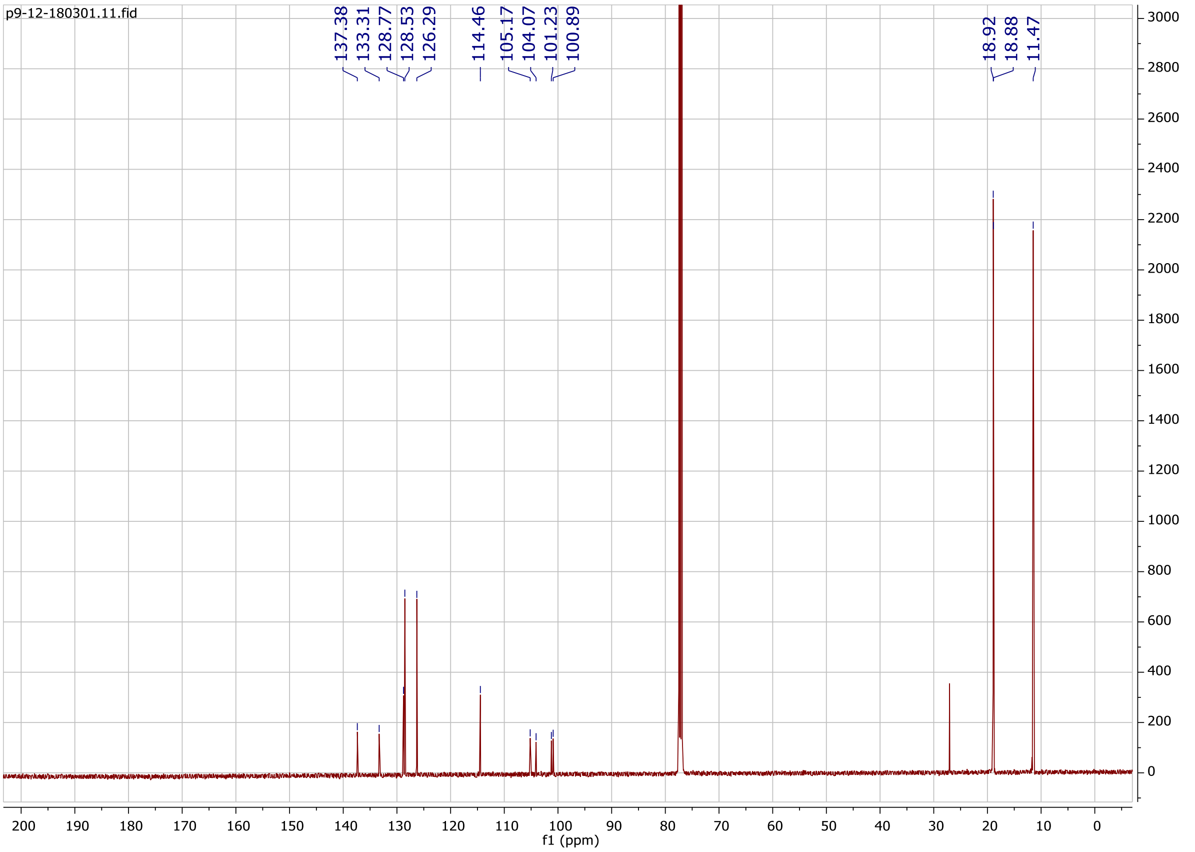

(*E*)-(3-Phenylhexa-3-en-1,5-diyne-1,6-diyl)bis(triisopropylsilane) ((*E*)-12)

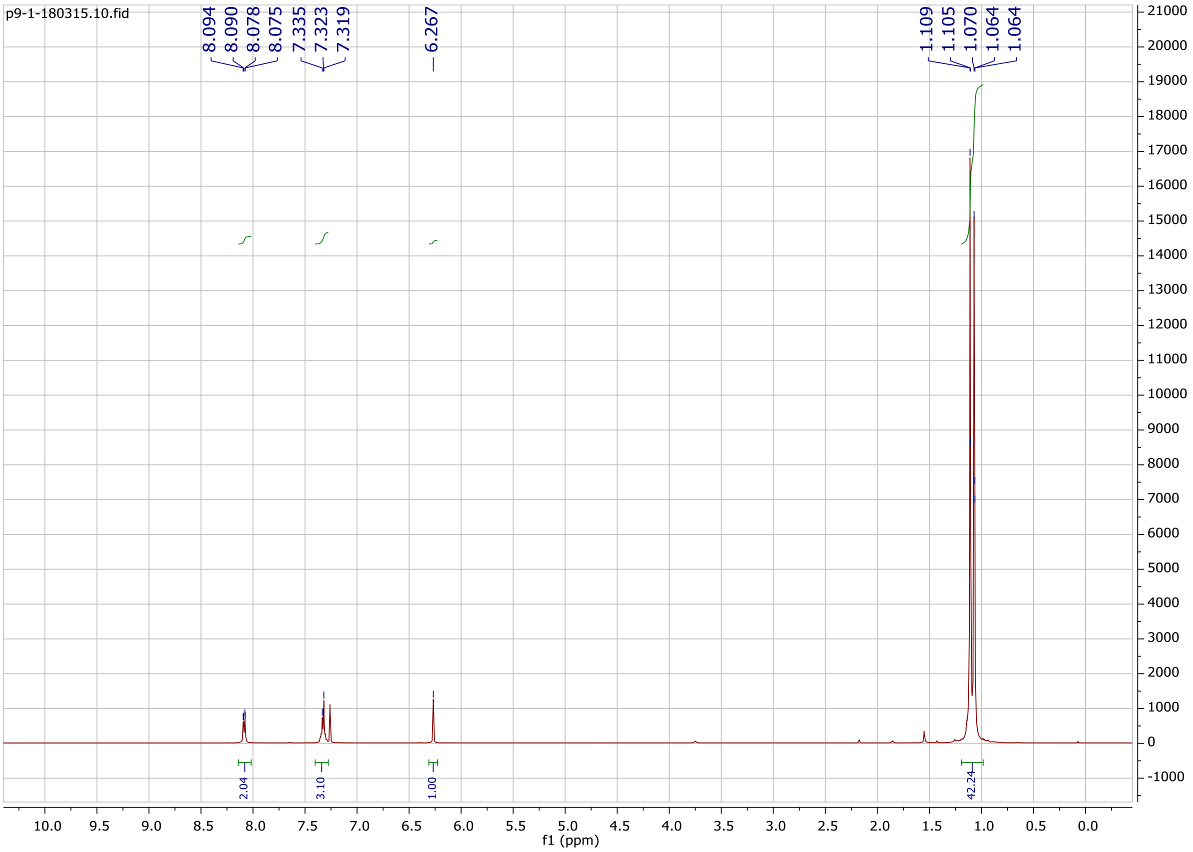

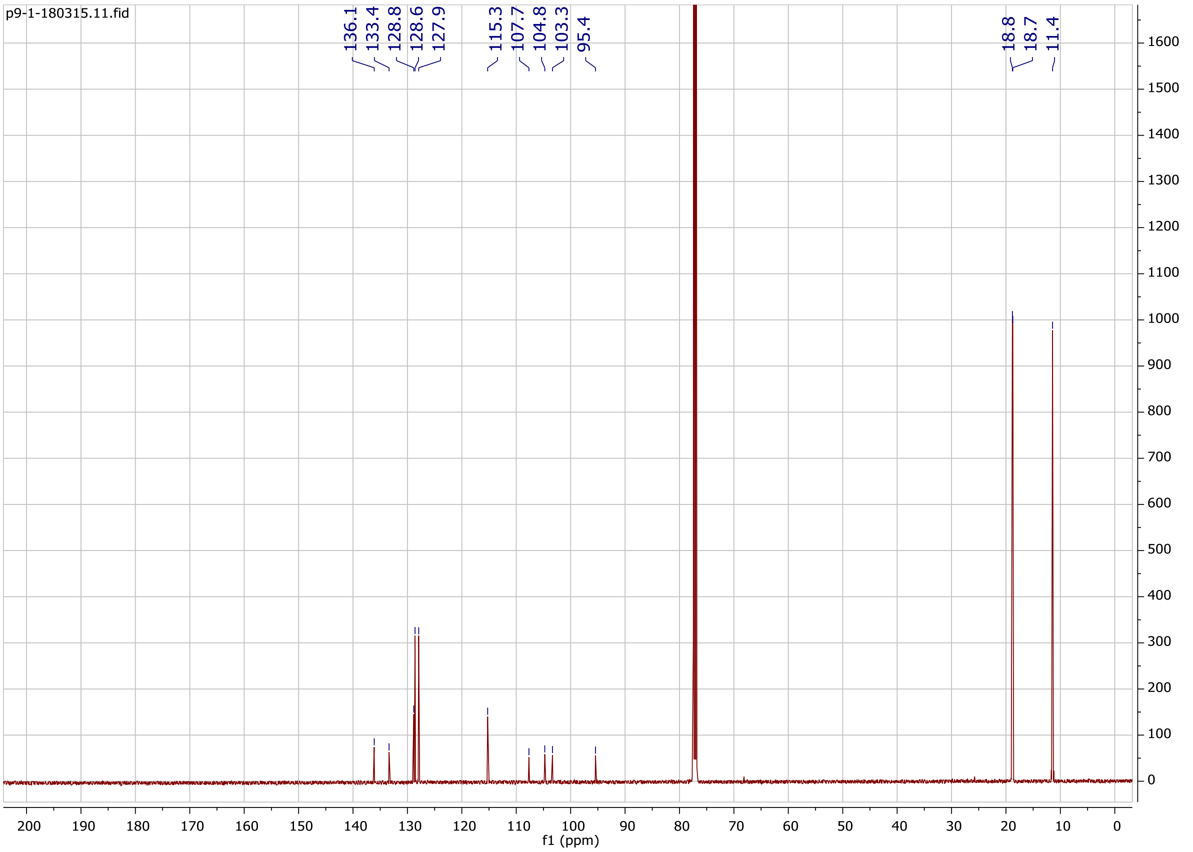

(*Z*)-(3,4-Diphenylhex-3-en-1-yn-1-yl)triisopropylsilane ((*Z*)-13)

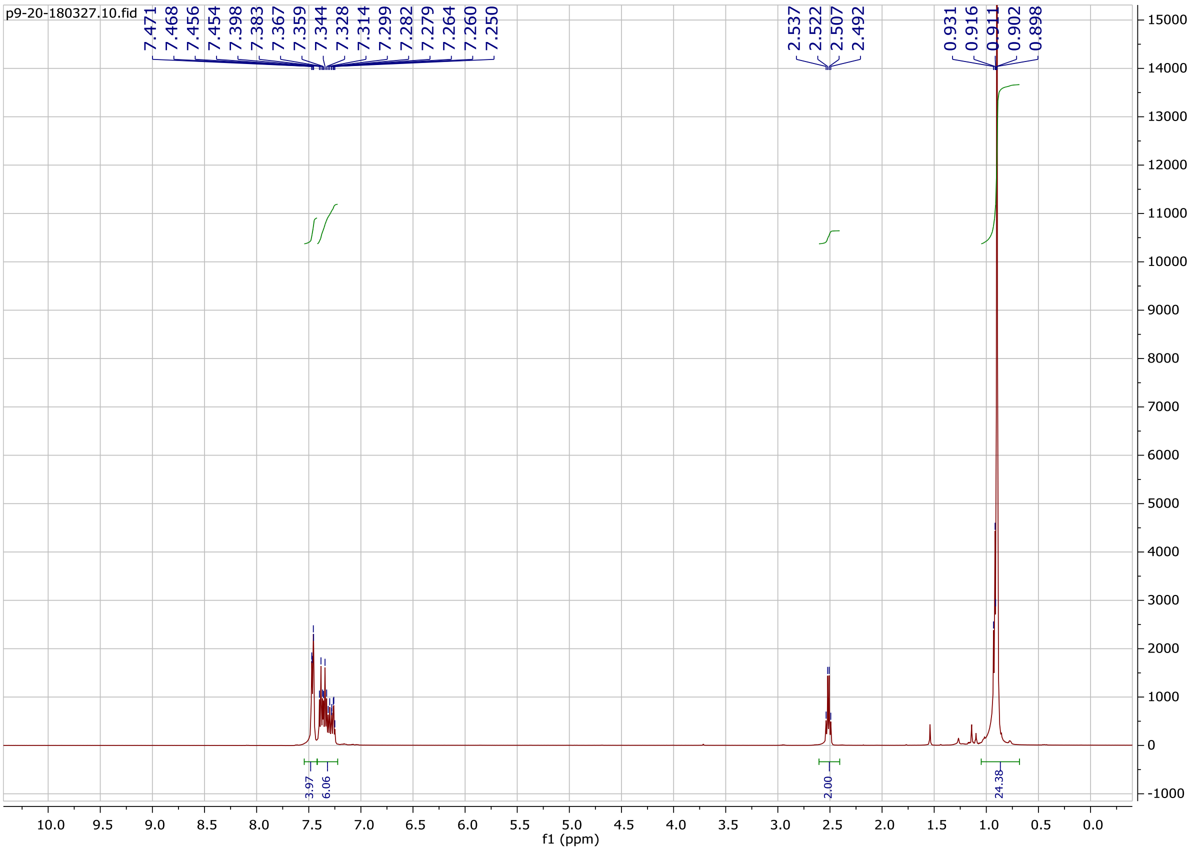

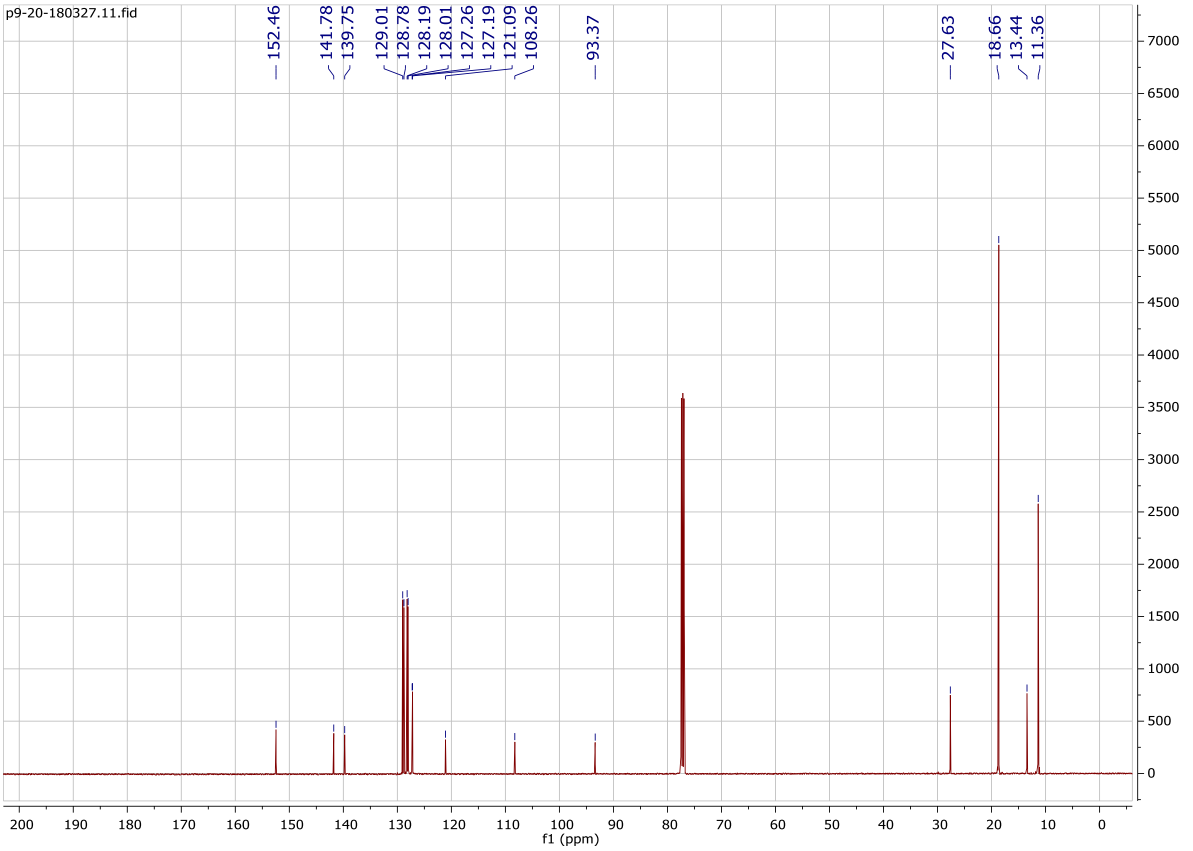

(*E*)-(3,4-Diphenylhex-3-en-1-yn-1-yl)triisopropylsilane ((*E*)-13)

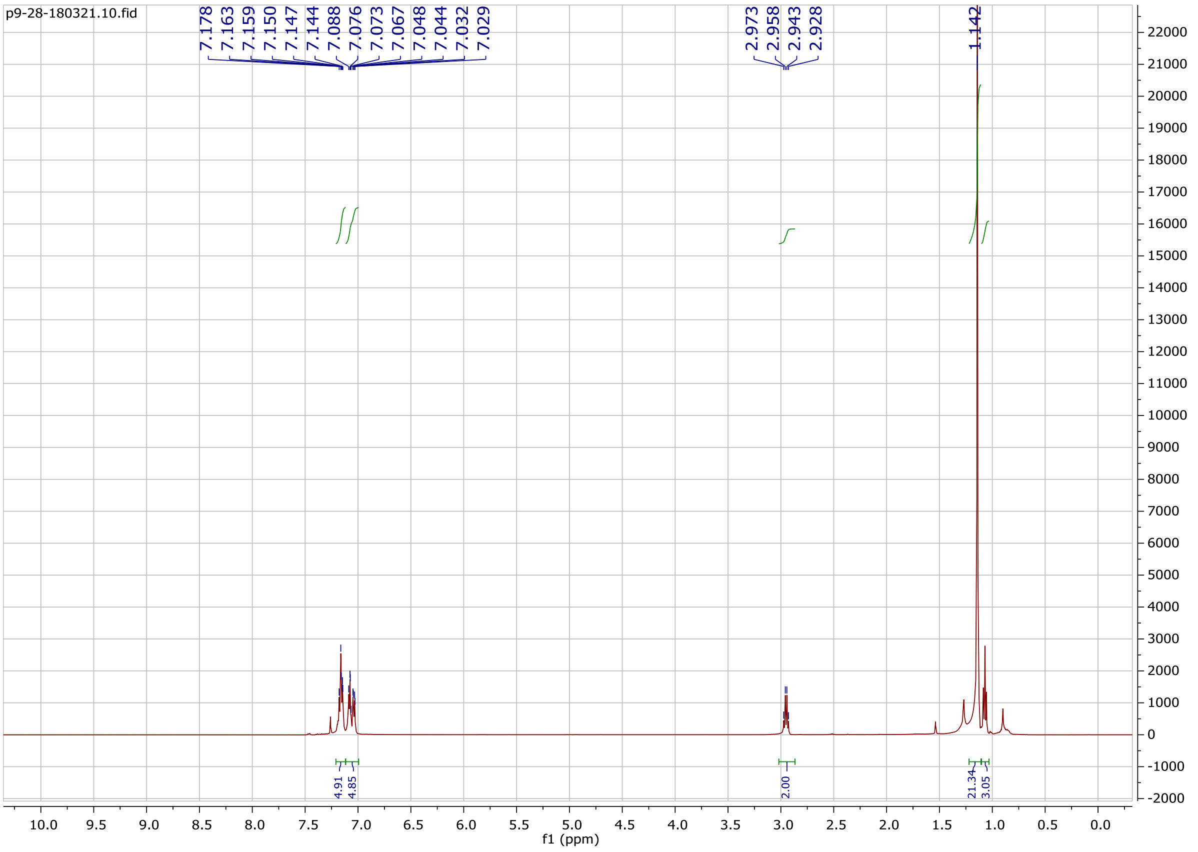

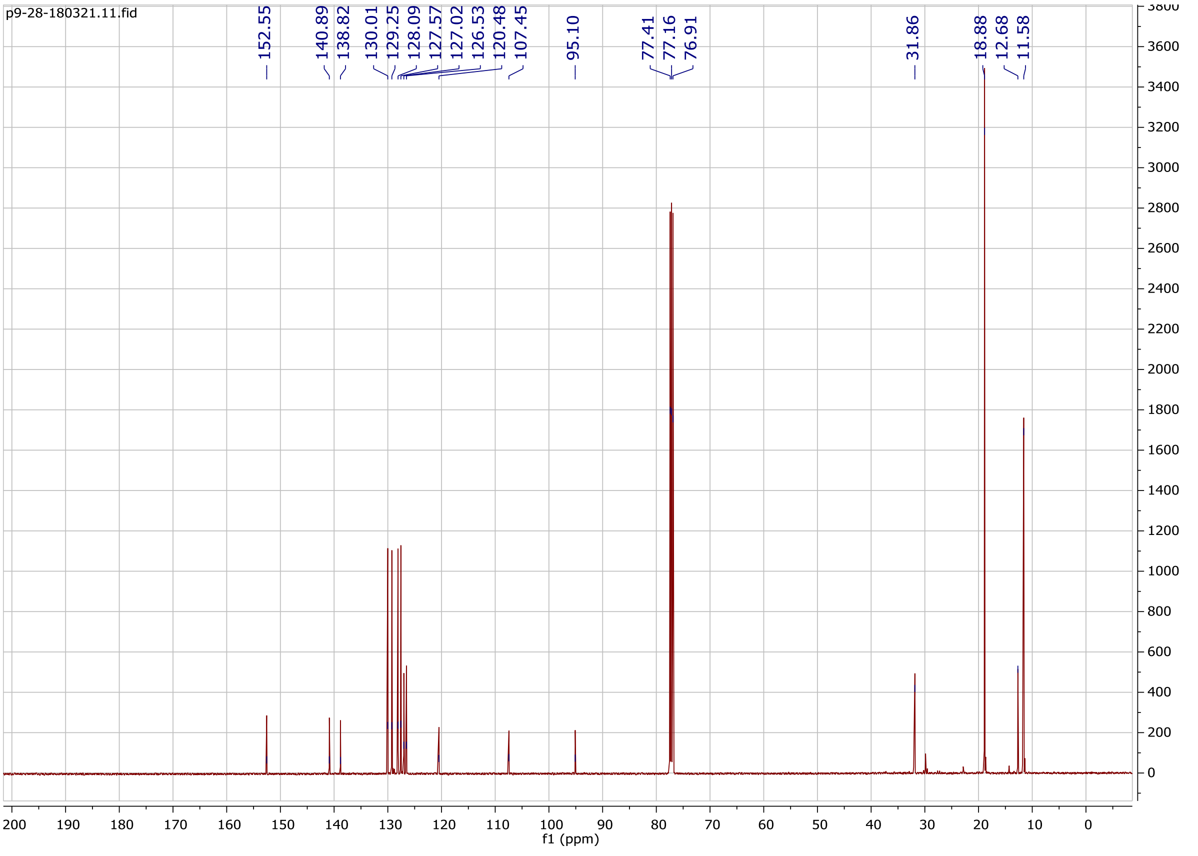

1-((4-Methoxyphenyl)diphenylmethoxy)-5-(triisopropylsilyl)pent-4-yn-2-one (14).

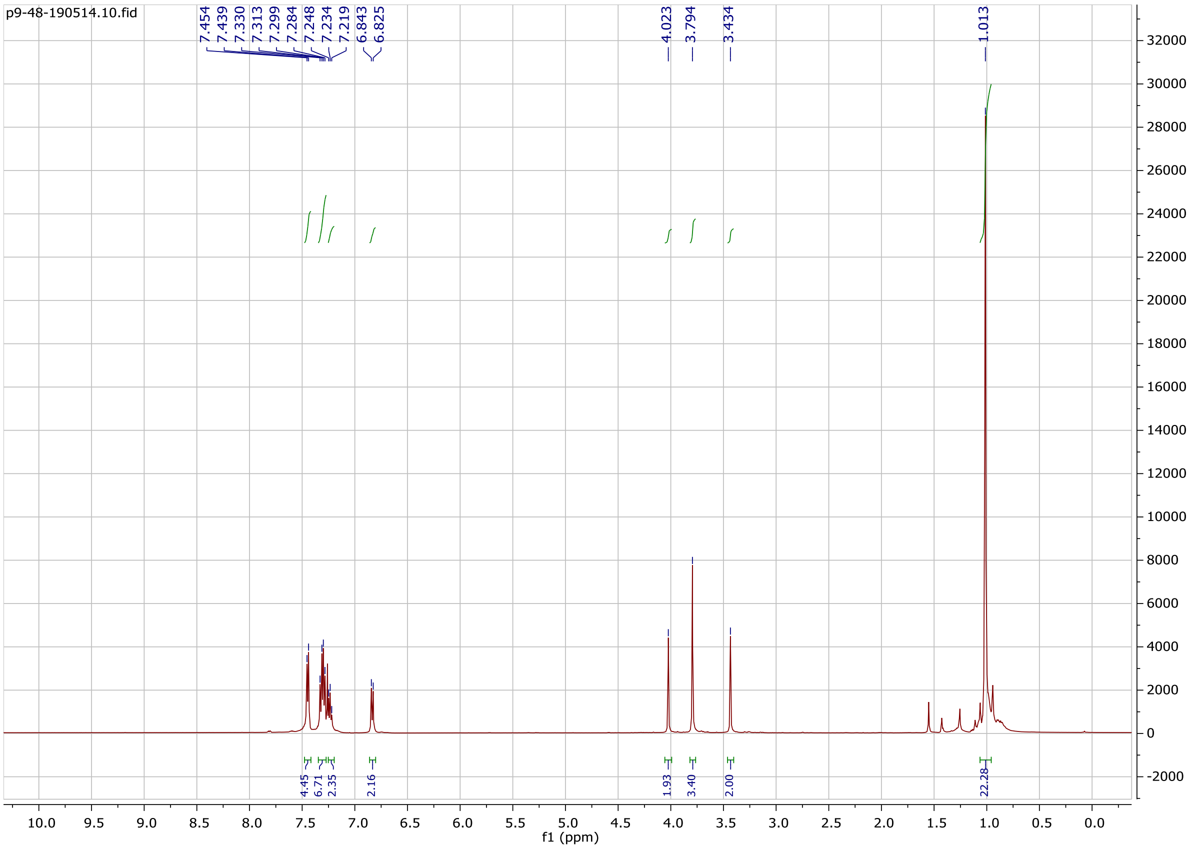

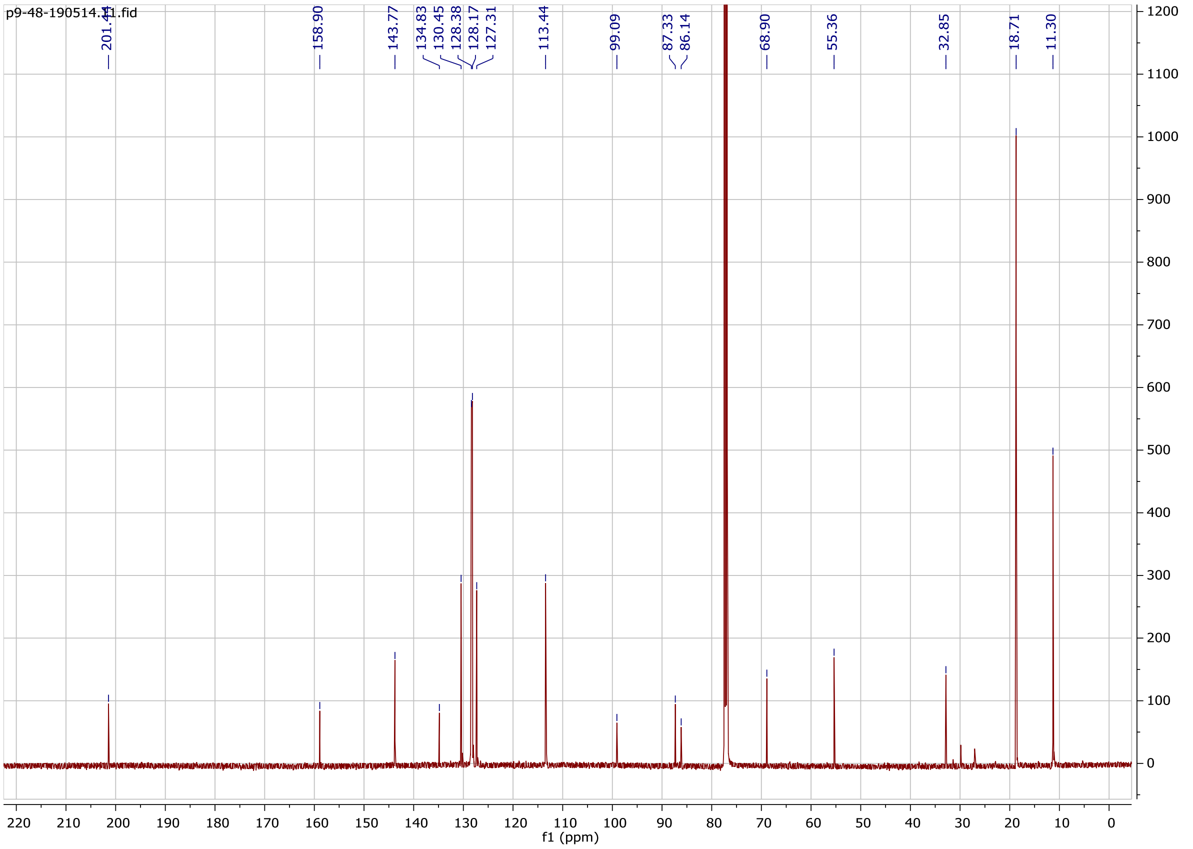

Supplement: File 2 — Copies of 1H NMR and 13C NMR spectra of all new compounds. [file Beilstein_J_Org_Chem-15-1416-s002.pdf]
